# Identification and characterization of soluble binding proteins associated with host foraging in the parasitoid wasp *Diachasmimorpha longicaudata*

**DOI:** 10.1371/journal.pone.0252765

**Published:** 2021-06-17

**Authors:** Juan P. Wulff, Diego F. Segura, Francisco Devescovi, Irina Muntaabski, Fabian H. Milla, Alejandra C. Scannapieco, Jorge L. Cladera, Silvia B. Lanzavecchia

**Affiliations:** Laboratorio de Insectos de Importancia Agronómica, Instituto de Genética Ewald A. Favret (INTA) gv IABIMO (CONICET), Buenos Aires, Argentina; USDA Agricultural Research Service, UNITED STATES

## Abstract

The communication and reproduction of insects are driven by chemical sensing. During this process, chemical compounds are transported across the sensillum lymph to the sensory neurons assisted by different types of soluble binding proteins: odorant-binding proteins (OBPs); chemosensory proteins (CSPs); some members of ML-family proteins (MD-2 (myeloid differentiation factor-2)-related Lipid-recognition), also known as NPC2-like proteins. Potential transcripts involved in chemosensing were identified by an *in silico* analysis of whole-body female and male transcriptomes of the parasitic wasp *Diachasmimorpha longicaudata*. This analysis facilitated the characterization of fourteen OBPs (all belonging to the Classic type), seven CSPs (and two possible isoforms), and four NPC2-like proteins. A differential expression analysis by qPCR showed that eleven of these proteins (CSPs 2 and 8, OBPs 2, 3, 4, 5, 6, 9, 10, and 11, and NPC2b) were over-expressed in female antenna and two (CSP 1 and OBP 12) in the body without antennae. Foraging behavior trials (linked to RNA interference) suggest that OBPs 9, 10, and 11 are potentially involved in the female orientation to chemical cues associated with the host. OBP 12 seems to be related to physiological processes of female longevity regulation. In addition, transcriptional silencing of CSP 3 showed that this protein is potentially associated with the regulation of foraging behavior. This study supports the hypothesis that soluble binding proteins are potentially linked to fundamental physiological processes and behaviors in *D*. *longicaudata*. The results obtained here contribute useful information to increase the parasitoid performance as a biological control agent of fruit fly pest species.

## Introduction

Chemical sensing plays a fundamental role in insect communication and reproduction, assisting behaviors such as host-seeking, feeding, mating, and avoidance of toxic substances [1]. Chemical compounds enter through pores in the sensilla cuticle and are then transported through the lymph to sensory neurons assisted by water-soluble binding proteins [1]. Different groups of such proteins have been described in insects: odorant-binding proteins (OBPs); chemosensory proteins (CSPs); protein members of the ML (MD-2 (myeloid differentiation factor-2)-related Lipid-recognition) family and odorant-degrading enzymes (ODEs) [1, 2]. These binding proteins form a complex with odor molecules, which further interact with membrane-bound receptors on the sensory neurons, such as odorant receptors (ORs), ionotropic receptors (IRs), their corresponding co-receptors (ORco and IRco), gustatory receptors (GRs), and sensory neuron membrane proteins (SNMPs) [1].

After the first OBP was discovered in *Antheraea polyphemus* Cramer (Lepidoptera: Saturniidae) [3], OBP homologous genes have been identified in many insect orders ranging from 12 to 100 identified proteins per species [4]. Members of this family can be grouped in more than one category, such as Classic OBP, Minus-C type, and Plus-C type [5]. The Classic OBP type (from which other variants can be defined) is a water-soluble protein of about 120–150 amino acids (10–30 kDa), expressed at a high concentration (10–20 mM) and with six highly conserved cysteine residues in a characteristic position [6]. These cysteines form three disulfide bonds to stabilize a tertiary structure formed by six or seven α-helices that define a hydrophobic binding cavity, which may undergo changes in a pH-dependent manner [7]. CSPs are very similar to OBPs in structure and function but characterized by four conserved cysteine residues forming two disulfide bonds to stabilize five or six α-helices [8]. The first CSP was discovered in *Drosophila melanogaster* Meigen (Diptera: Drosophilidae) showing high expression in the olfactory sensilla of antennae [9]. This suggested a potential olfactory function, later confirmed by semiochemical binding and electro-antennogram assays [10, 11]. The number of genes for CSPs ranges from 4 to 70 per species [4], and there is no classification into subfamilies.

The MLs are also water-soluble proteins, sharing several characteristics with CSPs and OBPs [12]. These proteins (also known as NPC2-like proteins) possess a conserved domain that can be found in about 100 protein families, such as myeloid differentiation factor 2 (MD-2), Niemann-Pick disease protein type C2 (NPC2), and Ecdysteroid-regulated 16 kDa protein (ESR16). NPC2-like proteins have been identified in different sensory structures in insects [12–14], and their function as semiochemical carriers was confirmed in some species [15, 16]. NPC2s are the most widely studied ML proteins in insects and can be classified into three sub-families, depending on the number of cysteine residues: six, seven, or eight [17]. The number of NPC2 genes ranges from three to fourteen per species [4]. However, in some cases, the same orthologous sequence has been classified into different subgroups (NPC2, ESR16 or MD-2), in different Hymenoptera species [18, 19].

Recent studies have confirmed other important roles for soluble binding proteins in insect biological processes. OBPs are also involved in metabolic functions, reproduction, or as a component of the parasitic wasp venom [20–22]. CSPs are also associated with molting, mating, and development [23, 24]. Some NPC2-like proteins are linked to molting as well as intracellular transport of sterols and spermatogenesis [25–27]. This supports the use of these genes as potential targets to manipulate some fundamental behaviors at the species level [*e*.*g*. 28].

Parasitic wasps are important natural enemies of numerous species of insects, several of which have achieved pest status in agricultural systems. Consequently, some parasitoid species are important biological control agents used in integrated pest management programs [29]. Parasitic wasps find their hosts in the foraging environment by primarily using chemical cues [30]. Recent studies on soluble binding proteins in parasitoids, such as *Microplitis mediator* Haliday (Hymenoptera: Braconidae) and *Anastatus japonicus* Ashmead (Hymenoptera: Eupelmidae), point to these genes/proteins as potential targets for manipulating fundamental behaviors [31, 32]. Although OBP studies are predominant, research on CSPs and NPC2-like proteins has increased [12, 33]. This opens the possibility of exploring their genes /proteins to obtain efficient biological control agents, as an alternative to the use of chemical treatments against insect pest species [34].

*Diachasmimorpha longicaudata* Ashmead (Hymenoptera: Braconidae) is an endoparasitoid wasp considered the main biological control agent of Tephritidae fruit flies of economic importance. Females parasitize late instar larvae of several fruit fly species, such as *Ceratitis capitata* Wiedemann and *Anastrepha fraterculus* Wiedemann (Diptera: Tephritidae), which are important pests in several South American countries. Numerous ecological and behavioral studies have addressed key issues about the foraging behavior *of D*. *longicaudata* [35, 36], such as the identification of volatile organic compounds (VOCs) that are significantly attractive to females [37]. However, there are no studies on molecular mechanisms associated with this behavior. Therefore, information about the expression patterns of genes, possibly involved in the foraging behavior for oviposition in this parasitic wasp, constitutes the starting point to explore the underlying molecular mechanisms intervening in this process. This knowledge could contribute to increasing the field efficiency of *D*. *longicaudata* as a biological control agent of fruit fly species in the future.

The present study aimed at identification and first characterization of soluble binding proteins potentially associated with host foraging in *D*. *longicaudata*. The main hypotheses of this study were as follows: 1) during host foraging behavior, the female *of D*. *longicaudata* deploys soluble binding proteins to detect odors associated with the host; 2) the host detection and oviposition performance of *D*. *longicaudata* females can be modified by transcriptional silencing of some of these transcripts.

## Materials and methods

### Insect rearing

Parasitoids and fruit flies used in the study were obtained from the experimental rearing facility at Instituto de Genética “E. A. Favret” (IGEAF-INTA) gv-IABIMO (CONICET). Females of *D*. *longicaudata* used in the experiments were obtained from a colony reared on *A*. *fraterculus* for 25 generations. This colony was established from *D*. *longicaudata* insects reared on *C*. *capitata* for more than 200 generations and established with individuals from Fundación Miguel Lillo-CIRPON [38]. Insects were maintained under controlled rearing conditions [25 ± 2°C, 65 ± 5% relative humidity (R.H.) and a 12:12 (light/dark) photoperiod] following protocols described by [35, 39]. Biosecurity considerations were in agreement with CONICET resolution 1619/2008 and with the WHO Biosecurity Handbook (ISBN 92 4354 6503). *A*. *fraterculus* larvae used for *D*. *longicaudata* rearing and behavioral assays were obtained from the experimental rearing facility at Instituto de Genética “E. A. Favret” (IGEAF, INTA) gv-IABIMO (CONICET).

### Tissue collection, RNA extraction, and cDNA synthesis

Total RNA was extracted from three types of tissue of *D*. *longicaudata* adult individuals of both sexes: whole body (without antenna), antenna, and ovipositor (only females). Samples were stored in 600 μl of Trizol® reagent (Invitrogen Carlsbad, CA, USA) according to the manufacturer’s specifications until RNA isolation. Specifically, we obtained five replicates of each pooled sample, as follows: “female body” included five whole bodies (without antennae) of 7- to 10-day-old naïve females; “male body” included five whole bodies (without antennae) of 7- to 10-day-old mated males;”male antenna” included the antennae of 30 males (7- to 10-day-old mated males), “female antenna” comprised the antennae of 30 females (7- to 10-day-old naïve females), and the “ovipositor” sample was composed of 30 ovipositors of 7- to 10-day-old naïve females. In the case of females, naïve status represents mated adults with no experience in oviposition. This physiological condition was selected to analyze the basal expression of genes potentially associated with host foraging (to reduce the effect of other biological processes on olfaction, such as mating). The integrity of the extracted RNA was tested by using 1% w/v agarose gel electrophoresis visualized by ethidium bromide staining. In addition, the RNA samples were quantified by a ND-1000 spectrophotometer (Thermo Fisher Scientific-NanoDrop®, Wilmington, USA) at optical density (OD) 260 nm (2 μl of a 1:10 dilution).

From the aforementioned RNA samples, cDNA was synthesized by using the Improm-II Kit (Promega®, USA), according to the manufacturer’s specifications and with temperature-cycling parameters, as follows: 70°C, 5 min (1 cycle); 25°C, 5 min (1 cycle); 37°C, 120 min (1 cycle); 4°C, 5 min (1 cycle).

### Identification of putative soluble binding protein transcripts

A local tBLASTn [40] against a *D*. *longicaudata* whole-body transcriptome [41] was performed to identify candidate unigenes that encode soluble binding proteins associated with chemical communication. A database of soluble binding proteins (OBPs, CSPs, and NPC2s) was used as a query and recorded from the following species: *D*. *melanogaster* (http://flybase.org), this database was included due to its high number of identified sequences for soluble binding proteins; *A*. *mellifera* and *N*. *vitripennis* (http://hymenopteragenome.org), as taxonomically related species with genomic information and annotated genes of soluble binding proteins; and *Diachasma alloeum* Muesebeck (Hymenoptera: Braconidae); *Fopius arisanus* Sonan (Hymenoptera: Braconidae); *Microplitis demolitor* Wilkinson (Hymenoptera: Braconidae), and *M*. *mediator* (http://www.insect-genome.com) as the closest related species. Transcript identification and annotation were also analyzed by BLASTn searches on the NCBI databases (https://blast.ncbi.nlm.nih.gov/Blast.cgi?PROGRAM=blastn&PAGE_TYPE=BlastSearch &LINK_LOC=blasthome). The nucleotide sequences of transcripts were translated to protein sequences using the NCBI ORFinder (https://www.ncbi.nlm.nih.gov/orffinder) and checked again (BLASTp) on the database SWISS-PROT (https://www.uniprot.org/blast/). The data analyzed in this study comes from a transcriptome of *D*. *longicaudata* [41] submitted to NCBI as Sequence Read Archive under the accession number SRP072867. The mentioned Transcriptome Shotgun Assembly project has been deposited at GenBank under the accession GELG00000000.

### Transcript sequences and phylogenetic analyses

A phylogenetic analysis was performed using the Bayesian Inference (BI) method and a Conserved Domain Analysis, based on the amino acid sequences (open reading frames) of selected transcripts (S1 Table). The orthologous protein sequences from the genomes and transcriptomes of the following species were used in the analysis: *A*. *mellifera*; *N*. *vitripennis*; *F*. *arisanus*; *D*. *alloeum*, and *M*. *demolitor*. The protein sequence alignments were performed using Clustal Ω (https://www.ebi.ac.uk/Tools/msa/clustalo/) and Pfam database [42] and Bioedit to edit the alignments [43]. The domain analysis was performed using the EMBO-InterPro Scan (https://www.ebi.ac.uk/interpro/) and SignalP-5.0 Server (http://www.cbs.dtu.dk/services/SignalP/). The XML files necessary for the BI analysis were generated by using Beauti v1.8.3 [44]. The amino acid substitution model used in the analyses was LG model G+I (5 categories) for CSPs and WAG model+G+I (5 categories) for OBPs and NPC2-like proteins (determined by Mega v6), and the Yule Speciation Model was used in all analyses [45]. Beast v1.8.4 [44] on CIPRES Science Gateway server [46] was used for the BI analyses. Two independent chains were made under the same conditions until the convergence was achieved, where the effective sample size value was > 200, and determined with Tracer v.1.6.0. Each run produced 100 million trees from a random tree. Both runs were combined using LogCombiner v1.83 discarding the first 10% of each run. The tree with the maximum credibility of the clade was obtained with TreeAnnotator v1.8.3, visualized, and edited with FigTree v1.4.2 [44]. Finally, the outgroup of each sub-clade was defined as those sequences belonging to the evolutionarily most distant taxa: *A*. *mellifera* or *N*. *vitripennis* depending on each sub-clade.

### RT-PCR assay

To evaluate the presence of expression of the selected transcripts (S1 Table) and to perform RT-PCR reactions, cDNA samples from each tissue: whole body (without antennae) and antennae (for males and females), and ovipositor (only for females) were used as template (2 μl of a 1:10 dilution). The quality of the synthesized cDNA was checked by using elongation factor 1-alpha (EF1-α) as a positive control (S1 Table). All primers were designed by using Primer3web (http://bioinfo.ut.ee/ primer3/) and manufactured by Invitrogen® (Carlsbad, CA, USA). Taq DNA polymerase Kit (Taq Pegasus PB-L®) was used for RT-PCR reactions, according to the manufacturer’s specifications, and at the following temperature-cycling parameters: 95°C, 5 min (1 cycle); 94°C, 15 s; 58°C, 15 s; 72°C, 20 s (35 cycles); 72°C, 10 min (1 cycle). PCR amplicons were run in 1% w/v agarose gel electrophoresis and visualized by ethidium bromide staining.

### Quantitative real-time PCR (qPCR)

Selected transcripts identified *in silico* as potential soluble binding proteins (S1 Table) were analyzed by qPCR to characterize their expression levels. The cDNA samples from female body (without antennae) and from antennae of females (five biological replicates for each of the treatments) were used in the analysis.

qPCR reactions were performed in a Light Cycler 96 (Roche®) and each reaction consisted of a total volume of 10 μl, containing 5 μl of Fast Start Essential DNA Green Master (Roche®), 0.2 μl (10 μM) of each forward and reverse primers, 2.6 μl of dH2O, and 2 μl of cDNA template (a 1:10 dilution of a solution of ~500 ng / μl). The cycling parameter was 95°C for 5 min, followed by 40 cycles at 95°C for 10 s and 60°C for 45 s, ending with a melting curve product amplification. All qPCR experiments were performed according to previously defined parameters [41]. EF1-α and β actin were used as internal reference genes (S1 Table), previously used in this species [41]. The stability and suitability of these reference genes were evaluated with BestKeeper, Normfinder, Genorm, and the comparative delta-Ct method software tools [47, 48]. The normalized relative quantity (NRQ) was calculated for each gene of interest (GOI) following the formulas in [49], and statistical analysis was performed over the Log2 (NRQ) according to [50].

PCR amplicons from the analyzed transcripts were purified and sequenced (forward and reverse primer) by an automatic sequencer—capillary electrophoresis (SIGYSA, IABIMO, CONICET-INTA, Buenos Aires, Argentina) to confirm their nucleotide sequence and univocal identity.

### Synthesis of dsRNA and delivery method

The selection of candidate transcripts to be analyzed by RNA interference (RNAi) [51] was based on the phylogenetic analyses performed in the present study and previous reports on taxonomically related species (Hymenoptera: Braconidae) [31, 52, 53]. For the RNAi assay, a coding fragment of each selected gene was amplified by RT-PCR using gene-specific primers (S1 Table). The products obtained were used as a template for the synthesis of dsRNA by using the RNA Pol. T7 Kit (Thermo Fisher Scientific®, MEGAscript™ RNAi Kit). The dsRNA was resuspended and provided to the insects in sterile tap water to avoid the osmotic shock. Blue food dye Erioglaucine (E133, Merck®, Darmstadt, Germany) was used in the water to identify the insects that consumed the dsRNA by assessing the presence of light blue color in their abdomens. We established a dose of 0.005% w/v to observe the color effect in the treated insects, although higher doses are harmless to insects [54]. The insects (naïve females), previously starved for 48 h, had access to a colored water solution of 20 ng / μl dsRNA for 48 h. Each insect ingested ~ 100 ng of dsRNA (~ 5 μl of solution) and fed on honey *ad libitum*, adapted from [55–57]. The control group received the same treatment, but the dsRNA was specific to the enhanced green fluorescent protein gene (eGFP), absent in insect species genomes and previously used as a control dsRNA of RNAi experiments in other braconid wasps [57].

After the behavioral experiment (see below), the decrease in mRNA levels of target transcripts was assessed by qPCR. The RNA and cDNA samples were obtained from females randomly selected for each treatment (2 h after the behavioral assay) using the above-mentioned protocols. Each sample consisted of the whole body of five pooled females. The qPCR was performed following the methods described above.

Joint silencing was performed for OBPs 9, 10, 11, and 12 proteins, given their high similarity at the nucleotide sequence level (S1A Fig) and because individual silencing was not obtained in preliminary assays. However, the level of expression of each of these transcripts was analyzed by qPCR, since this technique has a higher level of accuracy at the sequence level than the RNAi, as previously described by [58]. For this purpose, two different dsRNAs (both inside the coding region: 70–469 pb) were used: ds(OBP9-12)a from 93 to 346 pb and ds(OBP9-12)b from 189 to 468 pb (S1A Fig).

### Foraging behavior and survival assays

The performance of RNAi-silenced females (7–10-day-old naïve females) was evaluated by behavioral assays under laboratory conditions. The experiment was conducted in groups of five *D*. *longicaudata* females (n = 20 groups tested per treatment). The grouping strategy to evaluate the oviposition behavior of females was implemented because the presence of conspecifics increases parasitic activity [59]. The dsRNA was delivered in dyed water for 48 h and replaced with tap water 48 h before the assay. The behavioral assays were carried out 96 h after the application of the dsRNA. The experimental design was adapted from [55, 59] and defined after preliminary assays. For the assay, five females were placed in a small glass container (5.5 cm in diameter, 13 cm high) covered with a 6 cm Petri dish, placed inside a bigger cylindrical glass container (20 cm in diameter and 30 cm in height) closed at the top with a voile cloth, forming the experimental arena. A thin thread connected to the Petri dish made it possible to release the females from outside the arena with minimal disturbance. The females remained in the smallest container for 24 h before the assays for habituation. During the assay, 250 L3 larvae of *A*. *fraterculus* were offered in an oviposition unit (OU) that consisted of a Petri dish wrapped in voile cloth placed upside down [39]. Each treatment was tested for 25 min. Once the RNAi-silenced and control females were released, two variables were recorded: i) the number of females foraging on the OU every one minute; and ii) the latency for the first female to visit the OU. After the assay, the larvae from each replicate were placed in 200 mL jars with vermiculite as pupation substrate and kept under controlled conditions (25 ± 1°C; 65 ± 5% R.H.) to record the number of emerged parasitoids. In all cases, the conditions detailed previously were adapted from [60]. In addition, after the behavioral assays, female survival was recorded (n = 150 females) on RNAi-silenced and control females based on the protocol and variables described by Viscarret *et al*. [39]. We registered the following variables: i) mean longevity of the adult female (lifespan of the i female/initial number of females); ii) mean survival by age (lx), (number of female at age x/initial number of females).

### Statistical analysis

Data analysis was performed using SPSS Statistics 22.0 software (SPSS Inc., Chicago, IL, USA). A two-tailed Student’s t-test [61] was performed on the data for all experiments (qPCR, foraging, and survival assays), and the results are presented as the mean ± SEM. For mean longevity analysis, a Log-rank (Mantel-Cox) test was used [62]. The results were plotted using the Prism GraphPad v6 software (GraphPad Software, Inc. Accessed 10/04/2020; http://www.graphpad.com/faq/viewfaq.cfm?faq=1362).

## Results

### Identification of soluble binding protein transcripts

The bioinformatic analyses of transcripts identified from the *D*. *longicaudata* whole-body transcriptome [41] yielded the following unigenes: seven CSPs, with two possible isoforms for CSP 3 (CSPs 10 and CSP 11, S1B Fig); 14 OBPs; and four NPC2-like proteins. Detailed information on each identified transcript is listed and categorized in Table 1.

**Table 1:** Soluble binding proteins identified in a whole body transcriptome of *D*. *longicaudata*.

** **	***D*. *longicaudata* transcriptome database (best tBLASTn hit)**	** **				**UniprotKB/SwissProt database (best BLASTp hit)**	** **		** **	
**Gene name**	**Accession Number**	**Score**	**E-value**	**ID query**	**Species**	**Gene subject**	**ID sequence**	**Score**	**E-value**	**Ident**
										
**CSP1**	GELG01006173.1	77.8	7E-19	DQ855486	*A*. *mellifera*	PebIII_2 /CSP (*F*. *arisanus*)	A0A0C9RC91	215	2.40E-20	40 %
**CSP2**	GELG01043217.1	93.6	1E-25	NP_726402.1	*D*. *melanogaster*	PebIII_4/CSP (*F*. *arisanus*)	A0A0C9Q9U6	289	5.00E-35	98.0%
**CSP3**	GELG01013729.1	109	7E-31	NP_611990.1	*D*. *melanogaster*	PebIII_9/CSP ( *F*. *arisanus*)	A0A0C9R9K1	283	2.3E-30	48 %
**CSP4‡**	GELG01000290.1	141	2E-40	DQ855484	*A*. *mellifera*	transcriptome assembly error (equal sequence than CSP5)	-	-	-	-
**CSP5**	GELG01000294.1	141	7E-43	DQ855484	*A*. *mellifera*	PebIII_1 /CSP (*F*. *arisanus*)	A0A0C9QFN9	470	2.4E-58	67 %
**CSP6**	GELG01007750.1	142	2E-43	DQ855487	*A*. *mellifera*	PebIII_6/CSP (*F*. *arisanus*)	A0A0C9RRW4	568	2.2E-73	83.3%
**CSP7&**	GELG01001790.1	166	2E-52	NP_611990.1	*D*. *melanogaster*	Chemosensory protein 1 (*Bactrocera dorsalis*)	A0A034W0K7	552	3.8E-71	91.4%
**CSP8**	GELG01048086.1	79.7	2E-19	XP_011307929.1	*F*. *arisanus*	PebIII_3/CSP (*F*. *arisanus*)	A0A0C9R724	429	1.2E-57	95.9%
**CSP9**	GELG01007729.1	196	1E-64	XP_011296666.1	*F*. *arisanus*	A10_0/CSP (*F*. *arisanus*)	A0A0C9RWR5	487	2.3E-61	76.7%
**CSP10Ϯ**	GELG01015394.1	115	2E-33	XP_011305927.1	*F*. *arisanus*	PebIII_9/CSP ( *F*. *arisanus*)	A0A0C9R9K1	274	1.00E-28	43.9%
**CSP11Ϯ**	GELG01010361.1	114	1E-32	XP_011305927.1	*F*. *arisanus*	PebIII_9/CSP ( *F*. *arisanus*)	A0A0C9R9K1	286	1.8E-30	43.3%
**OBP1**	GELG01005963.1	62.4	6E-13	NV10225-PA	*N*. *vitripennis*	Pbprp1_1/OBP (*F*. *arisanus*)	A0A0C9RWQ2	507	1.1E-63	65.2%
**OBP2**	GELG01007924.1	51.6	1E-09	NV12594-PA	*N*. *vitripennis*	OBP1 (*F*. *arisanus*)	A0A0C9QKG6	542	5.4E-68	72.8%
**OBP3**	GELG01007569.1	81.3	5E-20	NV12608-PA	*N*. *vitripennis*	Obp56d_4 (*F*. *arisanus*)	A0A0C9RSX5	439	3.2E-53	63.2%
**OBP4**	GELG01006993.1	117	5E-33	XP_008543434.1	*M*. *demolitor*	Obp19a_0 (*F*. *arisanus*)	A0A0C9QRI4	616	4.6E-80	76.0%
**OBP5**	GELG01007011.1	140	3E-42	XP_008559486.1	*M*. *demolitor*	Odorant binding protein 3 (*Macrocentrus cingulum*)	A0A221I2K4	381	1.6E-44	53.0%
**OBP6**	GELG01005241.1	238	3E-79	XP_011304865.1	*F*. *arisanus*	Pbprp1_2/OBP (*F*. *arisanus*)	A0A0C9RWT1	683	1.9E-90	89.4%
**OBP7**	GELG01007880.1	33.9	0.006	NV15819-PA	*N*. *vitripennis*	SecA_0/OBP (*F*. *arisanus*)	A0A0C9R7J0	254	2.5E-25	35.6%
**OBP8**	GELG01002295.1	139	5E-39	XP_008559486.1	*M*. *demolitor*	Odorant binding protein 10 (*M*. *mediator*)	G3M0D3	354	2.1E-40	45.0%
**OBP9**	GELG01001415.1	123	4E-35	XP_011311566.1	*F*. *arisanus*	Obp56a (*F*. *arisanus*)	A0A0C9PK74	319	2.8E-35	43.5%
**OBP10**	GELG01001518.1	157	1E-47	XP_011311566.1	*F*. *arisanus*	Obp56a (*F*. *arisanus*)	A0A0C9PK74	410	6.7E-49	64.3%
**OBP11**	GELG01003729.1	157	7E-47	XP_011311566.1	*F*. *arisanus*	Obp56a (*F*. *arisanus*)	A0A0C9PK74	396	5.5E-47	51.2%
**OBP12**	GELG01001519.1	175	4E-55	XP_011311566.1	*F*. *arisanus*	Obp56a (*F*. *arisanus*)	A0A0C9PK74	452	1.7E-55	62.8%
**OBP13&**	GELG01005757.1	119	2E-33	Obp99a-PA	*D*. *melanogaster*	General odorant binding protein 99a (*C*. *capitata*)	W8ASY6	747	3.6E-100	100.0%
**OBP14**	GELG01031851.1/GELG01045208.1	88.6	2E-37	GB50936-PA	*A*. *mellifera*	OBP10 (*A*. *mellifera*)	Q1W644	529	5.1E-67	80.9%
**OBP15&**	GELG01008185.1	144	2E-44	Obp99a-PB	*D*. *melanogaster*	General odorant binding protein 99a (*C*. *capitata*)	W8CAL3	802	3.6E-108	100.0%
**OBP16&**	GELG01008020.1	87.8	2E-22	Obp99a-PA	*D*. *melanogaster*	General odorant binding protein 99a (*C*. *capitata*)	W8CD84	777	1.6E-104	100.0%
**OBP17**	GELG01007052.1	67.8	5E-15	NV12608-PA	*N*. *vitripennis*	Pbprp1_0/OBP (*F*. *arisanus*)	A0A0C9PWB3	463	4.2E-57	65.9%
**NPC2a**	GELG01007456.1	253	6E-83	XP_015119973.1	*D*. *alloeum*	Protein NPC2 homolog (*Drosophila guanche*)	A0A3B0JF70	377	5E-44	50.3%
**NPC2b**	GELG01006291.1	255	2E-84	XP_015120673.1	*D*. *alloeum*	Epididymal secretory protein E1 (*Ooceraea biroi*)	A0A026W6F8	438	4E-48	55.2%
**MD-2**	GELG01005505.1	249	9E-82	XP_015114239.1	*D*. *alloeum*	Md-2-related lipid-recognition (*Lasius niger*)	A0A0J7L5K5	479	2E-59	53.5%
**ESR16**	GELG01007261.1	248	1E-80	XP_015109258.1	*D*. *alloeum*	Ecdysteroid-regulated 16 kDa protein (*Camponotus floridanus*)	E2AC22	384	7E-45	48.1%

The genes highlighted in gray were excluded from subsequent analysis because they were assembly errors (‡), contamination (&) or possible isoforms (Ϯ).

### Sequence and phylogenetic analysis

**Fig 1:**
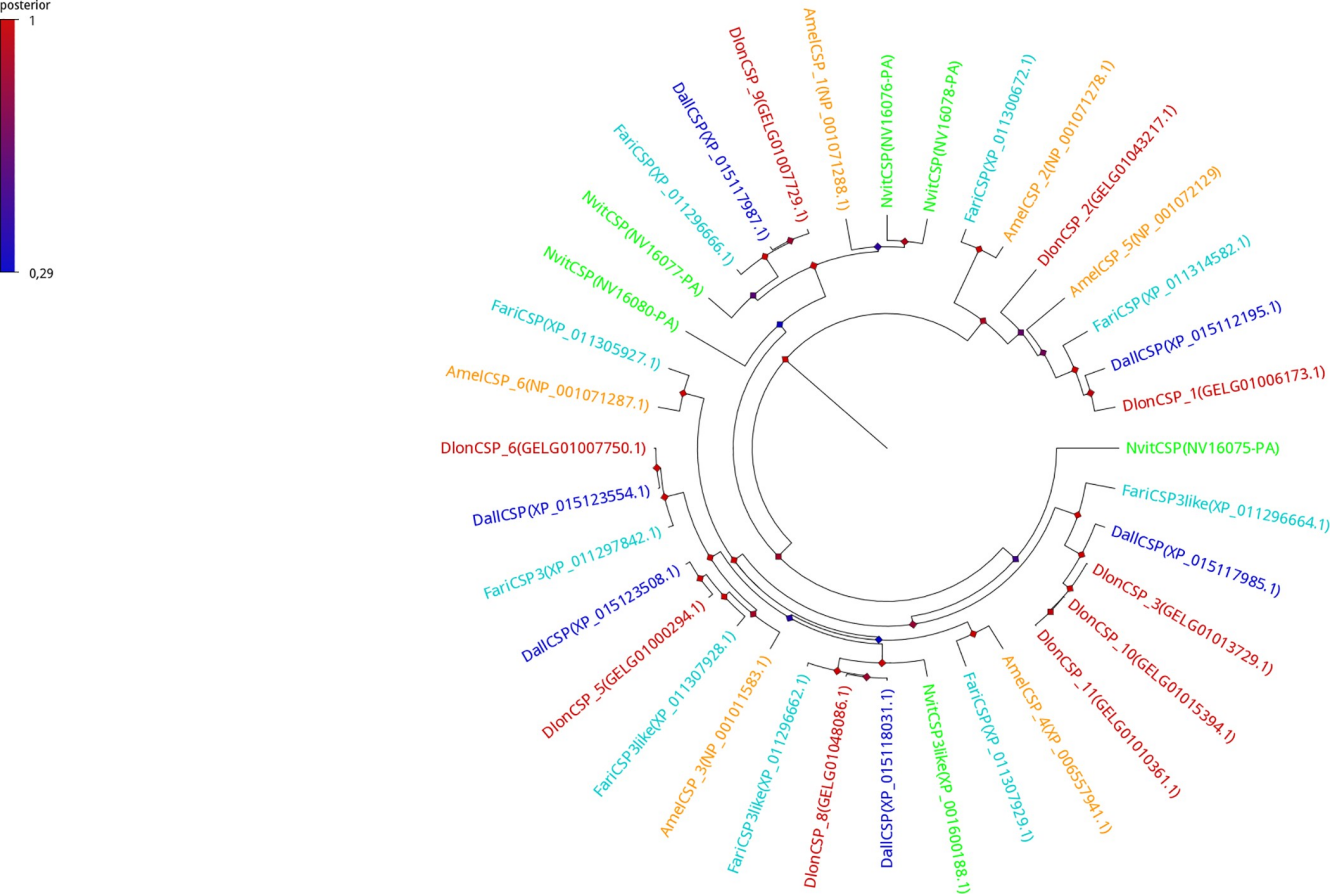
Phylogenetic tree (Bayesian Inference) of CSP protein sequences from different hymenopteran insects. *A*. *mellifera* (Amel, yellow); *N*. *vitripennis* (Nvit, green); *F*. *arisanus* (Fari, light blue); *D*. *alloeum* (Dall, blue); and *D*. *longicaudata* (Dlon, red). Node color indicates posterior values, while red color represents a high credibility node (scale top left).

**Fig 2:**
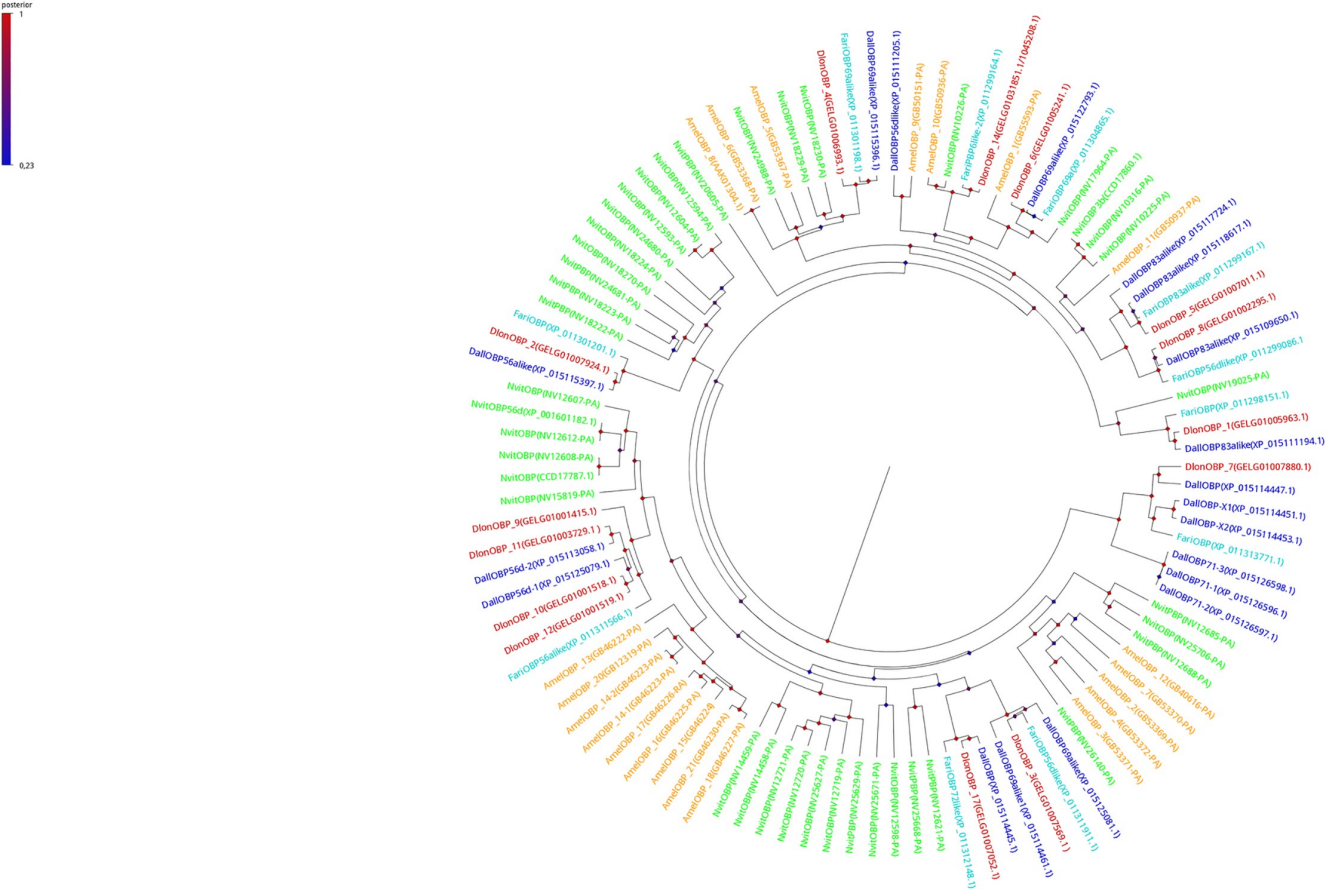
Phylogenetic tree (Bayesian Inference) of OBP protein sequences from different hymenopteran insects. *A*. *mellifera* (Amel, yellow); *N*. *vitripennis* (Nvit, green); *F*. *arisanus* (Fari, light blue); *D*. *alloeum* (Dall, blue); and *D*. *longicaudata* (Dlon, red). Node color indicates posterior values, while red color represents a high credibility node (scale top left).

**Fig 3:**
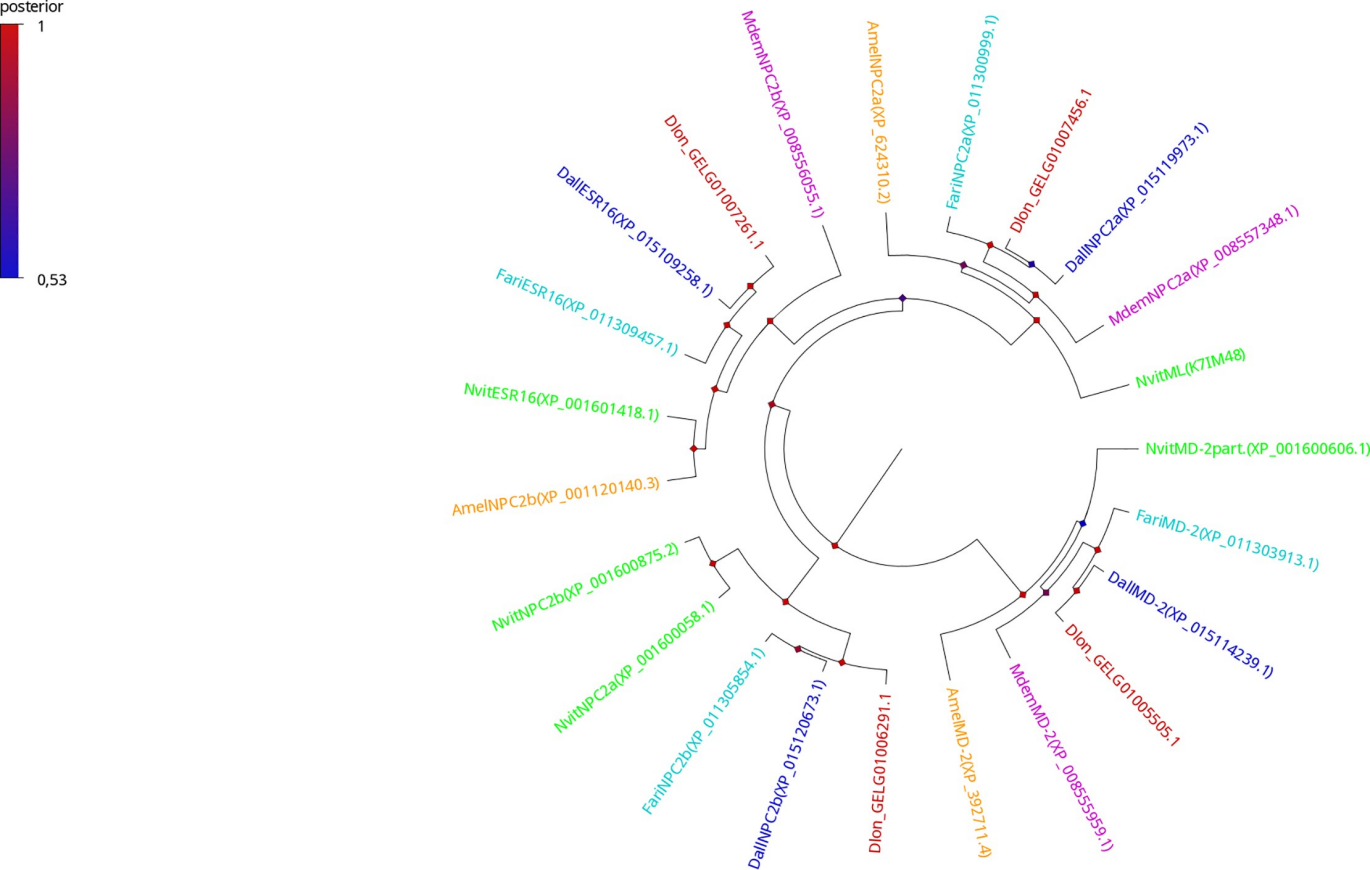
Phylogenetic tree (Bayesian Inference) of NPC2-like protein sequences from different hymenopteran insects. *A*. *mellifera* (Amel, yellow); *N*. *vitripennis* (Nvit, green); *F*. *arisanus* (Fari, light blue); *D*. *alloeum* (Dall, blue); *M*. *demolitor* (Mdem, violet) and *D*. *longicaudata* (Dlon, red). Node color indicates posterior values, while red color represents a high credibility node (scale top left).

Phylogenetic trees for CSP, OBP, and NPC2-like proteins (Figs 1–3 respectively) showed that each protein sequence of *D*. *longicaudata* was clustered with the proteins of other closely related hymenopteran species. The phylogenetic analyses revealed that all classes of soluble binding proteins (CSP, OBP, and NPC2-like) analyzed here were significantly different from each other within *D*. *longicaudata* (posterior values > 0.5, BI analysis). In contrast, homologous soluble binding proteins among species shared a highly similar amino acid sequence and were associated within the same clade with high posterior values support (Figs 1–3).

Amino acid sequence analysis of the soluble binding protein identified in *D*. *longicaudata* showed a percentage of identity ranging from 19% to 54% (30% on average) in the case of CSPs (S2A Fig). OBP protein sequences shared a percentage of identity between 15% and 90% (23% on average) (S2B Fig), and NPC2-like proteins shared between 23% and 32% (26% on average) of their amino acid sequences (S2C Fig). Regarding the number of conserved cysteines in the sequences, all members of the CSPs family possess the four typical cysteines, characteristic of this group. For OBPs members, only the Classic OBP motif (six conserved cysteines) was found. In several cases (OBPs 3, 5, 6, 8, 9, 10, 11, 12, and 14), an additional conserved cysteine was observed, but inside the signal peptide (~ 1–21 aa) and not as part of an α-helix. All the identified NPC2-like proteins had seven conserved cysteines in their amino acid sequences.

### Expression patterns of soluble binding protein transcripts

RT-PCR results showed that all soluble binding protein transcripts (CSP, OBP, and NPC2-like) were detected in all tissues analyzed, antennae and the whole body (without antennae) (both sexes) and ovipositor (only females) (S3–S12 Figs). Sequencing by capillary electrophoresis was used to confirm the identity of the transcripts analyzed by RT-PCR and qPCR (S13 Fig). qPCR analysis was performed only for females, because the main objective of the present work, is the identification of soluble binding proteins potentially associated to the detection of the host for oviposition.

By means of RT-PCR and sequencing, we identified assembly errors (described as different transcript IDs from the *D*. *longicaudata* transcriptome matched with the same query sequence or a transcript ID with more than one query sequence) (Table 1). We also detected host contamination, as *Ceratitis capitata* sequences were *in silico* identified for CSP 4 and 7, and OBPs 13, 15, and 16 (Table 1). This type of contamination was confirmed by the absence of PCR amplification in all *D*. *longicaudata* tissues analyzed (S3–S12 Figs) and *D*. *longicaudata* genomic DNA. Multiple alignments were performed to compare sequenced PCR amplicons with the transcript assembled sequences to confirm their nucleotide sequence and univocal identity (S13 Fig).

**Fig 4:**
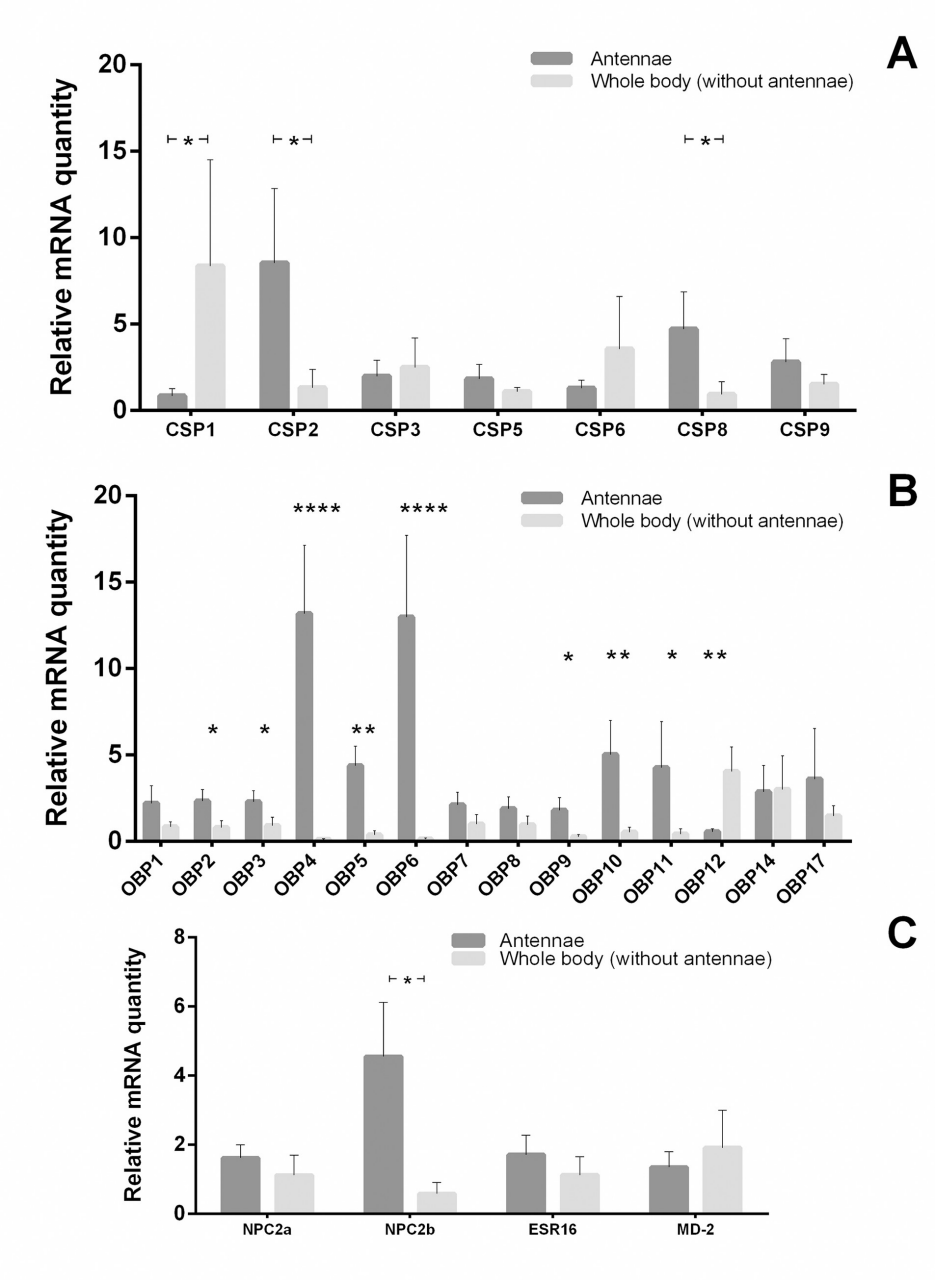
Expression levels of CSPs (A), OBPs (B), and NPC2-like (C) in *D*. *longicaudata* female tissues by qPCR. The relative expression level (NRQ) is indicated as mean ± SEM. The tissues analyzed were as follows: female whole body (5 female bodies without antennae) and female antenna (the antennae of 30 females per sample). The asterisk (*) represents significant differences (n = 5 samples; * p < 0.05, ** p < 0.01, **** p < 0.0001, two-tailed *t-test*) among the tissues.

The qPCR analysis performed on the female antenna and body (without antennae) showed that three CSPs were differentially expressed between these tissues. Specifically, CSP 2 and CSP 8 were significantly more highly expressed in female antenna than in the body (p = 0.036 and p = 0.044, respectively). Conversely, CSP 1 was significantly more highly expressed in the body (without antennae) in comparison to antenna (p = 0.040) (Fig 4A). In addition, nine OBPs were differentially expressed between these tissues. OBP 2 (p = 0.039), OBP 3 (p = 0.050), OBP 4 (p = 0.00007), OBP 5 (p = 0.002), OBP 6 (p = 0.00009), OBP 9 (p = 0.011), OBP 10 (p = 0.011), and OBP 11 (p = 0.043) showed an over-expression in female antenna in comparison to the whole body (without antennae). The opposite pattern was detected for OBP 12 (p = 0.010) (Fig 4B). Regarding NPC2-like transcripts, NPC2b was significantly highly expressed in the female antenna relative to the whole body (without antennae) (p = 0.025) (Fig 4C).

### RNAi assays

**Fig 5:**
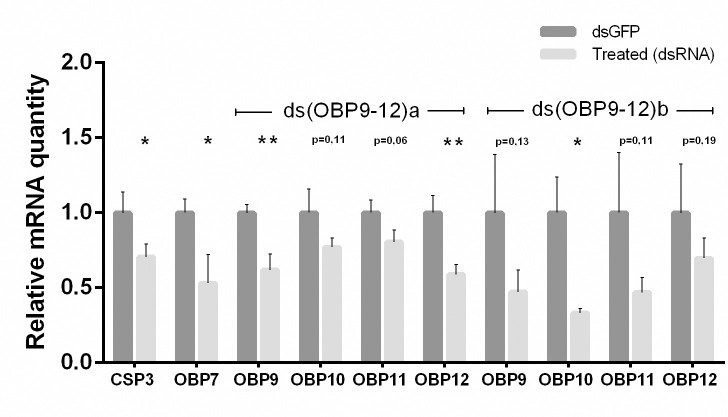
Transcript expression levels in response to dsRNA treatment measured by qPCR. The normalized relative quantity (NRQ), represented as fold change relative to controls, is indicated as mean ± SEM (n = 5 female whole-body per sample). The asterisk (*) represents significant differences (n = 5; * p < 0.05 two-tailed *t-test*) among target gene-specific dsRNA and control dsRNA (eGFP).

CSP 3 and OBPs 7, 9, 10, 11, and 12 were selected for the RNAi analysis based on the phylogenetic analyses performed in the present study and previous reports on taxonomically related species. The RNAi effect (the expected decrease in the transcription of silenced genes), evaluated by qPCR of RNAi-silenced females, showed that CSP 3 (p = 0.046) and OBP 7 (p = 0.035) were significantly silenced in comparison to the control groups (Fig 5). Using the RNA interference ds(OBP9-12)a, OBPs 9 and 12 were significantly silenced (n = 5; p = 0.01 and p = 0.004, respectively). Although OBPs 10 and 11 were not significantly affected after the RNAi, their expression levels decreased (p = 0.111 and p = 0.058, respectively) (Fig 5). Conversely, using ds(OBP9-12)b, only OBP 10 was significantly silenced (p = 0.016), and OBP 9, 11, and 12 decreased their expression levels by 50, 80, and 30%, respectively, although they were not significantly different from the control treatment (p = 0.125, p = 0.110, and p = 0.191, respectively) (Fig 5).

### Behavioral assays on RNAi-silenced females

**Fig 6:**
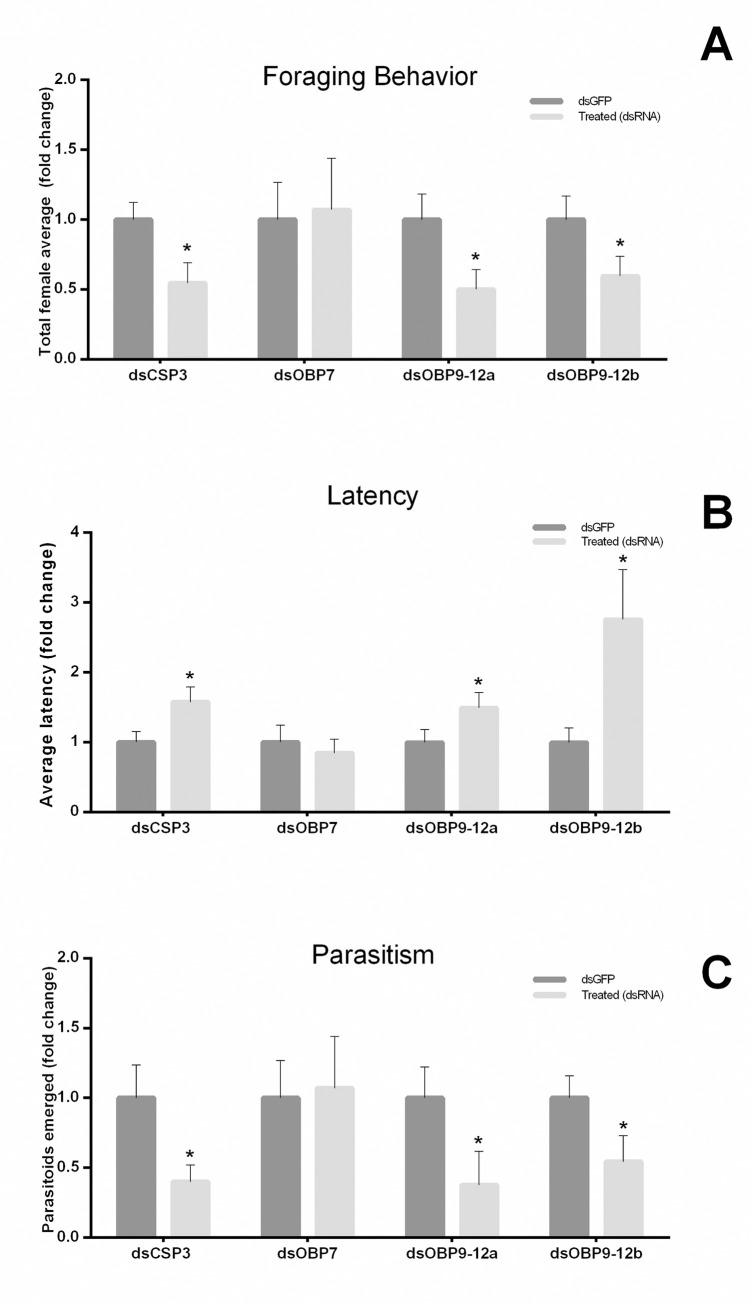
*In vivo* performance of females (previously silenced by CSP 3, OBPs 7, 9, 10, 11, and 12) by behavioral assays: (A) Foraging behavior; (B) Latency time and (C) Parasitism rate. The asterisk (*) represents significant differences (n = 14 all treatments and n = 7 for OBP7; * p < 0.05 two-tailed *t*-test) among target-gene-specific dsRNA and control dsRNA (eGFP).

Foraging behavior and survival of adult females were evaluated for silenced and non-silenced (control) adult females. For dsCSP 3 silenced females, the number of females foraging on the OUs, the latency for the first female to visit the OU, and the number of emerged parasitoids were significantly affected in comparison to the control treatments (dsGFP). The foraging behavior and the number of emerged parasitoids (from fly larvae used in the assays) decreased their values in the case of silenced females (p = 0.012 and p = 0.015, respectively). Conversely, the latency had increased values in this group (p = 0.019) (Fig 6A–6C). Silencing of OBPs 9–12 (using two different dsRNAs) was also associated with females’ foraging performance. RNA interference ds(OBP9-12)a decreased the foraging behavior and the number of emerged parasitoids (p = 0.020 and p = 0.035, respectively), but the latency increased (p = 0.049). Using ds(OBP9-12)b showed a decrease in foraging behavior and emerged parasitoids (p = 0.046 and p = 0.044, respectively), but an increased latency (p = 0.018). The treatment of the OBP 7 showed no significant difference for the three recorded variables (number of females foraging on the OU, the latency for the first female to visit the OU, and the number of emerged parasitoids) (p = 0.298, p = 0.318 and p = 0.439, respectively).

**Fig 7:**
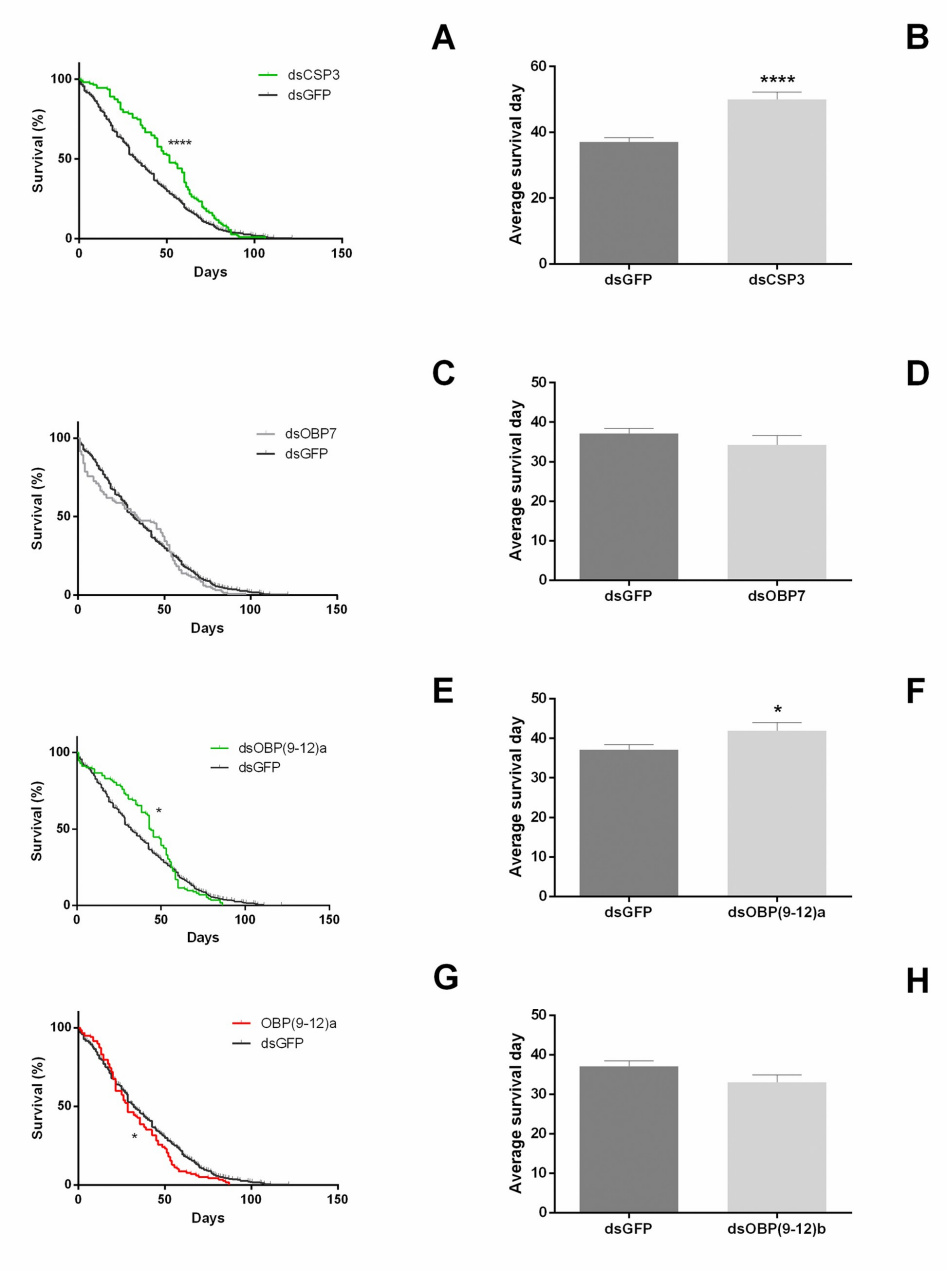
*In vivo* performance of females (previously silenced by CSP 3, OBPs 7, 9, 10, 11, and 12) by behavioral trials: survival analysis measured as mean longevity of the adult females (A, C, E, and G) and survival analysis measured as mean survival by age (B, D, F, and H). The asterisk (*) represents significant differences among target gene-specific dsRNA and control dsRNA (eGFP), for survival analysis (n = 150; * p < 0.05 and **** p < 0.0001 two-tailed *t*-test) and mean longevity analysis (n = 150; * p < 0.05 and **** p < 0.0001 Mantel-Cox).

After the behavioral assays, the survival of RNAi-silenced females was recorded. CSP 3-silenced females showed an increased mean longevity and mean survival by age (p < 0.0001 and p < 0.0001, respectively) (Fig 7A and 7B), as did females treated with ds(OBP9-12)a (p = 0.020 and p = 0.016, respectively) (Fig 7E and 7F). The treatment with ds(OBP9-12)b triggered a decrease in survival by age only in OBP 10-silenced females (p = 0.034), but there were no changes in the mean longevity (p = 0.313) (Fig 7G and 7H). OBP 7 showed no significant difference in mean longevity and mean survival by age (p = 0.229 and p = 0.286, respectively) (Fig 7C and 7D).

## Discussion

This study reports the identification, a first sequence characterization, and the behavioral evaluation of soluble binding proteins in *D*. *longicaudata*. The number of sequences identified here were similar to that of other hymenopteran species: *A*. *mellifera*, six CSPs, 21 OBPs and two NPC2-like [19, 63]; *N*. *vitripennis*, nine CSPs, 90 OBPs and four NPC2-like [64]; *M*. *mediator*, three CSPs, 20 OBPs and two NPC2-like [33, 65]; *F*. *arisanus*, nine CSPs, 11 OBPs and four NPC2-like [66] and *D*. *alloeum*, nine CSPs, 15 OBPs and four NPC2-like [67]. However, the identified soluble binding proteins do not match the number of transcripts identified in a transcriptome of pooled tissues (antenna, head, thorax, abdomen, and legs) of *D*. *longicaudata*, which reported 69 CSPs and 43 OBPs [68]. The reason for such differences might be related to the fact that we based our screening on a transcriptome obtained from the whole body of the wasp, which might have reduced the representation of genes expressed mainly in structures with a high content of cuticle, such as antenna. In fact, only classic OBPs were identified in this work, whereas OBPs like ABPXs, CRLBP, and D7 (which are mainly expressed in olfactory and gustatory structures such as antenna and insect mouthparts) [69, 70] were not detected.

Gene expression profile analyzed by RT-PCR on male and female tissues (antennae, rest of the body, and ovipositor) showed similarities for all soluble binding proteins tested on all the tissues analyzed. However, qPCR analysis on the female antenna and the rest of the body showed that three CSPs, nine OBPs, and one NPC2-like transcripts were differentially expressed between these tissues. Transcripts with a high expression in the female antenna (CSPs 2 and 8, OBPs 2, 3, 4, 5, 6, 9, 10, and 11, and NPC2b) would be potentially related to physiological processes such as host-seeking, feeding, and mating. On the other hand, those transcripts highly expressed in the body (CSP 1 and OBP 12) are potentially related to other physiological processes such as lipid homeostasis and reproduction [1, 4, 5]. Concerning NPC2-like proteins, this particular sub-family (with 7 cysteines conserved in the protein sequence) was found to be mostly associated with structures with chemosensory functions in other insects [4, 17].

The results of the behavioral and qPCR analysis support the hypothesis that OBPs 9–11 are involved in female foraging behavior. Besides, behavioral and qPCR assays support the hypothesis that OBP 12 is involved in physiological processes, directly or indirectly related to longevity. In addition, the hypothesis that CSP 3 and OBP 12 are related to foraging behavior was only supported by the behavioral assays. Regarding CSP 3, its orthologous sequence in *M*. *mediator* (MmedCSP1) showed high expression in adult antennae [71]. Moreover, MmedCSP3 is expressed exclusively in a type 2 basiconica sensillum [71], and the neurons associated with this sensillum have shown high responses to volatile plant compounds [72]. About OBPs 9–12, its orthologous sequences of the parasitoid wasp *Chouioia cunea* (CcunOBP2, 9, and 10) are highly expressed in adult antennae and head [73]. In another wasp species, *Sclerodermus* sp, its orthologous sequences (SspOBP4, 5, and 6) are mostly expressed in female antennae [53]. Moreover, it is interesting that an orthologous sequence of this cluster of proteins in *Phormia regina* (PregOBP56a) is believed to solubilize fatty acids during feeding, and subsequently, it helps to deliver the fatty acids to the midgut [22].

Our results suggest that CSP 3 and OBPs 9–11, but more likely OBP 12, are potentially associated with the regulation of mean longevity of the adult females. This could indicate that the transcriptional gene silencing of these soluble binding proteins (associated with an increase in longevity) could be related to energy-saving pathways, as protein translation process represents one of the most expensive stages in energetic terms, in comparison to other physiological processes [74, 75]. Furthermore, these proteins were found at very high concentrations (between 10–20 mM) in different tissues of other insect species [6]. However, silencing chemosensory-related transcripts do not always affect longevity. Such is the case of RproOBP27 in *Rhodnius prolixus* [76] or OBP 7 in this study, which could indicate that this phenomenon is specific to certain soluble binding proteins. One possible explanation is that some of these proteins act as lipid carriers in some insect species [22].

Several studies in *D*. *melanogaster* and *Caenorabditis elegans* indicate that there is a delicate balance between longevity and lipid homeostasis [*e*.*g*. 77]. Furthermore, this balance could be affected by different biological processes, such as an increase in lipid transport and lipase activity in the midgut, acting on lipid homeostasis and increasing the average longevity of individuals [78]. Differences observed in the mean longevity of adult females between ds(OBP9-12)a and ds(OBP9-12)b can be associated with similar processes (transport of fatty acids), carried out by the same or different genes (*e*.*g*. OBP 12), but in different tissues. We take as reference *D*. *melanogaster* and *C*. *elegans*, where an increase in the lipid concentration in different tissues, such as fat body and midgut, has an antagonistic effect on longevity [77–80]. A second hypothesis could be that this phenomenon is associated, on the one hand, with an off-target effect, related to energy saving for certain proteins, and on the other, to a specific effect only for OBP 12, the only protein of this group that showed a high expression in the rest of the body. To confirm these hypotheses, gene expression should be evaluated in different tissues and periods and under different starvation conditions.

The results presented in this work are in agreement with previous studies, which indicate that soluble binding proteins are involved in different physiological processes and behaviors, such as host-seeking and mean longevity [4, 81]. There is an increasing interest in the development of sustainable and innovative strategies to control insect pest species using the information generated of chemosensory genes [5, 28]. However, soluble binding proteins (mainly for OBPs) are encoded by genes subjected to rapid evolution rates [82–84], and this should be considered in studies of these genes as potential targets associated with biological control. Moreover, this rapid evolution, in some species, results in clade expansions (mainly for OBPs), facilitating the development of species-specific strategies [85, 86].

The study of soluble binding proteins also evidenced the complexity of the underlying molecular mechanisms, since several roles have been described for their orthologous sequences, either in the same or different species, such as recognition and solubilization of ligands; concentration of chemical elements in the sensillum lymph and/or ligand protection; removal or deactivation of odorants after receptor stimulation and ligand desorption [87–91]. Furthermore, recent studies in *D*. *melanogaster* have demonstrated that ORs can be activated (or even increase the neuronal response) in the absence of OBPs [92, 93], which could indicate that these proteins act more as modulators of specific physiological pathways rather than as indispensable components of such processes [94].

In summary, the present study is a first step in the characterization of the molecular mechanisms associated with the detection of cues related to foraging behavior for oviposition in *D*. *longicaudata*. The identification of receptors and other molecules involved in chemosensing and the associated volatile compounds may contribute to improving our knowledge of underlying biochemical pathways of this complex trait. In addition, this information will support future developments of attractant baits to monitor the parasitoid in the field and, therefore, improve the efficiency of the biological control strategy against fruit fly species.

## Supporting information

S1 Fig Multiple nucleotide sequence alignment of (A) *D*. *longicaudata* OBP 9 to 12 nucleotide sequence alignment. Both start and stop codons are highlighted in red (~45/70-470). (B) *D*. *longicaudata* CSP 3, 10, and 11 nucleotide sequence alignment. Both start and stop codons are highlighted in red (~40–410). Alignments were performed by Clustal Ω alignment.
(PDF)

S2 Fig Multiple protein sequence alignment of soluble binding proteins from *D*. *longicaudata* and closely related species: (A) CSPs, the four conserved cysteine residues are marked in black. (B) OBPs, the six conserved cysteine residues are marked in black. (C) NPC2-like proteins, the seven conserved cysteine residues are marked in black. Alignments were performed by Clustal Ω, only the closer orthologous sequence to the *D*. *longicaudata* sequences (according to phylogenetic analysis) were used to improve the visualization of conserved regions. Identical and positive amino acids are highlighted in dark gray and light gray, respectively. Predicted signal peptides are highlighted in red. *A*. *mellifera* (Amel); *N*. *vitripennis* (Nvit); *F*. *arisanus* (Fari); *D*. *alloeum* (Dall); *M*. *demolitor* (Mdem) and *D*. *longicaudata* (Dlon).
(PDF)

S3 Fig RT-PCR results of CSPs and NPC2-like proteins in female antennae. From left to right: molecular-weight size marker, CSPs 1–9, NPC2a, NPC2b, ESR16 and MD-2.
(TIF)

S4 Fig RT-PCR results of OBPs in female antenna. From left to right: molecular-weight size marker, OBPs 1–17.
(TIF)

S5 Fig RT-PCR results of CSPs and NPC2-like proteins in female body (without antennae). From left to right: molecular-weight size marker, CSPs 1–9 and NPC2a, NPC2b, ESR16, and MD-2.
(TIF)

S6 Fig RT-PCR results of OBPs in female body (without antennae). From left to right: molecular-weight size marker, OBPs 1–17.
(TIF)

S7 Fig RT-PCR results of CSPs and NPC2-like proteins in male antenna. From left to right: molecular-weight size marker, CPSs 1–9 and NPC2a, NPC2b, ESR16 and MD-2.
(TIF)

S8 Fig RT-PCR results of OBPs in male antenna. From left to right: molecular-weight size marker, OBPs 1–17.
(TIF)

S9 Fig RT-PCR results of CSPs and NPC2-like in male body (without antennae). From left to right: molecular-weight size marker, CSPs 1–9 and NPC2a, NPC2b, ESR16 and MD-2.
(TIF)

S10 Fig RT-PCR results of OBPs male body (without antennae). From left to right: molecular-weight size marker, OBPs_ from left to right MW, OBPs 1–17.
(TIF)

S11 Fig RT-PCR results of CSPs and NPC2-like in ovipositor. From left to right: molecular-weight size marker, CSPs 1–9 and NPC2a, NPC2b, ESR16 and MD-2.
(TIF)

S12 Fig RT-PCR results of OBPs in ovipositor. From left to right: molecular-weight size marker, OBPs 1–17.
(TIF)

S13 Fig Nucleotide sequence alignments of sequenced PCR amplicons (forward and reverse sequences) and assembled transcripts (obtained from the *D*. *longicaudata* transcriptome) for the selected genes. (PDF)

S14 Fig Potential contamination tested on genomic DNA of *D*. *longicaudata*. From left to right: molecular-weight size marker, negative control, β Actin as a positive control, CSPs 4 and 7, and OBPs 13, 15, and 16.
(TIF)

S1 Table Sequence of primers used for RT-PCR, qPCR, and RNA interference. (XLS)
