# Supplementary material for: Identification and characterization of soluble binding proteins associated with host foraging in the parasitoid wasp Diachasmimorpha longicaudata
Source: PLoS One. 2021 Jun 17;16(6):e0252765. doi: 10.1371/journal.pone.0252765 (PMC8211293; doi:10.1371/journal.pone.0252765)
Supplement: S1 Fig — Multiple nucleotide sequence alignment of (A) D. longicaudata OBP 9 to 12 nucleotide sequence alignment. Both start and stop codons are highlighted in red (~45/70-470). (B) D. longicaudata CSP 3, 10, and 11 nucleotide sequence alignment. Both start and stop codons are highlighted in red (~40–410). Alignments were performed by Clustal Ω alignment. (PDF) [file pone.0252765.s001.pdf]

A

```
                  10        20        30        40        50        60        70        80        90       100
             ....|....|....|....|....|....|....|....|....|....|....|....|....|....|....|....|....|....|....|....|
DlonOBP9     CGCATTTGCAACTGGTTCTAACTTTC-------------GGGAGCTCAGAATAATCCA--ACAACTCATCATGAAATTCCTGGTGATTGCCGTCCTCGTC
DlonOBP11    ---------------------------------------GGAGTCTATAACGATTGAAAAAACAA-TAT---GAAATTCCTCGTCGTTGTGATTTTCGTC
DlonOBP10    ATCAATATCAGCGTGAGATTTCTTTCCCTAATTAAGCCGATCGAATGTTACTCTTCCAAGAACTGGCACAGACTGTCTTAACATTATTTCAATAATTTTT
DlonOBP12    ---------------CACTTTTGCAACTTGCTTTATCCTCCGAAAGCTATACAATTTAAAAAGTCAACATGAAAGTATTT--GTGGTTATCCTCTCCACC

                 110       120       130       140       150       160       170       180       190       200
             ....|....|....|....|....|....|....|....|....|....|....|....|....|....|....|....|....|....|....|....|
DlonOBP9     TGCATCGTTGGTGCTCTGGCATCCGAGCAGAGAGAAACCCCGGAGGCGAGACGGCAGCGATACAGAGATGCTTGCCGTGCGGAGACAGGGGCTGATCAAG
DlonOBP11    TGTCTCGCTGGTGCTCTGGCCCAGGAGTTAACCGAAGCCCAAAGACGGAGACTTAGAGAGCATAGAGATATTTGTATAAGAGAGACAGATGCTAACAGAG
DlonOBP10    ATTCATTTACACGTGAAGGCCCAAGAATTGACGGAAGCGCAAAAGCAGAAAGTCAGGGAGAATAGAGACGCTTGCATTACCGAGACAGGTGCTGACAGAG
DlonOBP12    TGTCTCATTGGTGCTCTGGCCCAAGAATTGACGGAAGCGCAAAAGCAGAAAGTCAGGGAGAATAGAGACGCTTGCATTACCGAGACAGGTGCTGACAGAG

                 210       220       230       240       250       260       270       280       290       300
             ....|....|....|....|....|....|....|....|....|....|....|....|....|....|....|....|....|....|....|....|
DlonOBP9     CTGACATTGATAAGGCTCGCAAAGGCGAATGGGCTGATAAAGAGAACCTTCGTTGTTACACCCTTTGTATGCTGAAGAAAGGGAATCTGATGAATGATGA
DlonOBP11    CCGAGGTCGACAGGGCTCGTCAGGGCCAATGGGCTGATAACCCTCAAATCCGTTGTTTCGCTTTGTGCATGATGAGGCGGTTACGTTTGATGAGTGAAGA
DlonOBP10    AGGAGGTTAATAAGGCTAATAAGGGCGAATGGGCTGACAATGCAAAGATCCGTTGTTTCACCCTCTGTATGCTGAAGAAATGGGGCATGATGAATGATGC
DlonOBP12    AGGAGGTTAATAAGGCTAATAAGGGCGAATGGGCTGACAATGCAAAGATCCGTTGTTTCACCCTCTGTATGCTGAAGAAATGGGGCATGATGAATGATGC

                 310       320       330       340       350       360       370       380       390       400
             ....|....|....|....|....|....|....|....|....|....|....|....|....|....|....|....|....|....|....|....|
DlonOBP9     AGGTGTGCTCGATGAAATCGTCACACGCGACACGTTGGCCCGTAAATTGCCCGCTGAGCAGATCAATGATATCATGGCCAAGTGCAAAGATCTGAAGGGT
DlonOBP11    TGGTCAACTGAATGAAGCTGCGGCTCGGCAGAGATTGGCGTTGGTAGTTCCGCGTGAAAGGGTAGAAGAGATCATGACGAAGTGCAAGGATCTGAAGGGC
DlonOBP10    CGGTGTATTAGATGAAGCTGCAGCTCGCCAGAAGATGGGCTTGCAGATGAAGCCCGAGAAGGTCGAGGAGATCATGACCAAGTGCAAATATCTGAAGGGC
DlonOBP12    CGGTGTATTAGATGAAGCTGCAGCTCGCCAGAAGATGGGCTTGCAGATGAAGCCCGAGAAGGTCGAGGAGATCATGACCAAGTGCAAATATCTGAAGGGC

                 410       420       430       440       450       460       470       480       490       500
             ....|....|....|....|....|....|....|....|....|....|....|....|....|....|....|....|....|....|....|....|
DlonOBP9     TCCAACGCCTGTGGCACTGCGCAAAGGATAATGAAGTGTTATAATGAGCAGAAAACACACCTCATGTAAAAAATAATCCTGTGTTACTACAATGATGATC
DlonOBP11    AACACTCCCTGTGATACTGGCTATTTGGTCCTGAAGTGCTATACTGATAACAGAGCAAACGTCGTCTAATACTAATTCGC-AATCGCTGCTCTATTAATA
DlonOBP10    GATACCGCTTGTGATACAGCATACATGATGATGAAGTGCTACACTGACAACAGAGCAGTCACCGTCTAAGATTAACCCGA-AATTTCTACTATACAAACA
DlonOBP12    GATACCGCTTGTGATACAGCATACATGATGATGAAGTGCTACACTGACAACAGAGCAGTCACCGTCTAAGATTAACCCGA-AATTTCTACTATACAAACA

                 510       520       530       540       550       560       570       580       590       600
             ....|....|....|....|....|....|....|....|....|....|....|....|....|....|....|....|....|....|....|....|
DlonOBP9     CCTATGATTTTGTTGATTATCGGCCAATGCATATGAACTGCTTTTGTGACTAAATTCCCACCATGTTTCGAAACATTATTTATCGGGGCTT-------TA
DlonOBP11    ATTGATAATTGGAATATCACCAGCGAT-GGGGATGATGTAGCGTAGGTTCTGCCATTTTTTCAAGCT---GAGCAGCATTTATTTCGCTTAAAGTATTCG
DlonOBP10    TCGATGATTTTACTGATTTTTTTTGAGTGCACATGATTTAATC--TGTTCAATAAAGTTTTGGATCT---C-TCAGTTTTCCATTGGATTCTTCAATTCA
DlonOBP12    TCGATGATTTTACTGATTTTTTTTGAGTGCACATGATTTAATC--TGTTCAATAAAGTTTTGGATCT---C-TCAGTTTTCCATTGGATTCTTCAATTCA

                 610       620       630       640       650       660       670       680       690       700
             ....|....|....|....|....|....|....|....|....|....|....|....|....|....|....|....|....|....|....|....|
DlonOBP9     TCGTACGATTTGATTTATTGGAATAAAAAGCCATTAAATAAACAAGCATTAATAAATTGAAATGAGAGAACGGCAGTTTTGTGAGTCTCC----------
DlonOBP11    CCTACAGGGAACAATATACAGCGAAGATTTTGGGCACATATTA----CTAAAAAAATATATAAAATATATCTTCCATATGTTATCTATATAGCCTATATA
DlonOBP10    TTGAAATACAACATTTATTGGCGATGATTGAAATTTTCTTGAG----CTACA--ATTTCATCAAAC-----------TTGATGTCTGTGC----------
DlonOBP12    TTGAAATACAACATTTATTGGCGATGATTGAAATTTTCTTGAG----CTACA--ATTTCATCAAAC-----------TTGATGTCTGTGC----------

                 710       720       730       740       750       760       770       780       790       800
             ....|....|....|....|....|....|....|....|....|....|....|....|....|....|....|....|....|....|....|....|
DlonOBP9     -----CAACTGATTGGTCCAATTGAGGAAAAAAATATGAAGGGGATTTTTTA-----------------TGGTTGAATTATTCAAATGAATCAATTAATT
DlonOBP11    TGGGGCAGATCATTTTGGGTTGATTGAAATATATGTCGAAGGATAAATCTCATATAGGCCTCAAATATATCATTTTATGATTGAAAAAGATGTGAAATAT
DlonOBP10    --AGATAAATAAATTTTGATTTTTAGAAAT-ATTGTCGCTGAGGAGTTGGCCTCTGGCATAGTAAT---TAGATAGGCTATTGAGGCCCATCGGAAAAGT
DlonOBP12    --AGATAAATAAATTTTGATTTTTAGAAAT-ATTGTCGCTGAGGAGTTGGCCTCTGGCATAGTAAT---TAGATAGGCTATTGAGGCCCATCGGAAAAGT

                 810       820       830       840       850       860       870       880
             ....|....|....|....|....|....|....|....|....|....|....|....|....|....|....|....|..
DlonOBP9     T------ACAA-----TGAAAATAATTAAATGAATG----------------------------------------------
DlonOBP11    ATGTATTTCCCTGACTTGTCATTTGTCACATATATTTTACAGATATTTTGCACACCACAGCGACATTAT-TGGTAGCTTTGTGTTTT
DlonOBP10    TGGTTGAACCA--ACCAGAGAAAATTCGCC-ATCGCTGATCAATTTTTGGACGGATAAATCGATATTGGGTTATGGATTTGAA----
DlonOBP12    TGGTTGAACCA--ACCAGAGAAAATTCGCC-ATCGCTGATCAATTTTTGGACGGATAAATCGATATTGGGTTATGGATTTGAA----
```

B

```
                   10        20        30        40        50        60        70        80        90       100
           ....|....|....|....|....|....|....|....|....|....|....|....|....|....|....|....|....|....|....|....|
DlonCSP3   ----------------------------------------------------------------------------------------------------
DlonCSP10  CGGTAATCTTACTATTACTTTTGGAATCTCATCCCTACGATGAAATCTCTAGTATTTTTCGCACTGGTGCTTGGGAGAGTCATTTTAATGGCGTCGGGGG
DlonCSP11  ------TCTTACTA-TACTTTTGGAATCTCATCCCTACGATGAAATCTCTAGTATTTTTCGCACTGGTGCTTGGGAGAGTCATTTTAATGGCGTCGGGGG

                  110       120       130       140       150       160       170       180       190       200
           ....|....|....|....|....|....|....|....|....|....|....|....|....|....|....|....|....|....|....|
DlonCSP3   --GTAAATATGGATCGATATGATTCAGTCAATGTTGAACAAGTATTGGGCAATGAGAGGATCTATACGCAGCATTTGAACTGTTTACTTGATCAAGGACC
DlonCSP10  ACGTAAATATGGATCGATATGATTCAGTCAATGTTGAACAAGTATTGGGCAATGAGAGGATCTATACGCAACATTTGAGCTGTTTACTTGATCAAGGACC
DlonCSP11  ACGTAAATATGGATCGATATGATTCAGTCAATGTTGAACAAGTATTGGGCAATGAGAGGATCTATACGCAACATTTGAGCTGTTTACTTGATCAAGGACC

                  210       220       230       240       250       260       270       280       290       300
           ....|....|....|....|....|....|....|....|....|....|....|....|....|....|....|....|....|....|....|
DlonCSP3   ATGTAGTCGTCAAGCTCAGTCCCTCAAGGACGTTTTACCTGAGGTCTTGAGAACCAGCTGCGCCACATGCAGTCCGGTACAAAAGAAAATGGCCCGGAAG
DlonCSP10  ATGTAGTCGTCAAGCTCAGTCCCTCAAGGACGTTTTACCTGAGGTCTTGAGAACCAGCTGCGCCACATGCAGTCCGGTACAAAAGAAAATGGCCCGGAAG
DlonCSP11  ATGTAGTCGTCAAGCTCAGTCCCTCAAGGACGTTTTACCTGAGGTCTTGAGAACCAGCTGCGCCACATGCAGTCCGGTACAAAAGAAAATGGCCCGGAAG

                  310       320       330       340       350       360       370       380       390       400
           ....|....|....|....|....|....|....|....|....|....|....|....|....|....|....|....|....|....|....|
DlonCSP3   GTGATCACATACATTCAGGCAAATAAACCCGGTGATTGGAACCTACTCACAGCAAAATACGATCCCAAAGGTTCCTACACGGAAGAAAT-CAAGAAATTT
DlonCSP10  GTGATCACATACATTCAGGCAAATAAACCCGGTGATTGGAACCTACTCACAGCAAAATACGATCCCAAAGGTTCCTACATGGAAGAAATACAAGAAATTT
DlonCSP11  GTGATCACATACATTCAGGCAAATAAACCCGGTGATTGGAACCTACTCACAGCAAAATACGATCCCAAAGGTTCCTACATGGAAGAAAT-CAAGAAATTT

                  410       420       430       440       450       460       470       480       490       500
           ....|....|....|....|....|....|....|....|....|....|....|....|....|....|....|....|....|....|....|
DlonCSP3   CTGGCCAGTAATCTGTAATTGATCTTATCAATGCCTTAGCAATGCGGAAAAATCCATTCGATTAAA-TCGATAGCTGCAATTGGAATTCACCATTAGTCG
DlonCSP10  AC--------------------------------------------------------------------------------------------------
DlonCSP11  CTGGCCAGTAATCTGTAATTGATCTTATCAATGCCTTAGCAATGCGGAAAAATCCATTCGATTAAAATCAACAGCTGCAATTGGACTTCACCATTAGTCG

                  510       520       530       540       550       560
           ....|....|....|....|....|....|....|....|....|....|....|....|
DlonCSP3   AAACCAAAAGAATCCGCATTTACTTTTTTTCATCCAGAAGGTGTTGTACTCTCGACAATG
DlonCSP10  -----------------------------------------------------------
DlonCSP11  AAACCAAAAGAATC----------------------------------------------
```
