# Supplementary material for: Identification and characterization of soluble binding proteins associated with host foraging in the parasitoid wasp Diachasmimorpha longicaudata
Source: PLoS One. 2021 Jun 17;16(6):e0252765. doi: 10.1371/journal.pone.0252765 (PMC8211293; doi:10.1371/journal.pone.0252765)
Supplement: S2 Fig — Multiple protein sequence alignment of soluble binding proteins from D. longicaudata and closely related species: (A) CSPs, the four conserved cysteine residues are marked in black. (B) OBPs, the six conserved cysteine residues are marked in black. (C) NPC2-like proteins, the seven conserved cysteine residues are marked in black. Alignments were performed by Clustal Ω, only the closer orthologous sequence to the D. longicaudata sequences (according to phylogenetic analysis) were used to improve the visualization of conserved regions. Identical and positive amino acids are highlighted in dark gray and light gray, respectively. Predicted signal peptides are highlighted in red. A. mellifera (Amel); N. vitripennis (Nvit); F. arisanus (Fari); D. alloeum (Dall); M. demolitor (Mdem) and D. longicaudata (Dlon). (PDF) [file pone.0252765.s002.pdf]

A

```
                                   10        20        30        40        50        60        70        80        90       100
                            ....|....|....|....|....|....|....|....|....|....|....|....|....|....|....|....|....|....|....|....|
DlonCSP_9(isotig_07729)     -----MFRGAVIIVL---------VFLSAVIAEEKYGDKYDHIDVDGILENDRQRETYYQCFAGTGPCKTAAARFFRDTLPEAIVTRCKKCTERQTINFD
DallCSP(XP_015117987.1)     -----MLRGAVVIAL---------VFLSAVIAEEKYSEKYDYVDVDGILANDKQRESYYKCFAGIGPCKTAAARFFRDTLPEAIVTRCKKCTARQSVNFD
DlonCSP_1(isotig_06173)     ----MKMQTILVFVATLFVVIG-----------------AHDLGVETFLKDKTFVNKEIDCMLNKAPCDII-GQMIKDILPDALNNDCGHCSSIQAKIMR
DallCSP(XP_015112195.1)     ----MKARMALFLVGMLSLVVG-----------------IEAQDVEALLKNPEFVNFEINCMLDEGPCDLI-GNSIKNVLPEALNNNCRRCTRSQARIIR
DlonCSP_8(HWR9CMJ02HQRKL)   ----------------------RDIDCYTWPRRNTYMTRWDKVNLDEILQNKRLLHHYFRCLMGVGPCPPD-GQELKRVLPEALETACAKCSKSQKERRD
DallCSP(XP_015118031.1)     MEAKPSTQVLVFFLFTV-VILTRDIECYTWPRRNTYMTRWDKVNLDEILQNKRLLHHYFRCLMGVGPCPPD-GQELKRVLPEALETACAKCSKSQKEGAI
DlonCSP_6(isotig_07750)     ------MK--VA-------IVLLAVVAVTLGKPQGYTTKYDNVDLDQILRNDRLLNNYVKCLLDEGNCTSD-GKELKASLPDALATGCNKCSEKQRVGSE
DallCSP(XP_015123554.1)     ------MK--VA-------FVLLAVVAVSLAKPQGYTTKYDNVDLDQILRNDRLLNNYVKCLLDEGHCTSD-GKELKASLPDALATGCTKCSEKQRAGSE
DlonCSP_5(isotig_00294)     ------MKIAMF-------VLLSCLV-VITARPESYTTKWDNIDINQILNNDRILNNYVNCLLEKGNCTPD-GRELKSVLPDALETECTKCSQKQKDGSK
DallCSP(XP_015123508.1)     ------MKIAVF-------VLLSCLVAVISARPDKYTTKWDNIDVDQILNNDRILNNYVNCLLEEGNCTAE-GRELKSVLPDALETECEKCSRKQRDGSK
NvitCSP(NV16075-PA)         ------MRPAFYISLM---VLG--WAVSLSLAGDPFA------RVNDILSNETTLQFYAKCFLDQGPCSGD-GRAIKRLLPDFISSSCARCSSRQKQMAC
DlonCSP_2(HWR9CMJ02G3Q4Q)   ------------------------------------------------------------------------GRQLKAALPEVILRNCRNCSPQQAQNAQ
DallCSP(XP_015117985.1)     ------MKSSVFFVLA---ILG--AAFIAAEGGNRYADKYDSVNVDQLLGNERIYKQHLNCLLDQGQCSRQ-AQSLKDVLPEVLSTSCAKCSPVQRQMAR
DlonCSP_10(HWR9CMJ02F3EDJ)  ------MKSLVFFALV----LG--RV-ILMASGDVNMDRYDSVNVEQVLGNERIYTQHLSCLLDQGPCSRQ-AQSLKDVLPEVLRTSCATCSPVQKKMAR
DlonCSP_3(HWR9CMJ02FPWJW)   ----------------------------------VNMDRYDSVNVEQVLGNERIYTQHLNCLLDQGPCSRQ-AQSLKDVLPEVLRTSCATCSPVQKKMAR
DlonCSP_11(HWR9CMJ02GGN00)  ------MKSLVFFALV----LG--RV-ILMASGDVNMDRYDSVNVEQVLGNERIYTQHLSCLLDQGPCSRQ-AQSLKDVLPEVLRTSCATCSPVQKKMAR
                                                                                        C1     C2                  C3 C4

                                  110       120       130       140
                            ....|....|....|....|....|....|....|....|....|..
DlonCSP_9(isotig_07729)     KISEW-YTKNEPEKYQIIVAKAVRDIMARSA----------------
DallCSP(XP_015117987.1)     KISDW-YTTNEPEKYQIIVAKAVRDIMAKSA----------------
DlonCSP_1(isotig_06173)     RLMDF-METSHPELNRQIRELYPPNQG--------------------
DallCSP(XP_015112195.1)     RLIDF-METAYPEQNQRIRNRYIRSPTSELA--DELP----------
DlonCSP_8(HWR9CMJ02HQRKL)   LRN--------------------------------------------
DallCSP(XP_015118031.1)     YVIKY-LREYMPKKLEMLANRYDPDGKYRRRYYHSTSV--DNNTT--
DlonCSP_6(isotig_07750)     KVIRY-LVNERPKVWQKLAAKYDPHDEYRVKFQGEANA--RGIKV--
DallCSP(XP_015123554.1)     KVIRY-LVNERPKVWQKLAAKYDPHDEYRVKFQGEASA--RGIQV--
DlonCSP_5(isotig_00294)     RIIKF-LVRNKPDLWEKLMEKYDADKKYRGKYEDQAKA--EGIEIRG
DallCSP(XP_015123508.1)     KIIKF-LVQNKQDLWEKLMDKYDEEKKYRGKYEDQARA--EGIEIQS
NvitCSP(NV16075-PA)         KILYTLQQEKYADLWVDFVKKYDPVGQHQTKLQNFRELCLDSFSIAV
DlonCSP_2(HWR9CMJ02G3Q4Q)   KLTNF-LQTRYPDVWAMLLKKYQNA----------------------
DallCSP(XP_015117985.1)     KVVGY-IQKNKPDDWKLLTTKFDPQGRYTEEIRRFILSNV-------
DlonCSP_10(HWR9CMJ02F3EDJ)  KVITY-IQANKPGDWNLLTAKYDPKGSYMEEIQEIY-----------
DlonCSP_3(HWR9CMJ02FPWJW)   KVITY-IQANKPGDWNLLTAKYDPKGSYTEEIKKFLASNL-------
DlonCSP_11(HWR9CMJ02GGN00)  KVITY-IQANKPGDWNLLTAKYDPKGSYMEEIKKFLASNL-------
```

B

```
                                     10        20        30        40        50        60        70        80        90       100
                                 ....|....|....|....|....|....|....|....|....|....|....|....|....|....|....|....|....|....|....|....|
DlonOBP_7(isotig_07880)          MS--GIHQL-----LLILV-----------------VG--GV---------------SCSP--D---DCTVSQEWRDAKE-----ECQKEMDMDDECFW
DallOBP(XP_015114447.1)          MG--PIHQL-----LAIFV-----------------LG--AL---------------SCFA--E---DCKTSRKWLAAKE-----ECEKEMSEQNACFW
FariOBP(XP_011313771.1)          MA--LLIHL-----LGICV-----------------LI--SL---------------NCLAK-Q---NCTVNHEWKKARE-----ECEKEMDKTDDCFW
DallOBP-X2(XP_015114453.1)       M--------------------------------------TA----------------NLVS--H---GCKL------EKT-----VEGRVVSEDDECFW
DallOBP-X1(XP_015114451.1)       MC--RIYQL-----VGIFV-----------------LG--AF---------------NCIA--D---DCKSSEPWVQARE-----NCRRQISEDDECFW
DlonOBP_1(isotig_05963)          ----------------MRI------------IGVSLALLS---------LVLFVRGDDKDPHGPIREKCKDQFGLSSDDLKAAM-----EDPSDVGCYI
DallOBP83alike(XP_015111194.1)   MQRENF-KRQGTG-IDMRI------------IVPCLALLS---------LVLFVRGDDKDPHGPIREKCKDQFGLSSEDLKEAM-----EDPNDVGCYL
DlonOBP_14(HWR9CMJ02H/7WK0)      MRKYV--GF-------LCLILQV-----------SIGHCG------PVGRPDFVSDEMIALAASVVNACQTQTGVATADIEAVR-NGQWLNSTPLKCYM
FariPBP6like-2(XP_011299164.1)   MNKFI--GT-------LCLVLQV-----------GIAFSG------PVGRPDFVSDDMIALAASVVNACQTQTGVATADIEAVR-SGQWPDSTPLKCYM
DlonOBP_6(isotig_05241)          MARHMV----------CCFLIGM-----------AMQALLV-----SAGRPDFITDDMMAMVADDKARCMSEHGTTEALIDEVN-TGALPNNRALTCYM
DallOBP69alike(XP_015122793.1)   MARHVV----------CCFLLGV-----------AMQALIV-----SAGRPDFITDDMMAMVADDKARCMGEHGTTETLIDEVN-NGALPNDRALTCYM
DlonOBP_4(isotig_06993)          MKSAFISGL------VIVLVVL------------NFDNVGA----------KMTIPQVTNMLMPMRKTCMQKTGASAELVDAPK-TGPMPDDPVLQCYY
DallOBP69alike(XP_015115396.1)   MKSGILLGL------AILLVAQ------------SFENAEA----------KMTIPQVTNMLMPMRKTCIQKTGASADLVDAPK-TGPMPDDPSLKCYY
FariOBP69alike(XP_011301198.1)   MKSGIFLGL------AIFFVAQ------------NYQNAEA----------KMTIPQVTNMLVPMRKTCTQKIGVAPELIDAPK-TGPMPDDPTLKCYY
DlonOBP_5(isotig_07011)          MAKHILSAI-----ILFCLL--------------SYIST------------GPIPKEFQEVAGDIRKVCIEETGTTVDLIERAG-KGDFAEDDKLKCYL
FariOBP83alike(XP_011299167.1)   MVKYILSAV-----VLVFLV--------------GYISA------------GPIPKEFQEVAGDIRKVCIAETGTTVDLIERAG-KGDFADDENLKCYL
DallOBP83alike(XP_015118617.1)   MVKYMLSAV-----VLLCLM--------------GYISA------------GPIPKEFQEVAGDIRKVCIEETGTTVDLIERAG-KGDFAEDDNLKCYL
DlonOBP_8(isotig_02295)          MVKYIVSAF-----IAVCLV--------------AAIRA------------GEIPPEFKEIAPEVRRVCLEESGAENEWVVKAN-KGDFTDDPKFKCYL
FariOBP56dlike(XP_011299086.1)   MIKNIVYAL-----VTVCLV--------------AAINA------------GEIPEEFREIAPEVRRVCLEETGAEVEWIEKAN-KGEFTDDQKFKCYL
DallOBP83alike(XP_015109650.1)   MVKYVISAS-----LAVCLV--------------AVINA------------GEIPPEFKEIAPEVRRVCLEETGAKVEWIEKAN-KGEFTDDPKFKCYL
DlonOBP_17(isotig_07052)         MK--LLVIL-----LI--------------------FHVAFAAGAF---------RPADIVRFQRAVEKCRISEDVSDEVLERVL-NGEIVDDPHFNCFA
DallOBP(XP_015114445.1)          MK--LLVAV-----LI--------------------LSVAFVSAAF---------TAADIVRFQRTLEKCRTKNDLSDEVLERVL-NGEIVDEPDFNCFA
DlonOBP_3(isotig_07569)          MN--TSTVV-----LVFCA-----------------LAVTFVSGGD------------MKAEMHAQVEKCIIETGVDPSVLKSLHETGGANADENVKCLG
DallOBP69alike(XP_015125081.1)   MN--TSATV-----LVFCA-----------------LAVTLVLGHH------------PKPMFAAAAEKCIEKTGVDLDALKTLHETGGVNADEKLKCFG
DlonOBP_2(isotig_07924)          MKSGL---------FVVAL-----VG----------AFAV--AAGGPP------DKDCPLMKALKESIDACIDKLSEE--SVKLME-KDAFADNEEIRCFH
DallOBP56alike(XP_015115397.1)   MKSGV---------FFVAL-----VG----------AFAV--VAGDPP------NKDCPLMKALKESIDACIDKLSEE--SAKLME-KDQFADNEEIRCFH
DlonOBP_9(isotig_01415)          MK--FLVIA-----VLVCI-----VG----------ALASEQ--------------RETPEARRQRYRDACRAETGADQADIDKAR-KGEWADKENLRCYT
FariOBP56alike(XP_011311566.1)   MK--VFLVL-----FSVCL-----AA----------VMAQQI--------------SDIQKETLRENRDACITETGADRAEVDNAY-KNEWVDNEKLRCFA
DlonOBP_11(isotig_03729)         MK--FLVVV-----IFVCL-----AG----------ALAQEL--------------TEAQRRRLREHRDICIRETDANRAEVDRAR-QGQWADNPQIRCFA
DallOBP56d-2(XP_015113058.1)     MK--FLVVL-----FFVCL-----VG----------ALAQEL--------------NEAQRQRLREHRDACIRETGADRAEVDRAH-RGDWADNPQIRCFA
DallOBP56d-1(XP_015125079.1)     MK--FLVIV-----FFICL-----VG----------ALAQEL--------------TDAQKQKLREHRDACVTETGADRADVDKAY-KGEWADNPKLRCFT
DlonOBP_10(isotig_01518)         MSR-SNVTLPR-TGTDCLN---IISIIFIHLH-VKAQEL--------------TEAQKQKVRENRDACITETGADREEVNKAN-KGEWADNAKIRCFT
DlonOBP_12(isotig_01519)         MK--VFVVI-----LSTCL-----IG----------ALAQEL--------------TEAQKQKVRENRDACITETGADREEVNKAN-KGEWADNAKIRCFT
                                                                                                       C1                           C2

                                    110       120       130       140       150       160       170       180       190
                                 ....|....|....|....|....|....|....|....|....|....|....|....|....|....|....|....|....|....|....|
DlonOBP_7(isotig_07880)          RCTHDEIHVLNDAGELSEDIIRNVFRKLVDGAPLQEALDKAASRCFSE-IG-DEVKGCDQSAMFHKCLWKDQDYVQAAKDANHCIEIV------
DallOBP(XP_015114447.1)          NCMYEEVRALDTYGRLSKERSPDALKKVIEEPELREAVDHETIASCLSQ-VR-DEIDPCNQSKMLHECLWSDEEFLEAMKAANHCVMTPQ-----
FariOBP(XP_011313771.1)          NCVQGELGGLDDSGRPSLEKLSEAVAKVIKEPDVVEALRKSHEECISQ-FN-DTMTPCEESKLLNVCSWGDAELQQALQGANYCVYNPANPS---
DallOBP-X2(XP_015114453.1)       NCVHGTLGILDDSGRLSEDKIHDVVEKLIDEPELREVMEKSQTNCISQ-ID-DTMNACKGSKMFNKCSWSDEEVVQALKAENYCQEDFEDLIKNQ
DallOBP-X1(XP_015114451.1)       NCVHGTLGILDDSGRLSEDKIHDVVEKLIDEPELREVMEKSQTNCISQ-ID-DTMNACKGSKMFNKCSWSDEEVVQALKAENYCQEDFEDLIKNQ
DlonOBP_1(isotig_05963)          LCFFKDLSIMDDSGKFDPDAALDA--IEDSAKD---DAKPVLSSCDSKVKKSTTKDPCARALEVVTCFKEEAP--ELYKNLGIFHPLG------
DallOBP83alike(XP_015111194.1)   LCFFKDLSIMDDSGEFDPLAAMDA--IEESAKD---GARPVIFSCYDQEKKSATKDTCARALEVVLCFKKEAP--ELYKNLGLFHPLGQ------
DlonOBP_14(HWR9CMJ02H/7WK0)      YCLWEQFGLVDDKRELSLNGMLTFFQRIPAYRA---EVQTAIRECK---AD---GDNCQYAYTFNLCYSKLSP--------RTYYLF--------
FariPBP6like-2(XP_011299164.1)   YCLWEQFGLVDDKRELSLNGMLTFFQRIPAYRA---EVQTAIRECKEI-AN---GDNCQYAYTFNLCYAKLSP--------RTYYLF--------
DlonOBP_6(isotig_05241)          DCLFAAFGVIDEGE-LEVDMLVGF---LPDHMQD--AARDLLETCAKQ-PG---ADPCDKVFEIAKCVQAKRP--------DLWFMI--------
DallOBP69alike(XP_015122793.1)   DCLFAAFGVIDEGE-LEVDMLVGF---LPDHMQD--QARELLEACAKQ-PG---ADPCDKVFNIAKCVQAKRP--------DLWFMI--------
DlonOBP_4(isotig_06993)          ACLLKMIKVLTKEGLPNVDSMVKQMDVMLPADDITARLKEVIALCTPA---VTSTDSCEGTWQFIKCFYETDK--------NVCFFP--------
DallOBP69alike(XP_015115396.1)   SCLLKMVKVVTKEGLPNYENMVKQMDMMLPADDLTARLKDVINLCAPK---VTSTEPCEANWQFIKCFYETDK--------NVCFFP--------
FariOBP69alike(XP_011301198.1)   ACLLKMIKVVTKEGLPNYESMLKQMDIMLPADDTTARLKEVINICAPQ---VTSTEACDGTWQFIKCFYETDK--------NVCFFP--------
DlonOBP_5(isotig_07011)          KCLFAQFGLISKKG-LNFEQLVKV--APPDMKD---MAKQLGVTCKDI-KMNAAGDQCDLSYHMSKCFFNTFP-DQ-------YFIM--------
FariOBP83alike(XP_011299167.1)   KCLLAQFGLISKKG-LNFEQLVKV--APPDMKD---MAKQLGVTCKDI-KINEAGDQCDLAYHMSKCFFNTFP-EQ-------YFIM--------
DallOBP83alike(XP_015118617.1)   KCLFAQFGLISKKG-LNFEQLVKV--APPDMKD---MAKQLGVTCKDI-KINADGDQCDLAYHMSKCFFNTFP-DQ-------YFIM--------
DlonOBP_8(isotig_02295)          KCTLAQFGAVSRKG-VNFDALTKL--APPAYKE---ILDKVISACKDT-KPTIPGDVCDQVYEASKCFYRAAP--------DNYFVM--------
FariOBP56dlike(XP_011299086.1)   KCTVAQFGAVSRKG-VNFDALAKL--APPAYKT---KLEQIVAACKDT-KGTKPGDVCDQVYEVSKCFYRTAP--------DVYFVM--------
DallOBP83alike(XP_015109650.1)   KCTVAQFGAVSRKG-VNFDALAKL--APPAYKE---LKLEKIIAVCKDT-KGTIPGDVCDQVYESSKCFQRTSP--------DDYFVM--------
DlonOBP_17(isotig_07052)         ACLLEEFQLLKPDGSFNDELAVTK---IPQDADFAQQLTDAIKTCSAR-RD---PDKCTTAHLLFVCMYENNI-------PTLLFG---------
DallOBP(XP_015114445.1)          ACLLQNYELLREDGSFNVELAVSK---IPQDADFAQPLTDAIKTCSAR-RG---TDNCKTAHLLFVCMYEKDV-------PNLLFG---------
DlonOBP_3(isotig_07569)          ACIMKGLGVMAADGTVNLDKAKSL---VPSDAPDHDALLAAVEECHAE-KG---ANDCETAHAIGMCMHKKHV--------GPMD----------
DallOBP69alike(XP_015125081.1)   ACIMKGLGVMAEDGTVDVEAAKEL---VPSDVPDRDKIVAVIEACHHE-KG---ANECETAHAIGMCMHKNHM--------NDMQN---------
DlonOBP_2(isotig_07924)          ACVMTHSGLMTDGK-MDIPALEEMLSGNPDDEEEAQKIIEIMKMCKPE-AE-ISDNECEVAGNYVKCFQAKKV--------N-------------
DallOBP56alike(XP_015115397.1)   ACVMTHSGLMTDGK-MDA-LLEEMLSGDPEHEEEARKITEIMKSCKPE-SE-TSDNECEVAGNYVKCFQAKKM--------N-------------
DlonOBP_9(isotig_01415)          LCMLKKGNLMNDEGVLDEIVT-RDTLARKLPAE---QINDIMAKCKDL-KG---SNACGTAQRIMKCYNEQKT--------HLM-----------
FariOBP56alike(XP_011311566.1)   LCLLKKWKMMDDQGKLDETMA-RERMGKVMPPA---KVDELMTKCKDL-KG---SNPCETGYMMMKCYTGNIA-------PAAV-----------
DlonOBP_11(isotig_03729)         LCMMRRLRLMSEDGQLNEAAA-RQRLALVVPRE---RVEEIMTKCKDL-KG---NTPCDTGYLVLKCYTDNRA--------NVV-----------
DallOBP56d-2(XP_015113058.1)     LCMMKRLGLMSDDGKLNETAA-RQRLALVLRRE---RVEEVMRKCKDL-KG---PTPCDTGYLILKCYTDNRA--------NMV-----------
DallOBP56d-1(XP_015125079.1)     LCMMKKVGMMNDDGVLDETIT-RQRMALKLKPE---KVDEIWTKCKDL-KG---TTACDTAYMMMKCYTENRA--------ITV-----------
DlonOBP_10(isotig_01518)         LCMLKKWGMMNDAGVLDEAAA-RQKMGLQMKPE---KVEEIMTKCKYL-KG---DTACDTAYMMMKCYTDNRA--------VTV-----------
DlonOBP_12(isotig_01519)         LCMLKKWGMMNDAGVLDEAAA-RQKMGLQMKPE---KVEEIMTKCKYL-KG---DTACDTAYMMMKCYTDNRA--------VTV-----------
                                  C3                                         C4           C5        C6
```

C

```
                                   10        20        30        40        50        60        70        80        90       100
                               ....|....|....|....|....|....|....|....|....|....|....|....|....|....|....|....|....|....|....|....|
Dlon_isotig.05505              ------MKSFLVIFGVVCVALVRAEVVQFQPCVYPEGTPINCTVHELRIKPCPESAAAKPCRVKRGTDASIEFDYSTNFSANTLQGRAYWANSLVDLPFL
DallMD-2(XP_015114239.1)       ------MKNFLVILGVACVALVGADVVQFQPCSYREPTQTNCTVHELRINPCAEAAEGKPCRVKRGTDASIEFDYTTNFAADTLQGRAYWANKLMDVPFL
Dlon_isotig.06291              MKEMLGLKVLV-IV-GFCVAAS-LQDTPFTKCKTGGPPP-----ESLRIKGCN----TTPCKFIKGTDIKAEWDFNVVADTDDLHPKVLVKVAGFTIDYP
DallNPC2b(XP_015120673.1)      ---MLGLKVLV-IV-GFCVAAS-LQDTPFTKCKSGGPAP-----EGLRVKGCD----KTPCKFAKGTNVEAEWDFNVVADTDDLKPIVKARALGITVNYP
FariNPC2b(XP_011305854.1)      ---MLGLRVLI-IA-AFCVVGS-LQDTPFTKCKAGGPAP-----EALRVKGCN----TTPCRFVKGKDVEAEWDFNVVADAEALKPVVKAKALGITVDYP
Dlon_isotig.07456              ------MSRIICAF-VVCLSTL-VSAAKFRDC--GSAVG---KYTSVTISNCGA--SDSECILTRGSNATIEISFNTDESADAVTAVVHGIVASVPMPYP
FariNPC2a(XP_011300999.1)      ------MYPIICAL-LVCLSVL-TSAADFRDC--GSQVG---KYTSISISDCED--SDSECILTKGTNVTIKITFNTDEPVNVVKAVVHGILTGVPIPFP
DallNPC2a(XP_015119973.1)      ------MCRTICAL-VVCLSAL-ASAAEFKDC--GSSVG---KYSSISISDCGA--SDSECILTKGTNATIEISFSTVEPVNAVKAVVHGILTGVPIPFP
Dlon_isotig.07261              ---MFGKTAILFTT-VLCFVTF-GESTDVLKCKNGGSFD---TIKSIVITKCE----QPPCLLKRGTRVELVQQFVPEKNVEKLTTSVHATLLGVPLPFV
DallESR16(XP_015109258.1)      ---MFGKTALVFTT-VLCFVAF-GESTDVIECKNGRNLD---NIKSITITNCE----EPPCELKRGTRVELVQKFVPNEDVDRLTTSVSATILGVRLPFI
                                                 C1            C2                  C3        C4
                                  110       120       130       140       150       160       170
                               ....|....|....|....|....|....|....|....|....|....|....|....|....|....|....|
Dlon_isotig.05505              G-MTTDACLS-------TPCPVNPDTKQTYKMSLHIDHKYPARSYDVKWKLWNQQEQ-ECCIVFQIKLVK-----
DallMD-2(XP_015114239.1)       G-METNACLS-------TPCPLKADTKQTYKMALHVDTKYPARSFDVKWKLWNQQEQ-ECCIVFQIKLTK-----
Dlon_isotig.06291              L-PEQDACKSL----TNGECPLEKGELVSYGLKMPILKMYPSTKLHLTFSLVDQHKNTQVCFEVDAAVID-----
DallNPC2b(XP_015120673.1)      L-PEQDACKSL----TNGECPLEKGELISYGLKMPILKAYPNVDLHLTFSLVDQKENVHVCFELDARVVN-----
FariNPC2b(XP_011305854.1)      L-PEQDACKSL----VNGECPLEKGELVSYGLRMPILKAYPKVDLHLTFFLVDPQKNVHVCFEIDAKVVDN----
Dlon_isotig.07456              I-SHPDACAN---PDTGITCPLKKGGSYSYRKTFPVLAQYPKVRVQVKWELQNEKMQDIICILIPAKIQ------
FariNPC2a(XP_011300999.1)      I-AQPNACNN---PETGITCPLKKGGPFTYTKTFPILSQYPKVKVEVKWELQNEKNQDIVCIMIPARIE------
DallNPC2a(XP_015119973.1)      I-SHPDACAN---PDTGITCPLKKDQAYTYRKTFPILAQYPKVKVEVKWELQNEKRQDIVCILIPAKIQEKKSAN
Dlon_isotig.07261              GVDGTDACPNIFDADGKPGCPLKAGTQYYYKNGFPVLNFYPTVDLVVAWALLEN-NVPLTCFEIPAKIIR-----
DallESR16(XP_015109258.1)      GVDGSDACQNVYDADGKVGCPLKAGTEYYYKNGFPVLEIYPKVKLVVSWALLGS-SNPITCFEIPSKITS-----
                                      C5          C6                                      C7
```
