# Supplementary figures and images for: Identification and characterization of soluble binding proteins associated with host foraging in the parasitoid wasp Diachasmimorpha longicaudata
Source: PLoS One. 2021 Jun 17;16(6):e0252765. doi: 10.1371/journal.pone.0252765 (PMC8211293; doi:10.1371/journal.pone.0252765)

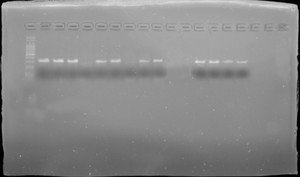

Supplement: S3 Fig — From left to right: molecular-weight size marker, CSPs 1–9, NPC2a, NPC2b, ESR16 and MD-2. (TIF) [file pone.0252765.s003.tif]

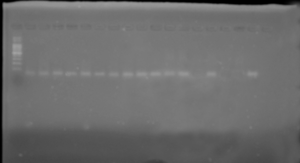

Supplement: S4 Fig — From left to right: molecular-weight size marker, OBPs 1–17. (TIF) [file pone.0252765.s004.tif]

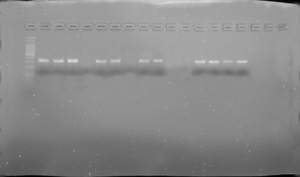

Supplement: S5 Fig — From left to right: molecular-weight size marker, CSPs 1–9 and NPC2a, NPC2b, ESR16, and MD-2. (TIF) [file pone.0252765.s005.tif]

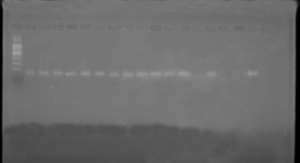

Supplement: S6 Fig — From left to right: molecular-weight size marker, OBPs 1–17. (TIF) [file pone.0252765.s006.tif]

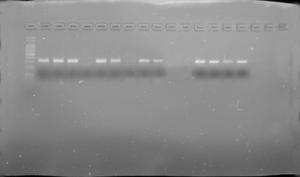

Supplement: S7 Fig — From left to right: molecular-weight size marker, CPSs 1–9 and NPC2a, NPC2b, ESR16 and MD-2. (TIF) [file pone.0252765.s007.tif]

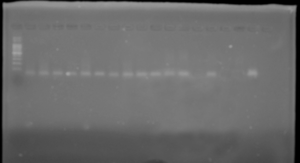

Supplement: S8 Fig — From left to right: molecular-weight size marker, OBPs 1–17. (TIF) [file pone.0252765.s008.tif]

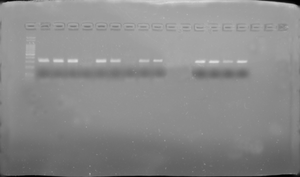

Supplement: S9 Fig — From left to right: molecular-weight size marker, CSPs 1–9 and NPC2a, NPC2b, ESR16 and MD-2. (TIF) [file pone.0252765.s009.tif]

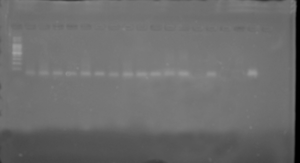

Supplement: S10 Fig — From left to right: molecular-weight size marker, OBPs_ from left to right MW, OBPs 1–17. (TIF) [file pone.0252765.s010.tif]

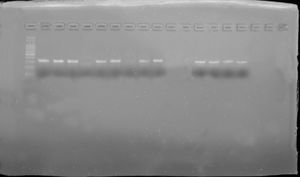

Supplement: S11 Fig — From left to right: molecular-weight size marker, CSPs 1–9 and NPC2a, NPC2b, ESR16 and MD-2. (TIF) [file pone.0252765.s011.tif]

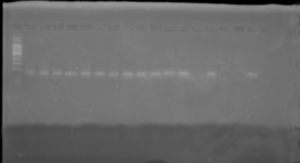

Supplement: S12 Fig — From left to right: molecular-weight size marker, OBPs 1–17. (TIF) [file pone.0252765.s012.tif]

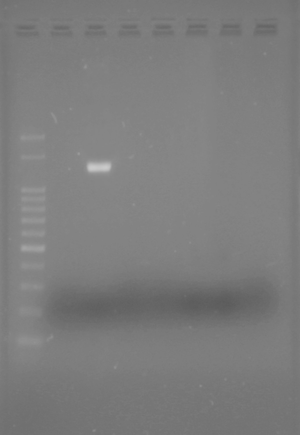

Supplement: S14 Fig — From left to right: molecular-weight size marker, negative control, β Actin as a positive control, CSPs 4 and 7, and OBPs 13, 15, and 16. (TIF) [file pone.0252765.s014.tif]
