# Supplementary material for: Identification and characterization of soluble binding proteins associated with host foraging in the parasitoid wasp Diachasmimorpha longicaudata
Source: PLoS One. 2021 Jun 17;16(6):e0252765. doi: 10.1371/journal.pone.0252765 (PMC8211293; doi:10.1371/journal.pone.0252765)
Supplement: S13 Fig — (PDF) [file pone.0252765.s013.pdf]

CSP 1

```
                             10        20        30        40        50        60        70        80        90       100
                    ....|....|....|....|....|....|....|....|....|....|....|....|....|....|....|....|....|....|....|....|
CSP1_Seq_Fw-Primer  ----------------------------------------------------------------------------------------------------
CSP1_Seq_Rv-Primer  ----------------------------------------------------------------------------------------------------
CSP1_cds            TTCCATTTAATAATTATGATAATAAGAACATTAGTGCGACAATGTACTGTACATCATAGAAGTTATTATTATATCGTAATTATGTAATAAATATTGTTGT

                            110       120       130       140       150       160       170       180       190       200
                    ....|....|....|....|....|....|....|....|....|....|....|....|....|....|....|....|....|....|....|....|
CSP1_Seq_Fw-Primer  ----------------------------------------------------------------------------------------------------
CSP1_Seq_Rv-Primer  ----------------------------------------------------------------------------------------------------
CSP1_cds            TAACATAAAATCATTGGTTTTGTCATTTATTGTGTAATTTTAATGGCCGGAAAACTGTATTTTTCATTGCGCAGCAGTTTAATCATCAAATCTCTCTTTG

                            210       220       230       240       250       260       270       280       290       300
                    ....|....|....|....|....|....|....|....|....|....|....|....|....|....|....|....|....|....|....|....|
CSP1_Seq_Fw-Primer  ----------------------------------------------------------------------------------------------------
CSP1_Seq_Rv-Primer  ---------------------------------------------------------------------------------------------TCTACCA
CSP1_cds            CCATTCGTCGCAATGACTAATTATCAGTACGAATAGTAGGAGTTGGCTTTTAGTCGCCACAGGCGAATAATTCTGGGAATAAATTTCAACTGGTCTACCA

                            310       320       330       340       350       360       370       380       390       400
                    ....|....|....|....|....|....|....|....|....|....|....|....|....|....|....|....|....|....|....|....|
CSP1_Seq_Fw-Primer  ------------------------------------------TATTTACTTTGTTTGTCGGTATCGTCTAGCCTTGGTTGGGGGGATATAATTCACGAAT
CSP1_Seq_Rv-Primer  GAGTCGAATAAAGATAATTGTAGTCAGTGCTGAGTGGCAAAATATTTACTTTGTTTGTCGGTATCGTCTAG-----------------------------
CSP1_cds            GAGTCGAATAAAGATAATTGTAGTCAGTGCTGAGTGGCAAAATATTTACTTTGTTTGTCGGTATCGTCTAGCCTTGGTTGGGGGGATATAATTCACGAAT

                            410       420       430       440       450       460       470       480       490       500
                    ....|....|....|....|....|....|....|....|....|....|....|....|....|....|....|....|....|....|....|....|
CSP1_Seq_Fw-Primer  TTGTCG----------------------------------------------------------------------------------------------
CSP1_Seq_Rv-Primer  ----------------------------------------------------------------------------------------------------
CSP1_cds            TTGTCGGTTTAGTTCGGGGTGGGAGGTCTCCATGAAGTCCATCAGACGACGCATGATCTTGGCTTGAATGGATGAGCAATGGCCACAATCATTGTTCAAT

                            510       520       530       540       550       560       570       580       590       600
                    ....|....|....|....|....|....|....|....|....|....|....|....|....|....|....|....|....|....|....|....|
CSP1_Seq_Fw-Primer  ----------------------------------------------------------------------------------------------------
CSP1_Seq_Rv-Primer  ----------------------------------------------------------------------------------------------------
CSP1_cds            GCATCGGGTAATATATCTTTGATCATTTGTCCAATTATATCGCAGGGTGCCTTGTTCAACATACAATCAATTTCTTTGTTAACAAATGTTTTGTCCTTCA

                            610       620       630       640       650       660       670       680       690       700
                    ....|....|....|....|....|....|....|....|....|....|....|....|....|....|....|....|....|....|....|....|
CSP1_Seq_Fw-Primer  ----------------------------------------------------------------------------------------------------
CSP1_Seq_Rv-Primer  ----------------------------------------------------------------------------------------------------
CSP1_cds            GGAAGGTTTCGACCCCCAAATCGTGGGCACCAATAACAACAAAGAGTGTTGCAACAAAAACCAAAATCGTTTGCATCTTCATTTTTATCCACAAACAGTT

                            710       720       730       740       750       760       770       780       790       800
                    ....|....|....|....|....|....|....|....|....|....|....|....|....|....|....|....|....|....|....|....|
CSP1_Seq_Fw-Primer  ----------------------------------------------------------------------------------------------------
CSP1_Seq_Rv-Primer  ---------------------------------------------------------------
CSP1_cds            GCTAATTCGCCAGCGAAATTCTCGATTGTGTAGAGTTATCGTCGGTTATCACTTGAATTAATT


                    ....|....
CSP1_Seq_Fw-Primer  ---------
CSP1_Seq_Rv-Primer
CSP1_cds
```

CSP 2

```
                                       10        20        30        40        50        60        70        80        90       100
                              ....|....|....|....|....|....|....|....|....|....|....|....|....|....|....|....|....|....|....|....|
CSP2_Seq_Fw-Primer            ------------------------------------------------TCGCAATTGCTCGCCTCAACAAGCTCAAAATGCGCAAAAATTAACAAATTTT
CSP2_Seq_Rv-Primer(Rev-Comp)  ----------------------------------------------------------------------------------------------------
CSP2_cds                      GGGAAGGCAGCTTAAAGCCGCCTTACCAGAAGTTATCTTAAGGAACTGTCGCAATTGCTCGCCTCAACAAGCTCAAAATGCGCAAAAATTAACAAATTTT

                                      110       120       130       140       150       160       170       180       190       200
                              ....|....|....|....|....|....|....|....|....|....|....|....|....|....|....|....|....|....|....|....|
CSP2_Seq_Fw-Primer            TTGCAAACTAGATACCCGGA--------------------------------------------------------------------------------
CSP2_Seq_Rv-Primer(Rev-Comp)  ---------------------GTGTGGGCTATGCTTCTTAAGAAATATCAAAACGCTTAGAACGGATATCTTCCAATAGAAATACCAACTGCTAAAAACA
CSP2_cds                      TTGCAAACTAGATACCCGGATGTGTGGGCTATGCTTCTTAAGAAATATCAAAACGCTTAGAACGGATATCTTCCAATAGAAATACCAACTGCTAAAAACA

                                      210       220       230       240       250       260       270       280       290       300
                              ....|....|....|....|....|....|....|....|....|....|....|....|....|....|....|....|....|....|....|....|
CSP2_Seq_Fw-Primer            -------------------------------
CSP2_Seq_Rv-Primer(Rev-Comp)  ACCAAACTTCATTTT-------------------------------------------------------------------------------------
CSP2_cds                      ACCAAACTTCATTTTTATATGATATTTTTAACTATGGCTTTTAAAAATTAGTCGGAATAAACATGTCTTACTAAAGGTGTAACTTGGTATCAACTTCTAC


                              .
CSP2_Seq_Fw-Primer
CSP2_Seq_Rv-Primer(Rev-Comp)  -
CSP2_cds                      C
```

CSP 3

```
                                       10        20        30        40        50        60        70        80        90       100
                              ....|....|....|....|....|....|....|....|....|....|....|....|....|....|....|....|....|....|....|....|
CSP3_Seq_Fw-Primer            ----------------------------------------------------------------------------------------------------
CSP3_Seq_Rv-Primer(Rev-Comp)  ----------------------------------------------------------------------------------------------------
CSP3_cds                      GTAAATATGGATCGATATGATTCAGTCAATGTTGAACAAGTATTGGGCAATGAGAGGATCTATACGCAGCATTTGAACTGTTTACTTGATCAAGGACCAT

                                      110       120       130       140       150       160       170       180       190       200
                              ....|....|....|....|....|....|....|....|....|....|....|....|....|....|....|....|....|....|....|....|
CSP3_Seq_Fw-Primer            ----------------------------------------------------------------------------------------------------
CSP3_Seq_Rv-Primer(Rev-Comp)  ----------------------------------------------------------------------------------------------------
CSP3_cds                      GTAGTCGTCAAGCTCAGTCCCTCAAGGACGTTTTACCTGAGGTCTTGAGAACCAGCTGCGCCACATGCAGTCCGGTACAAAAGAAAATGGCCCGGAAGGT

                                      210       220       230       240       250       260       270       280       290       300
                              ....|....|....|....|....|....|....|....|....|....|....|....|....|....|....|....|....|....|....|....|
CSP3_Seq_Fw-Primer            ----------------------------------------------------------------------------------------------------
CSP3_Seq_Rv-Primer(Rev-Comp)  ----------------------------------------------------------------------------------------------------
CSP3_cds                      GATCACATACATTCAGGCAAATAAACCCGGTGATTGGAACCTACTCACAGCAAAATACGATCCCAAAGGTTCCTACACGGAAGAAATCAAGAAATTTCTG

                                      310       320       330       340       350       360       370       380       390       400
                              ....|....|....|....|....|....|....|....|....|....|....|....|....|....|....|....|....|....|....|....|
CSP3_Seq_Fw-Primer            ----------------------------------------------------------------------------------------------------
CSP3_Seq_Rv-Primer(Rev-Comp)  --------------------------------------------------------------ATCGATAGCTGCAATTGGAATTCACCATTAGTCGAAAC
CSP3_cds                      GCCAGTAATCTGTAATTGATCTTATCAATGCCTTAGCAATGCGGAAAAATCCATTCGATTAAATCGATAGCTGCAATTGGAATTCACCATTAGTCGAAAC

                                      410       420       430       440       450       460       470       480       490       500
                              ....|....|....|....|....|....|....|....|....|....|....|....|....|....|....|....|....|....|....|....|
CSP3_Seq_Fw-Primer            ----------------TACTTTTTTTCATCCAGAAGGTGTTGTACTCTCGACAA----------------------------------------------
CSP3_Seq_Rv-Primer(Rev-Comp)  CAAAAGA-------------------------------------------------
CSP3_cds                      CAAAAGAATCCGCATTTACTTTTTTTCATCCAGAAGGTGTTGTACTCTCGACAATG


                              ....|...
CSP3_Seq_Fw-Primer            --------
CSP3_Seq_Rv-Primer(Rev-Comp)
CSP3_cds
```

CSP 5

```
                                      10        20        30        40        50        60        70        80        90       100
                              ....|....|....|....|....|....|....|....|....|....|....|....|....|....|....|....|....|....|....|....|
CSP5_Seq_Fw-Primer            ----------------------------------------------------------------------------------------------------
CSP5_Seq_Rv-Primer(Rev-Comp)  ----------------------------------------------------------------------------------------------------
CSP5_cds                      GCCTGAGATGTCAATTTATTTCTTTATCTACTTAATTGTTCAGTACTGAGCATCAGTTCCATATATGATGAGTAAAATGTATTCTAGAGTAGTTTTTCGA

                                     110       120       130       140       150       160       170       180       190       200
                              ....|....|....|....|....|....|....|....|....|....|....|....|....|....|....|....|....|....|....|....|
CSP5_Seq_Fw-Primer            ----------------------------------------------------------------------------------------------------
CSP5_Seq_Rv-Primer(Rev-Comp)  ----------------------------------------------------------------------------------------------------
CSP5_cds                      AATATTTTAACCCCGGATCTCTATTCCCTCAGCTTTCGCTTGATCTTCGTATTTTCCCCGGTACTTCTTGTCTGCATCGTATTTCTCCATTAATTTTTCC

                                     210       220       230       240       250       260       270       280       290       300
                              ....|....|....|....|....|....|....|....|....|....|....|....|....|....|....|....|....|....|....|....|
CSP5_Seq_Fw-Primer            ----------------------------------------------------------------------------------------------------
CSP5_Seq_Rv-Primer(Rev-Comp)  ----------------------------------------------------------------------------------------------------
CSP5_cds                      CACAGGTCCGGCTTGTTTCGCACTAAAAATTTGATTATTCTCTTTGAACCATCCTTTTGTTTTTGACTGCATTTTGTGCACTCGGTTTCTAAAGCGTCTG

                                     310       320       330       340       350       360       370       380       390       400
                              ....|....|....|....|....|....|....|....|....|....|....|....|....|....|....|....|....|....|....|....|
CSP5_Seq_Fw-Primer            ----------------------------------------------------------------------------------------------------
CSP5_Seq_Rv-Primer(Rev-Comp)  ----------------------------------------------------------------------------------------------------
CSP5_cds                      GAAGGACACTCTTGAGTTCTCTTCCATCAGGAGTACAGTTTCCCTTTTCCAACAAGCAGTTGACGTAATTGTTCAAGATTCGATCATTGTTTAGAATTTG

                                     410       420       430       440       450       460       470       480       490       500
                              ....|....|....|....|....|....|....|....|....|....|....|....|....|....|....|....|....|....|....|....|
CSP5_Seq_Fw-Primer            ------------------------------------------------------------------------------------------------CATG
CSP5_Seq_Rv-Primer(Rev-Comp)  -------------------------------------------------------ACCGCCACCAGACAAGAAAGTAGCACAAACATTGCGATTTTCATG
CSP5_cds                      ATTAATATCAATGTTATCCCATTTTGTGGTATAGCTCTCAGGTCTAGCCGATATTACCGCCACCAGACAAGAAAGTAGCACAAACATTGCGATTTTCATG

                                     510       520       530       540       550       560       570       580       590       600
                              ....|....|....|....|....|....|....|....|....|....|....|....|....|....|....|....|....|....|....|....|
CSP5_Seq_Fw-Primer            ATTGTAACTCGACTGGAATAGCCAAAATCAGAAAATTAAAAACGCGAAAATCTTCTCCAATTGTCGC---------------------------------
CSP5_Seq_Rv-Primer(Rev-Comp)  ATTGTAACTCGACTG-------------------------------------------------------------------------------------
CSP5_cds                      ATTGTAACTCGACTGGAATAGCCAAAATCAGAAAATTAAAAACGCGAAAATCTTCTCCAATTGTCGCACCTCGCAACTCACAATTTCACCTGCGTACAGA

                                     610       620       630       640
                              ....|....|....|....|....|....|....|....|....|....
CSP5_Seq_Fw-Primer            -------------------------------------------------
CSP5_Seq_Rv-Primer(Rev-Comp)  -
CSP5_cds                      T
```

CSP 6

```
                                      10        20        30        40        50        60        70        80        90       100
                              ....|....|....|....|....|....|....|....|....|....|....|....|....|....|....|....|....|....|....|....|
CSP6_Seq_Fw-Primer            ----------------------------------------------------------------------------------------------------
CSP6_Seq_Rv-Primer(Rev-Comp)  ----------------------------------------------------------------------------------------------------
CSP6_cds                      ATTCTTCCAAAAGATTTATTCAAAAAATAAAAAAAACACAAAGAATTTGATACATCATGAGAGCCTCAAAACTCCCGAGTAAGAATTTATAAATGATAAA

                                     110       120       130       140       150       160       170       180       190       200
                              ....|....|....|....|....|....|....|....|....|....|....|....|....|....|....|....|....|....|....|....|
CSP6_Seq_Fw-Primer            ---------------------------------------------TTGGCCTCCCCCTGGAACTTTACCCTGTATTCGTCATGTGGATCGTACTTAGCGG
CSP6_Seq_Rv-Primer(Rev-Comp)  ---------------------------------------------------TCCCCCTGGAACTTTACCCTGTATTCGTCATGTGGATCGTACTTAGCGG
CSP6_cds                      ATTTAATGACAACAATTAGTTATTTAAACCTTAATCCCCCGTGCGTTGGCCTCCCCCTGGAACTTTACCCTGTATTCGTCATGTGGATCGTACTTAGCGG

                                     210       220       230       240       250       260       270       280       290       300
                              ....|....|....|....|....|....|....|....|....|....|....|....|....|....|....|....|....|....|....|....|
CSP6_Seq_Fw-Primer            CGAGTTTC--------------------------------------------------------------------------------------------
CSP6_Seq_Rv-Primer(Rev-Comp)  CGAGTT----------------------------------------------------------------------------------------------
CSP6_cds                      CGAGTTTCTGCCAGACCTTTGGACGCTCGTTTACCAAATACCGGATGACCTTCTCACTGCCTACCCTCTGTTTTTCACTACATTTGTTGCAGCCAGTGGC

                                     310       320       330       340       350       360       370       380       390       400
                              ....|....|....|....|....|....|....|....|....|....|....|....|....|....|....|....|....|....|....|....|
CSP6_Seq_Fw-Primer            ----------------------------------------------------------------------------------------------------
CSP6_Seq_Rv-Primer(Rev-Comp)  ----------------------------------------------------------------------------------------------------
CSP6_cds                      GAGAGCATCGGGGAGAGAAGCTTTCAATTCTTTTCCGTCACTCGTGCAATTTCCCTCGTCCAGAAGACATTTCACATAGTTGTTAAGGAGACGATCATTA

                                     410       420       430       440       450       460       470       480       490       500
                              ....|....|....|....|....|....|....|....|....|....|....|....|....|....|....|....|....|....|....|....|
CSP6_Seq_Fw-Primer            ----------------------------------------------------------------------------------------------------
CSP6_Seq_Rv-Primer(Rev-Comp)  ----------------------------------------------------------------------------------------------------
CSP6_cds                      CGGAGGATCTGGTCCAGGTCTACGTTGTCGTATTTAGTGGTGTAGCCCTGGGGCTTCCCCAGGGTAACAGCTACCACAGCGAGCAATACAATGGCGACCT

                                     510       520       530       540
                              ....|....|....|....|....|....|....|....|..
CSP6_Seq_Fw-Primer            ------------------------------------------
CSP6_Seq_Rv-Primer(Rev-Comp)  ------------------------------------------
CSP6_cds                      TCATTCTCGTAGGTTTGTTAGGAACGAATGTTGTTGAGAGACTGATC
```

CSP 8

```
                            10        20        30        40        50        60        70        80        90       100
                    ....|....|....|....|....|....|....|....|....|....|....|....|....|....|....|....|....|....|....|....|
CSP8_Seq_Fw-Primer  ----------------------------------------------------------------------------------------------------
CSP8_cds            TGTAAAAAAACGAAAATGAAAAAAAAATGTGAAACCATAGTGAACAGTAGGGAATGTAGAAGCGACAGGGAGGGAATACAAAATGGGAAAAGAAAGTCCT

                           110       120       130       140       150       160       170       180       190       200
                    ....|....|....|....|....|....|....|....|....|....|....|....|....|....|....|....|....|....|....|....|
CSP8_Seq_Fw-Primer  ----------------------------------------------------------------------------------------------------
CSP8_cds            GACGAAAATATCACGTGGTATTGTTATCCACGGATGTGGAATGATAGTAACGTCGGCGATATTTTCCATCTGGGTCGTACCTATTCGCAAGCATCTCGAG

                           210       220       230       240       250       260       270       280       290       300
                    ....|....|....|....|....|....|....|....|....|....|....|....|....|....|....|....|....|....|....|....|
CSP8_Seq_Fw-Primer  ----------------------------------------------------------------------------------------------------
CSP8_cds            TTTCTTTGGCATGTATTCCCTCAAATATTTAATTACGTAGATCGCGCCTTTCTTTCTGGCTCTTCGAACATTTGGCACAAGCTGTTTCCAGGGCTTCCGG

                           310       320       330       340       350       360       370       380       390       400
                    ....|....|....|....|....|....|....|....|....|....|....|....|....|....|....|....|....|....|....|....|
CSP8_Seq_Fw-Primer  ----ACCCGTTTCAACTCCTGTCCATCAGGCGGACAAGGCCCCACTCCCATGAGACACCTGAAGTA----------------------------------
CSP8_cds            TAGGACCCGTTTCAACTCCTGTCCATCAGGCGGACAAGGCCCCACTCCCATGAGACACCTGAAGTAATGGTGAAGCAGTCTCTTATTCTGAAGAATTTCA

                           410       420       430       440       450       460       470
                    ....|....|....|....|....|....|....|....|....|....|....|....|....|....|...
CSP8_Seq_Fw-Primer  -------------------------------------------------------------------------
CSP8_cds            TCCAAATTCACTTTATCCCATCTCGTCATGTACGTATTTCTCCTCGGCCATGTGTAACAGTCAATGTCCCTCG
```

CSP 9

```
                                    10        20        30        40        50        60        70        80        90       100
                            ....|....|....|....|....|....|....|....|....|....|....|....|....|....|....|....|....|....|....|....|
CSP9_Seq_Fw-Primer          ----------------------------------------------------------------------------------------------------
CSP9_Seq_Rv-Primer(Rev-Comp) ----------------------------------------------------------------------------------------------------
CSP9_cds                    AACAACAATAACAGTTCTTTATTAGCTGGAGGCTTAATTATTTAAATTATACATTCATGGCTCCAGAATAAAATCCTTCCATTTGTATCAATACCAGTCT

                                   110       120       130       140       150       160       170       180       190       200
                            ....|....|....|....|....|....|....|....|....|....|....|....|....|....|....|....|....|....|....|....|
CSP9_Seq_Fw-Primer          ----------------------------------------------------------------------------------------------------
CSP9_Seq_Rv-Primer(Rev-Comp) ----------------------------------------------------------------------------------------------------
CSP9_cds                    CAGGGAGCAATATCTAATGATTGTTCCGGCCTCATCAATTAATTCCTCCAGGGAACTTACGCCGATCTGGCCATTATATCTCGGACTGCTTTAGCAACAA

                                   210       220       230       240       250       260       270       280       290       300
                            ....|....|....|....|....|....|....|....|....|....|....|....|....|....|....|....|....|....|....|....|
CSP9_Seq_Fw-Primer          ----------------------------------------------------------------------------------------------------
CSP9_Seq_Rv-Primer(Rev-Comp) ----------------------------------------------------------------------------------------------------
CSP9_cds                    TGATTTGATATTTCTCAGGCTCATTTTTTGTATACCATTCGCTTATTTTATCGAAATTGATGGTTTGTCTCTCTGTACATTTTTTGCATCTCGTCACGAT

                                   310       320       330       340       350       360       370       380       390       400
                            ....|....|....|....|....|....|....|....|....|....|....|....|....|....|....|....|....|....|....|....|
CSP9_Seq_Fw-Primer          ----------------------------------------------------------------------------------------------------
CSP9_Seq_Rv-Primer(Rev-Comp) -----------------------------------------------------CAGTACCGGCGAAACATTGATAGTAAGTCTCCCTCTGTCTGTCATTC
CSP9_cds                    CGCCTCTGGAAGAGTGTCCCTGAAGAACCTGGCGGCAGCAGTTTTACAAGGTCCAGTACCGGCGAAACATTGATAGTAAGTCTCCCTCTGTCTGTCATTC

                                   410       420       430       440       450       460       470       480       490       500
                            ....|....|....|....|....|....|....|....|....|....|....|....|....|....|....|....|....|....|....|....|
CSP9_Seq_Fw-Primer          ----GAATTCCGTCTACATCGATGTGATCATATTTGTCGCCATATTTTTCTTCTGCGATAACAGCACTGAGGAAA-------------------------
CSP9_Seq_Rv-Primer(Rev-Comp) TCAAGAATTCCGTCTACATCGATGTGATC-----------------------------------------------------------------------
CSP9_cds                    TCAAGAATTCCGTCTACATCGATGTGATCATATTTGTCGCCATATTTTTCTTCTGCGATAACAGCACTGAGGAAACCAGAACGATGATAACAGCACCTCG

                                   510       520       530       540
                            ....|....|....|....|....|....|....|....|....|....
CSP9_Seq_Fw-Primer          -------------------------------------------------
CSP9_Seq_Rv-Primer(Rev-Comp) -
CSP9_cds                    AAACATTGTTGATGGAGAACGATCTAAGACTCCAAAGAACTTTATCAC
```

OBP 1

```
                                    10        20        30        40        50        60        70        80        90       100
                            ....|....|....|....|....|....|....|....|....|....|....|....|....|....|....|....|....|....|....|....|
OBP1_Seq_Fw-Primer          ----------------------------------------------------------------------------------------------------
OBP1_Seq_Rv-Primer(Rev-Comp) ----------------------------------------------------------------------------------------------------
OBP1_cds                    TTTATCATAGTTTCCAGTTGGCTTTCCACGTGACGGAATTGTTGTTGTGCAAGTCCTCCCCCAGCGATCTTGGGAGTATTCTTGAGCACTTGGAAATAAA

                                   110       120       130       140       150       160       170       180       190       200
                            ....|....|....|....|....|....|....|....|....|....|....|....|....|....|....|....|....|....|....|....|
OBP1_Seq_Fw-Primer          ----------------GAGGATAATTTGGTATTCGTGTCCACTATCGTGAGCATTTGCGTGCACTTGGAATTGATCGACATGGGTATAAAG---------
OBP1_Seq_Rv-Primer(Rev-Comp) ---------------------------------------------------------------------------------------------------A
OBP1_cds                    AGAAAAATTCTGAATCGAGGATAATTTGGTATTCGTGTCCACTATCGTGAGCATTTGCGTGCACTTGGAATTGATCGACATGGGTATAAAGGCGCTTCCA

                                   210       220       230       240       250       260       270       280       290       300
                            ....|....|....|....|....|....|....|....|....|....|....|....|....|....|....|....|....|....|....|....|
OBP1_Seq_Fw-Primer          ----------------------------------------------------------------------------------------------------
OBP1_Seq_Rv-Primer(Rev-Comp) ATAATTCAACGAGAAAACTTTAAACGTCGAAGCACTGGCATCGACATGAGGATCATCGGGGT--------------------------------------
OBP1_cds                    ATAATTCAACGAGAAAACTTTAAACGTCGAAGCACTGGCATCGACATGAGGATCATCGGGGTTTCTCTAGCACTGCTGTCCCTCGTTCTCTTCGTCCGAG

                                   310       320       330       340       350       360       370       380       390       400
                            ....|....|....|....|....|....|....|....|....|....|....|....|....|....|....|....|....|....|....|....|
OBP1_Seq_Fw-Primer          ----------------------------------------------------------------------------------------------------
OBP1_Seq_Rv-Primer(Rev-Comp) ----------------------------------------------------------------------------------------------------
OBP1_cds                    GGGACGACAAGGATCCGCATGGACCTATAAGAGAGAAGTGCAAAGACCAATTCGGTCTTTCCTCTGACGACCTCAAGGCGGCTATGGAGGACCCCAGTGA

                                   410       420       430       440       450       460       470       480       490       500
                            ....|....|....|....|....|....|....|....|....|....|....|....|....|....|....|....|....|....|....|....|
OBP1_Seq_Fw-Primer          ----------------------------------------------------------------------------------------------------
OBP1_Seq_Rv-Primer(Rev-Comp) ----------------------------------------------------------------------------------------------------
OBP1_cds                    CGTTGGATGTTATATCCTGTGCTTCTTCAAGGATCTGTCCATTATGGATGACAGCGGGAAGTTTGATCCCGATGCGGCGCTGGATGCCATCGAGGATAGT

                                   510       520       530       540       550       560       570       580       590       600
                            ....|....|....|....|....|....|....|....|....|....|....|....|....|....|....|....|....|....|....|....|
OBP1_Seq_Fw-Primer          ----------------------------------------------------------------------------------------------------
OBP1_Seq_Rv-Primer(Rev-Comp) ----------------------------------------------------------------------------------------------------
OBP1_cds                    GCCAAGGATGACGCCAAGCCGGTCCTCTCCTCGTGTGATAGTAAAGTAAAAAAATCTACAACTAAAGACCCTTGTGCTCGAGCATTGGAGGTGGTTACTT

                                   610       620       630       640       650       660       670       680       690       700
                            ....|....|....|....|....|....|....|....|....|....|....|....|....|....|....|....|....|....|....|....|
OBP1_Seq_Fw-Primer          ----------------------------------------------------------------------------------------------------
OBP1_Seq_Rv-Primer(Rev-Comp) ----------------------------------------------------------------------------------------------------
OBP1_cds                    GTTTCAAGGAGGAAGCACCTGAACTTTATAAAAATTTGGGAATATTCCATCCACTGGGCTAATAGCACCAGTAAACGACGAACATGGTCCTCATTTTATC

                                   710       720       730       740       750       760       770       780       790
                            ....|....|....|....|....|....|....|....|....|....|....|....|....|....|....|....|....|....|...
OBP1_Seq_Fw-Primer          ------------
OBP1_Seq_Rv-Primer(Rev-Comp) ---------------------------------------------------------------------------------------------
OBP1_cds                    AACAATTTGAAAGTCTACAAATAGACAAAGAATATGGTCATCCAAAATATTAACATGAACGGCGTATTCTCACGCAATATCAATAAATAAAAC
```

OBP 2

```
                                    10        20        30        40        50        60        70        80        90       100
                            ....|....|....|....|....|....|....|....|....|....|....|....|....|....|....|....|....|....|....|....|
OBP2_Seq_Fw-Primer          ----------------------------------------------------------------------------------------------------
OBP2_Seq_Rv-Primer(Rev-Comp) ----------------------------------------------------------------------------------------------------
OBP2_cds                    GTGCTTAACTCTTTTTATTTTCTAATTTCCACAGTAACAACTATCGAGTATTTTTAGAATGAAAATTTTGGTGGTCAGTTGACCTTCTTTGCTTGGAAGC

                                   110       120       130       140       150       160       170       180       190       200
                            ....|....|....|....|....|....|....|....|....|....|....|....|....|....|....|....|....|....|....|....|
OBP2_Seq_Fw-Primer          ----------------------------------------------------------------------------------------------------
OBP2_Seq_Rv-Primer(Rev-Comp) ----------------------------------------------------------------------------------------------------
OBP2_cds                    ATTTCACGTAATTTCCAGCAACTTCACACTCGTTATCACTGATTTCCGCTTCGGGTTTACACATTTTCATTATCTCGATGATTTTCTGGGCCTCCTCTTC

                                   210       220       230       240       250       260       270       280       290       300
                            ....|....|....|....|....|....|....|....|....|....|....|....|....|....|....|....|....|....|....|....|
OBP2_Seq_Fw-Primer          ----------------------------TCGAGCGCTGGGATGTCCATCTTCCCGTCGGTCATAAGGCCACTGTGAGTCATAACACAAGCATGGAAGCAA
OBP2_Seq_Rv-Primer(Rev-Comp) -----------------------------------CTGGGATGTCCATCTTCCCGTCGGTCATAAG----------------------------------
OBP2_cds                    GTCATCAGGATTCCCTGATAGCATTTCCTCGAGCGCTGGGATGTCCATCTTCCCGTCGGTCATAAGGCCACTGTGAGTCATAACACAAGCATGGAAGCAA

                                   310       320       330       340       350       360       370       380       390       400
                            ....|....|....|....|....|....|....|....|....|....|....|....|....|....|....|....|....|....|....|....|
OBP2_Seq_Fw-Primer          CG--------------------------------------------------------------------------------------------------
OBP2_Seq_Rv-Primer(Rev-Comp) ----------------------------------------------------------------------------------------------------
OBP2_cds                    CGGATTTCCTCGTTATCGGCGAACGCGTCTTTCTCCATTAGTTTAACACTTTCTTCACTGAGTTTATCGATGCACGCATCGATGCTCTCCTTTAGTGCCT

                                   410       420       430       440       450       460       470       480       490       500
                            ....|....|....|....|....|....|....|....|....|....|....|....|....|....|....|....|....|....|....|....|
OBP2_Seq_Fw-Primer          ----------------------------------------------------------------------------------------------------
OBP2_Seq_Rv-Primer(Rev-Comp) ----------------------------------------------------------------------------------------------------
OBP2_cds                    TCATGAGTGGGCAATCTTTATCAGGTGGACCTCCAGCAGCGACAGCAAAAGCCCCCACAAGAGCAACGACGAATAGTCCACTCTTCATACTGAATGAATA

                                   510
                            ....|....|....|.
```

```
OBP2_Seq_Fw-Primer              ----------------
OBP2_Seq_Rv-Primer(Rev-Comp)    ----------------
OBP2_cds                        TTGAGCAATGTGTCGT
```

OBP 3

```
                                        10        20        30        40        50        60        70        80        90       100
                                ....|....|....|....|....|....|....|....|....|....|....|....|....|....|....|....|....|....|....|....|
OBP3_Seq_Fw-Primer              ----------------------------------------------------------------------------------------------------
OBP3_Seq_Rv-Primer(Rev-Comp)    ----------------------------------------------------------------------------------------------------
OBP3_cds                        TGGGATATGAATTTTATTAGAGATATTTTCTAAGTTATGTACATGTCGAAAATATTTGACACCAGAAAGTTGTGTATTCTGCAACTGTGATCTATCATTT

                                       110       120       130       140       150       160       170       180       190       200
                                ....|....|....|....|....|....|....|....|....|....|....|....|....|....|....|....|....|....|....|....|
OBP3_Seq_Fw-Primer              -----------------------------------------------------CTAATCCATGGGACCGACGTGTTTCTTGTGCATACACATGCCAATGG
OBP3_Seq_Rv-Primer(Rev-Comp)    -------------------------------------------------------------------CGACGTGTTTCTTGTGCATACACATGCCAATGG
OBP3_cds                        TATATTCAGAGTATTCCACGAGTGACTGTAGGTTACTGTCGTCGTTCAATTCACTAATCCATGGGACCGACGTGTTTCTTGTGCATACACATGCCAATGG

                                       210       220       230       240       250       260       270       280       290       300
                                ....|....|....|....|....|....|....|....|....|....|....|....|....|....|....|....|....|....|....|....|
OBP3_Seq_Fw-Primer              CGTGGGCT--------------------------------------------------------------------------------------------
OBP3_Seq_Rv-Primer(Rev-Comp)    CGTGGGCTG-------------------------------------------------------------------------------------------
OBP3_cds                        CGTGGGCTGTTTCGCAATCATTTGCTCCCTTTTCAGCGTGGCATTCTTCTACTGCGGCCAGAAGGGCATCGTGATCTGGAGCATCACTTGGAACAAGGGA

                                       310       320       330       340       350       360       370       380       390       400
                                ....|....|....|....|....|....|....|....|....|....|....|....|....|....|....|....|....|....|....|....|
OBP3_Seq_Fw-Primer              ----------------------------------------------------------------------------------------------------
OBP3_Seq_Rv-Primer(Rev-Comp)    ----------------------------------------------------------------------------------------------------
OBP3_cds                        TTTTGCCTTGTCTAAATTTACAGTCCCATCCGCCGCCATCACTCCAAGACCTTTCATGATACAGGCACCAAGACATTTTACGTTCTCATCAGCATTAGCT

                                       410       420       430       440       450       460       470       480       490       500
                                ....|....|....|....|....|....|....|....|....|....|....|....|....|....|....|....|....|....|....|....|
OBP3_Seq_Fw-Primer              ----------------------------------------------------------------------------------------------------
OBP3_Seq_Rv-Primer(Rev-Comp)    ----------------------------------------------------------------------------------------------------
OBP3_cds                        CCACCTGTTTCGTGTAGGCTCTTAAGTACACTTGGATCCACACCGGTTTCAATAATGCATTTTTCCACTTGGGCATGCATTTCGGCTTTCATGTCACCCC

                                       510       520       530       540       550       560
                                ....|....|....|....|....|....|....|....|....|....|....|....|....|....
OBP3_Seq_Fw-Primer              ---------------------------------------------------------------------
OBP3_Seq_Rv-Primer(Rev-Comp)    ---------------------------------------------------------------------
OBP3_cds                        CCGAAACGAAAGTGACAGCAAGAGCGCAGAAGACCAAGACGACGGTTGAGGTATTCATGTTGACTTTTC
```

OBP 4

```
                                        10        20        30        40        50        60        70        80        90       100
                                ....|....|....|....|....|....|....|....|....|....|....|....|....|....|....|....|....|....|....|....|
OBP4_Seq_Fw-Primer              ----------------------------------------------------------------------------------------------------
OBP4_Seq_Rv-Primer(Rev-Comp)    ----------------------------------------------------------------------------------------------------
OBP4_cds                        TGATTACAATTGATTTTAATTTTTATGTACATAAATATTGTTTCAATTTTGTTGATTCATCCCAGTCAGTGTTGATATCATCGCGCCTTGCTATTCCGTG

                                       110       120       130       140       150       160       170       180       190       200
                                ....|....|....|....|....|....|....|....|....|....|....|....|....|....|....|....|....|....|....|....|
OBP4_Seq_Fw-Primer              ------------------------------------------------------ACATTTTTATCCGTCTCATAGAAACACTTGATGAACTGCCAGGTCC
OBP4_Seq_Rv-Primer(Rev-Comp)    ----------------------------------------ATGGGAAAAAGCAAACATTTTTATCCGTCTCATAGAAACACTTGATGAACTGCCAGGTCC
OBP4_cds                        GATCGATTACCAGCGACTTCCAGTACACCCCGAGCTTCTCATGGGAAAAAGCAAACATTTTTATCCGTCTCATAGAAACACTTGATGAACTGCCAGGTCC

                                       210       220       230       240       250       260       270       280       290       300
                                ....|....|....|....|....|....|....|....|....|....|....|....|....|....|....|....|....|....|....|....|
OBP4_Seq_Fw-Primer              CTTCGCAACTGTCAGTACTCG-------------------------------------------------------------------------------
OBP4_Seq_Rv-Primer(Rev-Comp)    CTTC------------------------------------------------------------------------------------------------
OBP4_cds                        CTTCGCAACTGTCAGTACTCGTTACTGCAGGAGTACACAAAGCTATGACTTCCTTTAATCGTGCTGTTATGTCATCGGCTGGCAGCATTACGTCCATTTG

                                       310       320       330       340       350       360       370       380       390       400
                                ....|....|....|....|....|....|....|....|....|....|....|....|....|....|....|....|....|....|....|....|
OBP4_Seq_Fw-Primer              ----------------------------------------------------------------------------------------------------
OBP4_Seq_Rv-Primer(Rev-Comp)    ----------------------------------------------------------------------------------------------------
OBP4_cds                        CTTCACCATACTGTCGACATTTGGTAGACCTTCTTTCGTCAAGACTTTGATCATTTTTAACAAACATGCGTAGTAGCACTGCAGAACGGGATCGTCCGGC

                                       410       420       430       440       450       460       470       480       490       500
                                ....|....|....|....|....|....|....|....|....|....|....|....|....|....|....|....|....|....|....|....|
OBP4_Seq_Fw-Primer              ----------------------------------------------------------------------------------------------------
OBP4_Seq_Rv-Primer(Rev-Comp)    ----------------------------------------------------------------------------------------------------
OBP4_cds                        ATCGGGCCGGTTTTCGGTGCGTCAACCAACTCTGCACTCGCACCAGTTTTTTGCATGCAGGTCTTCCTCATTGGCATCAGCATGTTTGTTACTTGAGGTA

                                       510       520       530       540       550       560       570       580       590       600
                                ....|....|....|....|....|....|....|....|....|....|....|....|....|....|....|....|....|....|....|....|
OBP4_Seq_Fw-Primer              ----------------------------------------------------------------------------------------------------
OBP4_Seq_Rv-Primer(Rev-Comp)    ----------------------------------------------------------------------------------------------------
OBP4_cds                        TCGTCATTTTAGCCCCGACATTGTCAAAATTCAGAACAACCAGAACTATCACTAATCCAGAAATAAAAGCACTTTTCATTTTTGAATTTTGATTCTTCGA

                                       610       620       630       640
                                ....|....|....|....|....|....|....|....|....|
OBP4_Seq_Fw-Primer              ---------------------------------------------
OBP4_Seq_Rv-Primer(Rev-Comp)    ---------------------------------------------
OBP4_cds                        TTGAGAATCCAAGAAAAAAAATTCTAGATGTATCGAATCACTGAA
```

OBP 5

```
                                        10        20        30        40        50        60        70        80        90       100
                                ....|....|....|....|....|....|....|....|....|....|....|....|....|....|....|....|....|....|....|....|
OBP5_Seq_Fw-Primer              ----------------------------------------------------------------------------------------------------
OBP5_Seq_Rv-Primer(Rev-Comp)    ----------------------------------------------------------------------------------------------------
OBP5_cds                        AAATTAATCTGAGGTAATTACTGCCACAGATGTACGAAATGATTGACATTTTTTTCAAATGTGCGCTTAGAAATTGATTTATTTGGATGTAATTACACAT

                                       110       120       130       140       150       160       170       180       190       200
                                ....|....|....|....|....|....|....|....|....|....|....|....|....|....|....|....|....|....|....|....|
OBP5_Seq_Fw-Primer              ----------------------------------------------------------------------------------------------------
OBP5_Seq_Rv-Primer(Rev-Comp)    ----------------------------------------------------------------------------------------------------
OBP5_cds                        TCATTATTGCAAATTCATAATTGGTTCTTAATTCGTCATAGTAACACCATTGGTAACTGTAGGATAGTTGTTTCTTGCCGTTGTTACATGATGAAATATT

                                       210       220       230       240       250       260       270       280       290       300
                                ....|....|....|....|....|....|....|....|....|....|....|....|....|....|....|....|....|....|....|....|
OBP5_Seq_Fw-Primer              ----------------------------------------------------------------------------------------------------
OBP5_Seq_Rv-Primer(Rev-Comp)    ----------------------------------------------------------------------------------------------------
OBP5_cds                        GGTCAGGGAAAGTATTGAAGAAACATTTAGACATGTGATAAGAGAGATCGCATTGATCTCCTGCTGCGTTCATCTTGATATCCTTGCAAGTCACTCCGAG

                                       310       320       330       340       350       360       370       380       390       400
                                ....|....|....|....|....|....|....|....|....|....|....|....|....|....|....|....|....|....|....|....|
OBP5_Seq_Fw-Primer              ----------------------------------------------------------------------------------------------------
OBP5_Seq_Rv-Primer(Rev-Comp)    ----------------------------------------------------------------------------------------------------
OBP5_cds                        TTGTTTTGCCATGTCCTTCATATCTGGGGGAGCGACTTTAACCAGTTGTTCAAAGTTCAGTCCCTTTTTCGAGATCAGTCCAAATTGTGCAAATAAGCAC

                                       410       420       430       440       450       460       470       480       490       500
                                ....|....|....|....|....|....|....|....|....|....|....|....|....|....|....|....|....|....|....|....|
OBP5_Seq_Fw-Primer              ----------------------------------------------CAGCTCTTTCAATTAGGTCAACAGTGGTACCCGTCTCTTCAATGCAAACCTTGC
OBP5_Seq_Rv-Primer(Rev-Comp)    ---------------------------------------------------------------------GTGGTACCCGTCTCTTCAATGCAAACCTTGC
OBP5_cds                        TTGAGATAACATTTCAATTTGTCGTCCTCAGCAAAATCGCCCTTTCCAGCTCTTTCAATTAGGTCAACAGTGGTACCCGTCTCTTCAATGCAAACCTTGC

                                       510       520       530       540       550       560       570       580       590       600
                                ....|....|....|....|....|....|....|....|....|....|....|....|....|....|....|....|....|....|....|....|
OBP5_Seq_Fw-Primer              GAATGTCTCCAGCAACTTCTTGAAATTCTTTCGGAATGGGCCCAGTTGAAATGTAGCTCAGCAGGCAGAAC-----------------------------
OBP5_Seq_Rv-Primer(Rev-Comp)    GAATGTCTCCAGCAACTTCTTGAAA---------------------------------------------------------------------------
OBP5_cds                        GAATGTCTCCAGCAACTTCTTGAAATTCTTTCGGAATGGGCCCAGTTGAAATGTAGCTCAGCAGGCAGAACAAAATGATTGCACTTAGTATATGCTTAGC
```

```
                                     610       620       630       640
                              ....|....|....|....|....|....|....|....|....|
OBP5_Seq_Fw-Primer            ---------------------------------------------
OBP5_Seq_Rv-Primer(Rev-Comp)  ---------------------------------------------
OBP5_cds                      CATGTTGGTTCAATTTGAAAATGAGGAATTAATACTTGAACTGCC
```

OBP 6

```
                                      10        20        30        40        50        60        70        80        90       100
                              ....|....|....|....|....|....|....|....|....|....|....|....|....|....|....|....|....|....|....|....|
OBP6_Seq_Fw-Primer            ----------------------------------------------------------------------------------------------------
OBP6_Seq_Rv-Primer(Rev-Comp)  ----------------------------------------------------------------------------------------------------
OBP6_cds                      CGTTTGCATATTTGTATTTTATTGGAATAATTCTGTCTATTGATTCATCACTGATTAAGTATCTTAATCCTTCAGCATATCGCCTTAATAACTGCTCTAA

                                     110       120       130       140       150       160       170       180       190       200
                              ....|....|....|....|....|....|....|....|....|....|....|....|....|....|....|....|....|....|....|....|
OBP6_Seq_Fw-Primer            ----------------------------------------------------------------------------------------------------
OBP6_Seq_Rv-Primer(Rev-Comp)  ----------------------------------------------------------------------------------------------------
OBP6_cds                      TAAATTTTCGTTGCTTATCACATGTATTACGTATTCAGGCATCGAATGACACGCACACCCATTACAATCTGTCAGGATTGGTTATCGATCCAAGGTCGGA

                                     210       220       230       240       250       260       270       280       290       300
                              ....|....|....|....|....|....|....|....|....|....|....|....|....|....|....|....|....|....|....|....|
OBP6_Seq_Fw-Primer            ----------------------------------------------------------------------------------------------------
OBP6_Seq_Rv-Primer(Rev-Comp)  ----------------------------------------------------------------------------------------------------
OBP6_cds                      GGAATTGAAGGTCAATGACTGGTGAAAATTTCCGGCTGAAGTATCTTGTCTTCTTCTTCATATTCTTTTGTACTACTTTCCTGCTATTCTCCAACTGCTG

                                     310       320       330       340       350       360       370       380       390       400
                              ....|....|....|....|....|....|....|....|....|....|....|....|....|....|....|....|....|....|....|....|
OBP6_Seq_Fw-Primer            ----------------------------------------------------------------------------------------------------
OBP6_Seq_Rv-Primer(Rev-Comp)  ----------------------------------------------------------------------------------------------------
OBP6_cds                      ACATTTTCAATTAACTGATTACACTCGTTCATCTGCAAACTCGCCCAGCCTTCTTGATTTTTCTCCGTTGACCAGCTCAGATCATGAACCACAGATCTGG

                                     410       420       430       440       450       460       470       480       490       500
                              ....|....|....|....|....|....|....|....|....|....|....|....|....|....|....|....|....|....|....|....|
OBP6_Seq_Fw-Primer            ------------------------------------------------------------------------------------------------ACCT
OBP6_Seq_Rv-Primer(Rev-Comp)  -------------------------------------------------------------TGTTTGGCGCAAGTCTCCAGAAGATCTCTAGCTGCACCT
OBP6_cds                      TCGTTTTGCTTGTACACATTTAGCAATCTCGAAGACTTTGTCACATGGATCCGCTCCAGGTTGTTTGGCGCAAGTCTCCAGAAGATCTCTAGCTGCACCT

                                     510       520       530       540       550       560       570       580       590       600
                              ....|....|....|....|....|....|....|....|....|....|....|....|....|....|....|....|....|....|....|....|
OBP6_Seq_Fw-Primer            GCATGTGATCGGGGAGGAATCCTACCAACATATCTACTTCAAGCTCACCCTCGTCAATG-----------------------------------------
OBP6_Seq_Rv-Primer(Rev-Comp)  GCATGTG---------------------------------------------------------------------------------------------
OBP6_cds                      GCATGTGATCGGGGAGGAATCCTACCAACATATCTACTTCAAGCTCACCCTCGTCAATGACTCCGAAAGCTGCGAACAGGCAGTCCATGTAGCAGGTCAA

                                     610       620       630       640       650       660       670       680       690       700
                              ....|....|....|....|....|....|....|....|....|....|....|....|....|....|....|....|....|....|....|....|
OBP6_Seq_Fw-Primer            ----------------------------------------------------------------------------------------------------
OBP6_Seq_Rv-Primer(Rev-Comp)  ----------------------------------------------------------------------------------------------------
OBP6_cds                      AGCTCGATTGTTGGGGAGGGCTCCAGTGTTGACTTCATCGATAAGAGCTTCAGTGGTTCCATGTTCACTCATGCATCGCGCCTTATCATCCGCAACCATT

                                     710       720       730       740       750       760       770       780       790       800
                              ....|....|....|....|....|....|....|....|....|....|....|....|....|....|....|....|....|....|....|....|
OBP6_Seq_Fw-Primer            ----------------------------------------------------------------------------------------------------
OBP6_Seq_Rv-Primer(Rev-Comp)  ----------------------------------------------------------------------------------------------------
OBP6_cds                      GCCATCATGTCATCGGTAATGAAATCCGGGCGCCCTGCAGACACAAGGAGAGCTTGCATAGCCATTCCAATGAGAAAACAACAGACCATATGTCTCGCCA

                                     810       820       830       840       850       860       870       880       890       900
                              ....|....|....|....|....|....|....|....|....|....|....|....|....|....|....|....|....|....|....|....|
OBP6_Seq_Fw-Primer            ----------------------------------------------------------------------------------------------------
OBP6_Seq_Rv-Primer(Rev-Comp)  ----------------------------------------------------------------------------------------------------
OBP6_cds                      TGTTGTACCTCGATGATGTTTTACACAGTCAACTTAAATATTTATAGAATCAATAGTATCGATAAATAATCGAACACAGTGAGTAGATGCGTTGTTCTTC

                                     910       920       930       940
                              ....|....|....|....|....|....|....|....|....|..
OBP6_Seq_Fw-Primer            -----------------------------------------------
OBP6_Seq_Rv-Primer(Rev-Comp)  -
OBP6_cds                      G
```

OBP 7

```
                                      10        20        30        40        50        60        70        80        90       100
                              ....|....|....|....|....|....|....|....|....|....|....|....|....|....|....|....|....|....|....|....|
OBP7_Seq_Fw-Primer            ----------------------------------------------------------------------------------------------------
OBP7_Seq_Rv-Primer(Rev-Comp)  ----------------------------------------------------------------------------------------------------
OBP7_cds                      GTTTATGAAGAATTTTATGTTCGTAAAAACTGATGCCGCGATTAACGTATCAAGCATTTCGCGGATCTACGATAAATCATTTGTCATAATTAAACGATCT

                                     110       120       130       140       150       160       170       180       190       200
                              ....|....|....|....|....|....|....|....|....|....|....|....|....|....|....|....|....|....|....|....|
OBP7_Seq_Fw-Primer            ----------------------------------------------------------------------------TTGGTCGCATCCTTTCACCTCATC
OBP7_Seq_Rv-Primer(Rev-Comp)  --------------------------------------------TCCTTCCAAAGGCACTTGTGAAACATCGCCGATTGGTCGCATCCTTTCACCTCATC
OBP7_cds                      CGATACAGTGGTTTGCATCCTTAGCTGCTTGCACATAGTCCTGGTCCTTCCAAAGGCACTTGTGAAACATCGCCGATTGGTCGCATCCTTTCACCTCATC

                                     210       220       230       240       250       260       270       280       290       300
                              ....|....|....|....|....|....|....|....|....|....|....|....|....|....|....|....|....|....|....|....|
OBP7_Seq_Fw-Primer            ACCAATTTCTGAGAAGCAACGAGAGGCTGC----------------------------------------------------------------------
OBP7_Seq_Rv-Primer(Rev-Comp)  ACCAA-----------------------------------------------------------------------------------------------
OBP7_cds                      ACCAATTTCTGAGAAGCAACGAGAGGCTGCTTTGTCTAAAGCTTCCTGCAATGGCGCTCCGTCAACCAATTTTCGGAAAACGTTGCGGATTATATCCTCC

                                     310       320       330       340       350       360       370       380       390       400
                              ....|....|....|....|....|....|....|....|....|....|....|....|....|....|....|....|....|....|....|....|
OBP7_Seq_Fw-Primer            ----------------------------------------------------------------------------------------------------
OBP7_Seq_Rv-Primer(Rev-Comp)  ----------------------------------------------------------------------------------------------------
OBP7_cds                      GAAAGTTCTCCAGCGTCATTCAACACGTGGATTTCGTCATGCGTGCACCGCCAAAAGCATTCATCGTCCATATCCATCTCCTTTTGACATTCTTCTTTAG

                                     410       420       430       440       450       460       470       480       490       500
                              ....|....|....|....|....|....|....|....|....|....|....|....|....|....|....|....|....|....|....|....|
OBP7_Seq_Fw-Primer            ----------------------------------------------------------------------------------------------------
OBP7_Seq_Rv-Primer(Rev-Comp)  ----------------------------------------------------------------------------------------------------
OBP7_cds                      CGTCCCGCCATTCTTGACTGACCGTGCAGTCGTCCGGAGAACAACTCACTCCACCCACCACCAAAATTAATAAAAGCTGATGAATGCCGCTCATTTTTAA

                                     510       520
                              ....|....|....|....|....|
OBP7_Seq_Fw-Primer            -------------------------
OBP7_Seq_Rv-Primer(Rev-Comp)  -------------------------
OBP7_cds                      CGTCAATATGTCAATCGAGGAGTCG
```

OBP 8

```
                                      10        20        30        40        50        60        70        80        90       100
                              ....|....|....|....|....|....|....|....|....|....|....|....|....|....|....|....|....|....|....|....|
OBP8_Seq_Fw-Primer            ----------------------------------------------------------------------------------------------------
OBP8_Seq_Rv-Primer(Rev-Comp)  ----------------------------------------------------------------------------------------------------
OBP8_cds                      GGTCGTTTCTGCGGCTTGAAAATCAGAGGGCTGATACATGGACTGTCAAAGACATCAAACGTGATAGCCCTCTTGGCATTGGGCTTCCGTACAGACGTTA

                                     110       120       130       140       150       160       170       180       190       200
                              ....|....|....|....|....|....|....|....|....|....|....|....|....|....|....|....|....|....|....|....|
OBP8_Seq_Fw-Primer            ----------------------------------------------------------------------------------------------------
OBP8_Seq_Rv-Primer(Rev-Comp)  ----------------------------------------------------------------------------------------------------
OBP8_cds                      AATCGTGGGGAGTCTTTAGTGGACTCGGCCCAGCCGCTAAAAAACTGTCTTCCAGAGCCATTGATGCTGGAGAAAATAACACTGATGTGTCTATACTTTT

                                     210       220       230       240       250       260       270       280       290       300
                              ....|....|....|....|....|....|....|....|....|....|....|....|....|....|....|....|....|....|....|....|
```

```
OBP8_Seq_Fw-Primer           ----------------------------------------------------------------------------------------------------
OBP8_Seq_Rv-Primer(Rev-Comp) ----------------------------------------------------------------------------------------------------
OBP8_cds                     ACTCGGAGTTTCGACAGCAAAACTCATGTCAGAGCTGTCTGTTGAGGTCGCCCATGCTTGACAAAGGGCTTTGCGTGCCCTTTTCCCCCCAGGTGCACTA

                                    310       320       330       340       350       360       370       380       390       400
                             ....|....|....|....|....|....|....|....|....|....|....|....|....|....|....|....|....|....|....|....|
OBP8_Seq_Fw-Primer           ----------------------------------------------------------------------------------------------------
OBP8_Seq_Rv-Primer(Rev-Comp) ----------------------------------------------------------------------------------------------------
OBP8_cds                     TTTTCCTTTCCACGTCGTCGTCCAGTGCCGTAACCGGAGTCCTGGAGCATACTGCTGCTGGCCAAACTCTCACGATCAGTTTCCTGCTTGATAATTTCGG

                                    410       420       430       440       450       460       470       480       490       500
                             ....|....|....|....|....|....|....|....|....|....|....|....|....|....|....|....|....|....|....|....|
OBP8_Seq_Fw-Primer           ----------------------------------------------------------------------------------------------------
OBP8_Seq_Rv-Primer(Rev-Comp) ----------------------------------------------------------------------------------------------------
OBP8_cds                     TGAGTGCATCCAGACGACTTGGGGTGGCTGGCAATTGCGTTGCAGCTCCGCTGCGTCTTTCAATCTCAGCAAGTGCATCCTTGAAAGGAGTGGGAGTCCT

                                    510       520       530       540       550       560       570       580       590       600
                             ....|....|....|....|....|....|....|....|....|....|....|....|....|....|....|....|....|....|....|....|
OBP8_Seq_Fw-Primer           ----------------------------------------------------------------------------------------------------
OBP8_Seq_Rv-Primer(Rev-Comp) ----------------------------------------------------------------------------------------------------
OBP8_cds                     CGGAGTGTTGCCATCGTTGTGTCCACGACGAAGTCCAGAGGGTTGAGGAGTAGTAAGGGTCCCTGGGGAGCCCTTGGGTGTGCTGGCACGTATCCATGAC

                                    610       620       630       640       650       660       670       680       690       700
                             ....|....|....|....|....|....|....|....|....|....|....|....|....|....|....|....|....|....|....|....|
OBP8_Seq_Fw-Primer           ----------------------------------------------------------------------------------------------------
OBP8_Seq_Rv-Primer(Rev-Comp) ----------------------------------------------------------------------------------------------------
OBP8_cds                     GATTCAGGGCTCGCTAAACTGTTGAGGAATTGCGAGGGGCTGAAGGGAAGTTGCTTTATAGGGGTCGAGGGGGCTGTCGTTGCCTCCTGGGAGGATGTCC

                                    710       720       730       740       750       760       770       780       790       800
                             ....|....|....|....|....|....|....|....|....|....|....|....|....|....|....|....|....|....|....|....|
OBP8_Seq_Fw-Primer           ----------------------------------------------------------------------------------------------------
OBP8_Seq_Rv-Primer(Rev-Comp) ----------------------------------------------------------------------------------------------------
OBP8_cds                     CCATGAGGAAGTCCTGGTGACCACTGTCCGGAGTCTCGGTACGGGGGAGAATGTGCTGGAAGCCTCGACGTTTACGGAGAATAGAGGGCGGACTTAGTCT

                                    810       820       830       840       850       860       870       880       890       900
                             ....|....|....|....|....|....|....|....|....|....|....|....|....|....|....|....|....|....|....|....|
OBP8_Seq_Fw-Primer           ----------------------------------------------------------------------------------------------------
OBP8_Seq_Rv-Primer(Rev-Comp) ----------------------------------------------------------------------------------------------------
OBP8_cds                     GCCATCTTCGTCGTCTTCGAGGTCTGGCATTGGCAGGAGACGAATGTCTGAGGTATTCGACGAGGCTTCTGCGCCCATGTTCAGATTGAAGTATTTGGAG

                                    910       920       930       940       950       960       970       980       990      1000
                             ....|....|....|....|....|....|....|....|....|....|....|....|....|....|....|....|....|....|....|....|
OBP8_Seq_Fw-Primer           ----------------------------------------------------------------------------------------------------
OBP8_Seq_Rv-Primer(Rev-Comp) ----------------------------------------------------------------------------------------------------
OBP8_cds                     AACGGTGAAATAATTGGTTTTCTATCGTTGATAGGTTCGTCGGGCCTCTCGTCCATCTGTATAATACTCTGTAGATAATTCGTTTGAGATTGTTGCTGTA

                                   1010      1020      1030      1040      1050      1060      1070      1080      1090      1100
                             ....|....|....|....|....|....|....|....|....|....|....|....|....|....|....|....|....|....|....|....|
OBP8_Seq_Fw-Primer           ----------------------------------------------------------------------------------------------------
OBP8_Seq_Rv-Primer(Rev-Comp) ----------------------------------------------------------------------------------------------------
OBP8_cds                     TCATAGAATGCTGGGGCGTTTGTCCCCAGGCGCCAGTCCAGTCTAGTGAATCAATCTCTAAACTCCCTGAGGTCACAGTTGCCCGACTATCCTCGGCCTT

                                   1110      1120      1130      1140      1150      1160      1170      1180      1190      1200
                             ....|....|....|....|....|....|....|....|....|....|....|....|....|....|....|....|....|....|....|....|
OBP8_Seq_Fw-Primer           ----------------------------------------------------------------------------------------------------
OBP8_Seq_Rv-Primer(Rev-Comp) ----------------------------------------------------------------------------------------------------
OBP8_cds                     GATCTCAACCTTCACGGATTGTTCCTGGCACGAGGATTCCTCCGCTGTTGATACTTAGACGATCACTAATTGACTCATATCCAGTAAATATGGTTAAATA

                                   1210      1220      1230      1240      1250      1260      1270      1280      1290      1300
                             ....|....|....|....|....|....|....|....|....|....|....|....|....|....|....|....|....|....|....|....|
OBP8_Seq_Fw-Primer           ----------------------------------------------------------------------------------------------------
OBP8_Seq_Rv-Primer(Rev-Comp) ----------------------------------------------------------------------------------------------------
OBP8_cds                     TATTGTCAGTGCTTTCATCGCGGTGTGTCTGGTGGCTGCCATCAGGGCTGGTGAGATACCCCCGGAATTCAAAGAGATCGCACCCGAAGTGAGAAGAGTA

                                   1310      1320      1330      1340      1350      1360      1370      1380      1390      1400
                             ....|....|....|....|....|....|....|....|....|....|....|....|....|....|....|....|....|....|....|....|
OBP8_Seq_Fw-Primer           ----------------------------------------------------------------------------------------------------
OBP8_Seq_Rv-Primer(Rev-Comp) ----------------------------------------------------------------------------------------------------
OBP8_cds                     TGTCTAGAAGAAAGTGGCGCTGAAAATGAATGGGTTGTGAAAGCAAATAAGGGCGATTTCACTGACGATCCGAAATTCAAGTGCTACCTGAAGTGCACCC

                                   1410      1420      1430      1440      1450      1460      1470      1480      1490      1500
                             ....|....|....|....|....|....|....|....|....|....|....|....|....|....|....|....|....|....|....|....|
OBP8_Seq_Fw-Primer           ------------------------------------------------------------------------------------TCGATAAAGTCATCTC
OBP8_Seq_Rv-Primer(Rev-Comp) -------------------------------------------TGCACTGACTAAACTGGCACCACCTGCTTACAAAGA---------------------
OBP8_cds                     TGGCTCAGTTTGGTGCTGTTTCGAGGAAGGGAGTCAATTTCGATGCACTGACTAAACTGGCACCACCTGCTTACAAAGAAATCCTCGATAAAGTCATCTC

                                   1510      1520      1530      1540      1550      1560      1570      1580      1590      1600
                             ....|....|....|....|....|....|....|....|....|....|....|....|....|....|....|....|....|....|....|....|
OBP8_Seq_Fw-Primer           CGCGTGTAAGGACACGAAGCCAACAATACCCGGAGA----------------------------------------------------------------
OBP8_Seq_Rv-Primer(Rev-Comp) ----------------------------------------------------------------------------------------------------
OBP8_cds                     CGCGTGTAAGGACACGAAGCCAACAATACCCGGAGATGTATGCGATCAAGTGTACGAGGCTAGCAAATGCTTCTACCGAGCAGCTCCTGACAATTACTTC

                                   1610      1620      1630      1640      1650      1660      1670      1680      1690      1700
                             ....|....|....|....|....|....|....|....|....|....|....|....|....|....|....|....|....|....|....|....|
OBP8_Seq_Fw-Primer           ----------------------------------------------------------------------------------------------------
OBP8_Seq_Rv-Primer(Rev-Comp) ----------------------------------------------------------------------------------------------------
OBP8_cds                     GTTATGTAAGTGCCTAATGGAGTGCAACCCTGCCACTAACCGAGAATGATAAAGACATTGGAAAGGAAAATGATAGAGAGTTTAAGTAGAATTCGGTAGG

                                   1710      1720      1730      1740      1750      1760      1770      1780      1790      1800
                             ....|....|....|....|....|....|....|....|....|....|....|....|....|....|....|....|....|....|....|....|
OBP8_Seq_Fw-Primer           ----------------------------------------------------------------------------------------------------
OBP8_Seq_Rv-Primer(Rev-Comp) ----------------------------------------------------------------------------------------------------
OBP8_cds                     GGGAATTCATTAATTTTGATCTCCATAATTATATGTTTGTAATTATTTGCGATACAATACATGAGCTACAAGGACAACTTGTACATCAAAGAGGGGTTGT

                                   1810      1820      1830      1840      1850      1860      1870      1880      1890      1900
                             ....|....|....|....|....|....|....|....|....|....|....|....|....|....|....|....|....|....|....|....|
OBP8_Seq_Fw-Primer           ----------------------------------------------------------------------------------------------------
OBP8_Seq_Rv-Primer(Rev-Comp) ----------------------------------------------------------------------------------------------------
OBP8_cds                     TTGTTATTCCGGGACTGAAATCTAATTTTATTGTTTAAATGTCGAAGTACCGAGAGATATTCAAAACCTCATCGTAAGTGATACTCGGTCTCATCAAAAA

                                   1910      1920      1930      1940      1950      1960      1970      1980      1990      2000
                             ....|....|....|....|....|....|....|....|....|....|....|....|....|....|....|....|....|....|....|....|
OBP8_Seq_Fw-Primer           ----------------------------------------------------------------------------------------------------
OBP8_Seq_Rv-Primer(Rev-Comp) ----------------------------------------------------------------------------------------------------
OBP8_cds                     AGTGTTTTTTTACTTATTTTAAGTAAAAATTGATGAAGCACTAACAGAGATAATAACGAGGGTTGCTGATGCTTATGGAGTGACATCAACCGCAAAATTT

                                   2010      2020      2030      2040      2050      2060      2070      2080      2090      2100
                             ....|....|....|....|....|....|....|....|....|....|....|....|....|....|....|....|....|....|....|....|
OBP8_Seq_Fw-Primer           ----------------------------------------------------------------------------------------------------
OBP8_Seq_Rv-Primer(Rev-Comp) ----------------------------------------------------------------------------------------------------
OBP8_cds                     CGTAGTGGTGGCGCATGAACTAGGATGGTGGACGTGCCTGTCGGTAAGGCCTCACCACGGAGTACGGGGTACTCAATTTGTGAGACGATTTGCGATTTTT

                                   2110      2120      2130      2140      2150      2160      2170      2180      2190      2200
                             ....|....|....|....|....|....|....|....|....|....|....|....|....|....|....|....|....|....|....|....|
OBP8_Seq_Fw-Primer           ----------------------------------------------------------------------------------------------------
OBP8_Seq_Rv-Primer(Rev-Comp) ----------------------------------------------------------------------------------------------------
OBP8_cds                     CTACAGCTCCCGAAATATGTTAATATCCGTTTTTACGTCTTTCGCATTTTTCTCGGCTTCTGGATCTCCCTATATCATACCTTACTCACGGTATCTGTAA

                                   2210      2220      2230      2240      2250      2260      2270      2280      2290      2300
                             ....|....|....|....|....|....|....|....|....|....|....|....|....|....|....|....|....|....|....|....|
OBP8_Seq_Fw-Primer           ----------------------------------------------------------------------------------------------------
OBP8_Seq_Rv-Primer(Rev-Comp) ----------------------------------------------------------------------------------------------------
OBP8_cds                     GTGACGACCTCTGTGCACCACAACTGGAACACACGGCGCCGCGGATGACGTTCCGAGGTCCTTGTCCACTGCTTTGGCAGTGACATAATCGTCATGTGTC
```

```
                                          2310      2320      2330      2340      2350      2360      2370      2380      2390      2400
                                  ....|....|....|....|....|....|....|....|....|....|....|....|....|....|....|....|....|....|....|....|
OBP8_Seq_Fw-Primer                ----------------------------------------------------------------------------------------------------
OBP8_Seq_Rv-Primer(Rev-Comp)      -----------------------------------------------------------------
OBP8_cds                          ATCTGTATCTAATTATAATATTATTCTCACTGTATACTTAATAATTAAACAATCCTCTCAATACC

                                          2410      2420
                                  ....|....|....|....|..
OBP8_Seq_Fw-Primer                ----------------------
OBP8_Seq_Rv-Primer(Rev-Comp)
OBP8_cds
```

OBP 9

```
                                            10        20        30        40        50        60        70        80        90       100
                                  ....|....|....|....|....|....|....|....|....|....|....|....|....|....|....|....|....|....|....|....|
OBP9_Seq_Fw-Primer                ----------------------------------------------------------------------------------------------------
OBP9_Seq_Rv-Primer(Rev-Comp)      ----------------------------------------------------------------------------------------------------
OBP9_cds                          TATTCATAATTTTTTGTCCACTACATTTTATTTATTTCATTCATTTAATTATTTTCATTGTAAATTAATTGATTCATTTGAATAATTCAACCATAAAAAA

                                           110       120       130       140       150       160       170       180       190       200
                                  ....|....|....|....|....|....|....|....|....|....|....|....|....|....|....|....|....|....|....|....|
OBP9_Seq_Fw-Primer                ----------------------------------------------------------------------------------------------------
OBP9_Seq_Rv-Primer(Rev-Comp)      ----------------------------------------------------------------------------------------------------
OBP9_cds                          TCCCCTTCATATTTTTTTCCTCAATTGGACCAATCAGTTGGGAGACTCACAAAACTGCCGTTCTCTCATTTCAATTTATTAATGCTTGTTTATTTAATGG

                                           210       220       230       240       250       260       270       280       290       300
                                  ....|....|....|....|....|....|....|....|....|....|....|....|....|....|....|....|....|....|....|....|
OBP9_Seq_Fw-Primer                ----------------------------------------------------------------------------------------------------
OBP9_Seq_Rv-Primer(Rev-Comp)      ----------------------------------------------------------------------------------------------------
OBP9_cds                          CTTTTTATTCCAATAAATCAAATCGTACGATAAAGCCCCGATAAATAATGTTTCGAAACATGGTGGGAATTTAGTCACAAAAGCAGTTCATATGCATTGG

                                           310       320       330       340       350       360       370       380       390       400
                                  ....|....|....|....|....|....|....|....|....|....|....|....|....|....|....|....|....|....|....|....|
OBP9_Seq_Fw-Primer                ----------------------------------------------------------------------------------------------------
OBP9_Seq_Rv-Primer(Rev-Comp)      ----------------------------------------------------------------------------------------------------
OBP9_cds                          CCGATAATCAACAAAATCATAGGGATCATCATTGTAGTAACACAGGATTATTTTTTACATGAGGTGTGTTTTCTGCTCATTATAACACTTCATTATCCTT

                                           410       420       430       440       450       460       470       480       490       500
                                  ....|....|....|....|....|....|....|....|....|....|....|....|....|....|....|....|....|....|....|....|
OBP9_Seq_Fw-Primer                ----------------------------------------------------------------------------------------------------
OBP9_Seq_Rv-Primer(Rev-Comp)      ----------------------------------------------------------------------------------------------------
OBP9_cds                          TGCGCAGTGCCACAGGCGTTGGAACCCTTCAGATCTTTGCACTTGGCCATGATATCATTGATCTGCTCAGCGGGCAATTTACGGGCCAACGTGTCGCGTG

                                           510       520       530       540       550       560       570       580       590       600
                                  ....|....|....|....|....|....|....|....|....|....|....|....|....|....|....|....|....|....|....|....|
OBP9_Seq_Fw-Primer                ----------------------------------------------------------------------------------------------------
OBP9_Seq_Rv-Primer(Rev-Comp)      ----------------------------------------------------------------------------------------------------
OBP9_cds                          TGACGATTTCATCGAGCACACCTTCATCATTCATCAGATTCCCTTTCTTCAGCATACAAAGGGTGTAACAACGAAGGTTCTCTTTATCAGCCCATTCGCC

                                           610       620       630       640       650       660       670       680       690       700
                                  ....|....|....|....|....|....|....|....|....|....|....|....|....|....|....|....|....|....|....|....|
OBP9_Seq_Fw-Primer                ----------------------------------------------------------------------------------------------TGCTCG
OBP9_Seq_Rv-Primer(Rev-Comp)      ----------------------------------------------CGGCAAGCATCTCTGTATCGCTGCCGTCTCGCCTCCGGGGTTTCTCTCTGCTC-
OBP9_cds                          TTTGCGAGCCTTATCAATGTCAGCTTGATCAGCCCCTGTCTCCGCACGGCAAGCATCTCTGTATCGCTGCCGTCTCGCCTCCGGGGTTTCTCTCTGCTCG

                                           710       720       730       740       750       760       770       780       790       800
                                  ....|....|....|....|....|....|....|....|....|....|....|....|....|....|....|....|....|....|....|....|
OBP9_Seq_Fw-Primer                GATGCCAGAGCACCAACGATGCAGACGAGGACGGCAATCACCAGGAATTA--------------------------------------------------
OBP9_Seq_Rv-Primer(Rev-Comp)      ----------------------------------------------------------------------------------------------------
OBP9_cds                          GATGCCAGAGCACCAACGATGCAGACGAGGACGGCAATCACCAGGAATTACATGATGAGTTGTTGGATTATTCTGAGCTCCCGAAAGTTAGAACCAGTTG

                                           810       820       830       840       850
                                  ....|....|....|....|....|....|....|....|....|....|....|...
OBP9_Seq_Fw-Primer                ----------------------------------------------------------
OBP9_Seq_Rv-Primer(Rev-Comp)      --------
OBP9_cds                          CAAATGCG
```

OBP 10

```
                                            10        20        30        40        50        60        70        80        90       100
                                  ....|....|....|....|....|....|....|....|....|....|....|....|....|....|....|....|....|....|....|....|
OBP10_Seq_Fw-Primer               ----------------------------------------------------------------------------------------------------
OBP10_Seq_Rv-Primer(Rev-Comp)     ----------------------------------------------------------------------------------------------------
OBP10_cds                         GTTTTAAAAATGTTATTTTGTTATAAAAGATTTTTAGAAATATTTTGAATGGGGAAAGCCTGTGAATAACCTTTGAATGAGGTGAATAGAACAATAGTTT

                                           110       120       130       140       150       160       170       180       190       200
                                  ....|....|....|....|....|....|....|....|....|....|....|....|....|....|....|....|....|....|....|....|
OBP10_Seq_Fw-Primer               ----------------------------------------------------------------------------------------------------
OBP10_Seq_Rv-Primer(Rev-Comp)     ----------------------------------------------------------------------------------------------------
OBP10_cds                         TCAAAACCAGTCAAAAGTTAATGGAAATTCAATTTGATGTTTTGCAAATTCACCTGGTTTTCATATCGAAGTTTTTCATCCATCAATATCAGCGTGAGAT

                                           210       220       230       240       250       260       270       280       290       300
                                  ....|....|....|....|....|....|....|....|....|....|....|....|....|....|....|....|....|....|....|....|
OBP10_Seq_Fw-Primer               ----------------------------------------------------------------------------------------------------
OBP10_Seq_Rv-Primer(Rev-Comp)     -------------------------------------------------------------------------------------------ACGTGAAGG
OBP10_cds                         TTCTTTCCCTAATTAAGCCGATCGAATGTTACTCTTCCAAGAACTGGCACAGACTGTCTTAACATTATTTCAATAATTTTTATTCATTTACACGTGAAGG

                                           310       320       330       340       350       360       370       380       390       400
                                  ....|....|....|....|....|....|....|....|....|....|....|....|....|....|....|....|....|....|....|....|
OBP10_Seq_Fw-Primer               -----------------------------------------AATAGAGACGCTTGCATTACCGAGACAGGTGCTGACAGAGAGGAGGTTA----------
OBP10_Seq_Rv-Primer(Rev-Comp)     CCCAAGAATTGACGGAAGCGCAAAAGCAGAAAGTCAGGGA------------------------------------------------------------
OBP10_cds                         CCCAAGAATTGACGGAAGCGCAAAAGCAGAAAGTCAGGGAGAATAGAGACGCTTGCATTACCGAGACAGGTGCTGACAGAGAGGAGGTTAATAAGGCTAA

                                           410       420       430       440       450       460       470       480       490       500
                                  ....|....|....|....|....|....|....|....|....|....|....|....|....|....|....|....|....|....|....|....|
OBP10_Seq_Fw-Primer               ----------------------------------------------------------------------------------------------------
OBP10_Seq_Rv-Primer(Rev-Comp)     ----------------------------------------------------------------------------------------------------
OBP10_cds                         TAAGGGCGAATGGGCTGACAATGCAAAGATCCGTTGTTTCACCCTCTGTATGCTGAAGAAATGGGGCATGATGAATGATGCCGGTGTATTAGATGAAGCT

                                           510       520       530       540       550       560       570       580       590       600
                                  ....|....|....|....|....|....|....|....|....|....|....|....|....|....|....|....|....|....|....|....|
OBP10_Seq_Fw-Primer               ----------------------------------------------------------------------------------------------------
OBP10_Seq_Rv-Primer(Rev-Comp)     ----------------------------------------------------------------------------------------------------
OBP10_cds                         GCAGCTCGCCAGAAGATGGGCTTGCAGATGAAGCCCGAGAAGGTCGAGGAGATCATGACCAAGTGCAAATATCTGAAGGGCGATACCGCTTGTGATACAG

                                           610       620       630       640       650       660       670       680       690       700
                                  ....|....|....|....|....|....|....|....|....|....|....|....|....|....|....|....|....|....|....|....|
OBP10_Seq_Fw-Primer               ----------------------------------------------------------------------------------------------------
OBP10_Seq_Rv-Primer(Rev-Comp)     ----------------------------------------------------------------------------------------------------
OBP10_cds                         CATACATGATGATGAAGTGCTACACTGACAACAGAGCAGTCACCGTCTAAGATTAACCCGAAATTTCTACTATACAAACATCGATGATTTTACTGATTTT

                                           710       720       730       740       750       760       770       780       790       800
                                  ....|....|....|....|....|....|....|....|....|....|....|....|....|....|....|....|....|....|....|....|
OBP10_Seq_Fw-Primer               ----------------------------------------------------------------------------------------------------
OBP10_Seq_Rv-Primer(Rev-Comp)     ----------------------------------------------------------------------------------------------------
OBP10_cds                         TTTTGAGTGCACATGATTTAATCTGTTCAATAAAGTTTTGGATCTCTCAGTTTTCCATTGGATTCTTCAATTCATTGAAATACAACATTTATTGGCGATG

                                           810       820       830       840       850       860       870       880       890       900
                                  ....|....|....|....|....|....|....|....|....|....|....|....|....|....|....|....|....|....|....|....|
OBP10_Seq_Fw-Primer               ----------------------------------------------------------------------------------------------------
OBP10_Seq_Rv-Primer(Rev-Comp)     ----------------------------------------------------------------------------------------------------
OBP10_cds                         ATTGAAATTTTCTTGAGCTACAATTTCATCAAACTTGATGTCTGTGCAGATAAATAAATTTTGATTTTTAGAAATATTGTCGCTGAGGAGTTGGCCTCTG
```

```
                                             910       920       930       940       950       960       970       980       990      1000
                                    ....|....|....|....|....|....|....|....|....|....|....|....|....|....|....|....|....|....|....|....|
OBP10_Seq_Fw-Primer                 ----------------------------------------------------------------------------------------------------
OBP10_Seq_Rv-Primer(Rev-Comp)       ----------------------------------------------------------------------------------------------------
OBP10_cds                           GCATAGTAATTAGATAGGCTATTGAGGCCCATCGGAAAAGTTGGTTGAACCAACCAGAGAAAATTCGCCATCGCTGATCAATTTTTGGACGGATAAATCG

                                            1010      1020      1030      1040      1050      1060      1070
                                    ....|....|....|....|....|....|....|....|....|....|....|....|....|....|.
OBP10_Seq_Fw-Primer                 -----------------------------------------------------------------------
OBP10_Seq_Rv-Primer(Rev-Comp)       ---------------------
OBP10_cds                           ATATTGGGTTATGGATTTGAA
```

OBP 11

```
                                              10        20        30        40        50        60        70        80        90       100
                                    ....|....|....|....|....|....|....|....|....|....|....|....|....|....|....|....|....|....|....|....|
OBP11_Seq_Fw-Primer                 ----------------------------------------------------------------------------------------------------
OBP11_Seq_Rv-Primer(Rev-Comp)       ----------------------------------------------------------------------------------------------------
OBP11_cds                           TATTGATCAAGACCCGCTAGTGCTCACAACAATCGAAAAATTGTCAACTAAATAACCATTTTTTTTTGCCTCAGGAATCGGTGATATTTCGAAAGTTTTC

                                             110       120       130       140       150       160       170       180       190       200
                                    ....|....|....|....|....|....|....|....|....|....|....|....|....|....|....|....|....|....|....|....|
OBP11_Seq_Fw-Primer                 ----------------------------------------------------------------------------------------------------
OBP11_Seq_Rv-Primer(Rev-Comp)       ----------------------------------------------------------------------------------------------------
OBP11_cds                           TTTAGAAAAATGTGATAATGTTGAAATTGGAGTTGAAATTGAAATACTCCCAGTAAATTCCTCAGTAAATTTTTATTTGAACGTCGGTTTTTCCCCAATT

                                             210       220       230       240       250       260       270       280       290       300
                                    ....|....|....|....|....|....|....|....|....|....|....|....|....|....|....|....|....|....|....|....|
OBP11_Seq_Fw-Primer                 ----------------------------------------------------------------------------------------------------
OBP11_Seq_Rv-Primer(Rev-Comp)       ----------------------------------------------------------------------------------------------------
OBP11_cds                           TTTCCTATATTACCTACACGTGGATAAGGTAGGGAAAGAGATAGAACTATCAATATCCCATTCAGTCTCATGCATAATTAACCACTTTAGAAGTTACTCT

                                             310       320       330       340       350       360       370       380       390       400
                                    ....|....|....|....|....|....|....|....|....|....|....|....|....|....|....|....|....|....|....|....|
OBP11_Seq_Fw-Primer                 ----------------------------------------------------------------------------------------------------
OBP11_Seq_Rv-Primer(Rev-Comp)       ----------------------------------------------------------------------------------------------------
OBP11_cds                           TGTTATTATGGTTGTCATAAGGTGTCAGAGGTTTATTAGGTGGTTTCTTACTACATCGAAGAACGTTTTTGATTTTCCATTATTCCTCTTGTTGATTCTA

                                             410       420       430       440       450       460       470       480       490       500
                                    ....|....|....|....|....|....|....|....|....|....|....|....|....|....|....|....|....|....|....|....|
OBP11_Seq_Fw-Primer                 ----------------------------------------------------------------------------------------------------
OBP11_Seq_Rv-Primer(Rev-Comp)       ----------------------------------------------------------------------------------------------------
OBP11_cds                           CATCCATACAGGAGGCAGGAATACATGTCGAAATATATTTTATATACATTAGCATCACAGAAAACACAAAGCTACCAATAATGTCGCTGTGGTGTGCAAA

                                             510       520       530       540       550       560       570       580       590       600
                                    ....|....|....|....|....|....|....|....|....|....|....|....|....|....|....|....|....|....|....|....|
OBP11_Seq_Fw-Primer                 ----------------------------------------------------------------------------------------------------
OBP11_Seq_Rv-Primer(Rev-Comp)       ----------------------------------------------------------------------------------------------------
OBP11_cds                           ATATCTGTAAAATATATGTGACAAATGACAAGTCAGGGAAATACATATATTTCACATCTTTTTCAATCATAAAATGATATATTTGAGGCCTATATGAGAT

                                             610       620       630       640       650       660       670       680       690       700
                                    ....|....|....|....|....|....|....|....|....|....|....|....|....|....|....|....|....|....|....|....|
OBP11_Seq_Fw-Primer                 ----------------------------------------------------------------------------------------------------
OBP11_Seq_Rv-Primer(Rev-Comp)       ----------------------------------------------------------------------------------------------------
OBP11_cds                           TTATCCTTCGACATATATTTCAATCAACCCAAAATGATCTGCCCCATATATAGGCTATATAGATAACATATGGAAGATATATTTTATATATTTTTTTAGT

                                             710       720       730       740       750       760       770       780       790       800
                                    ....|....|....|....|....|....|....|....|....|....|....|....|....|....|....|....|....|....|....|....|
OBP11_Seq_Fw-Primer                 ----------------------------------------------------------------------------------------------------
OBP11_Seq_Rv-Primer(Rev-Comp)       ----------------------------------------------------------------------------------------------------
OBP11_cds                           AATATGTGCCCAAAATCTTCGCTGTATATTGTTCCCTGTAGGCGAATACTTTAAGCGAAATAAATGCTGCTCAGCTTGAAAAAATGGCAGAACCTACGCT

                                             810       820       830       840       850       860       870       880       890       900
                                    ....|....|....|....|....|....|....|....|....|....|....|....|....|....|....|....|....|....|....|....|
OBP11_Seq_Fw-Primer                 ----------------------------------------------------------------------------------------------------
OBP11_Seq_Rv-Primer(Rev-Comp)       ----------------------------------------------------------------------------------------------------
OBP11_cds                           ACATCATCCCCATCGCTGGTGATATTCCAATTATCAATTATTAATAGAGCAGCGATTGCGAATTAGTATTAGACGACGTTTGCTCTGTTATCAGTATAGC

                                             910       920       930       940       950       960       970       980       990      1000
                                    ....|....|....|....|....|....|....|....|....|....|....|....|....|....|....|....|....|....|....|....|
OBP11_Seq_Fw-Primer                 -----------------------------------------------------------------------------------CGCGGAACTACCAACGC
OBP11_Seq_Rv-Primer(Rev-Comp)       ACTTCAGGACCAAATAGCCAGTATCACAGGGAGTGTTGCCCTTCAGATCCTTGCACTTCGTCATGATCTCTTCTACCCTTTCAC----------------
OBP11_cds                           ACTTCAGGACCAAATAGCCAGTATCACAGGGAGTGTTGCCCTTCAGATCCTTGCACTTCGTCATGATCTCTTCTACCCTTTCACGCGGAACTACCAACGC

                                            1010      1020      1030      1040      1050      1060      1070      1080      1090      1100
                                    ....|....|....|....|....|....|....|....|....|....|....|....|....|....|....|....|....|....|....|....|
OBP11_Seq_Fw-Primer                 CAATCTCTGCCGAGCCGCAGCTTCATTCAGTTGACCA---------------------------------------------------------------
OBP11_Seq_Rv-Primer(Rev-Comp)       ----------------------------------------------------------------------------------------------------
OBP11_cds                           CAATCTCTGCCGAGCCGCAGCTTCATTCAGTTGACCATCTTCACTCATCAAACGTAACCGCCTCATCATGCACAAAGCGAAACAACGGATTTGAGGGTTA

                                            1110      1120      1130      1140      1150      1160      1170      1180      1190      1200
                                    ....|....|....|....|....|....|....|....|....|....|....|....|....|....|....|....|....|....|....|....|
OBP11_Seq_Fw-Primer                 ----------------------------------------------------------------------------------------------------
OBP11_Seq_Rv-Primer(Rev-Comp)       ----------------------------------------------------------------------------------------------------
OBP11_cds                           TCAGCCCATTGGCCCTGACGAGCCCTGTCGACCTCGGCTCTGTTAGCATCTGTCTCTCTTATACAAATATCTCTATGCTCTCTAAGTCTCCGTCTTTGGG

                                            1210      1220      1230      1240      1250      1260      1270      1280      1290      1300
                                    ....|....|....|....|....|....|....|....|....|....|....|....|....|....|....|....|....|....|....|....|
OBP11_Seq_Fw-Primer                 ----------------------------------------------------------------------------------------------------
OBP11_Seq_Rv-Primer(Rev-Comp)       ----------------------------------------------------------------------------------------------
OBP11_cds                           CTTCGGTTAACTCCTGGGCCAGAGCACCAGCGAGACAGACGAAAATCACAACGACGAGGAATTTCATATTGTTTTTTCAATCGTTATAGACTCC

                                            1310      1320      1330      1340      1350      1360
                                    ....|....|....|....|....|....|....|....|....|....|....|....|....|...
OBP11_Seq_Fw-Primer                 --------------------------------------------------------------------
OBP11_Seq_Rv-Primer(Rev-Comp)
OBP11_cds
```

OBP 12

```
                                              10        20        30        40        50        60        70        80        90       100
                                    ....|....|....|....|....|....|....|....|....|....|....|....|....|....|....|....|....|....|....|....|
OBP12_Seq_Fw-Primer                 ----------------------------------------------------------------------------------------------------
OBP12_Seq_Rv-Primer(Rev-Comp)       --------------------------------------------------------------------------------------CTCATTGGTGCTCT
OBP12_cds                           CACTTTTGCAACTTGCTTTATCCTCCGAAAGCTATACAATTTAAAAAGTCAACATGAAAGTATTTGTGGTTATCCTCTCCACCTGTCTCATTGGTGCTCT

                                             110       120       130       140       150       160       170       180       190       200
                                    ....|....|....|....|....|....|....|....|....|....|....|....|....|....|....|....|....|....|....|....|
OBP12_Seq_Fw-Primer                 -------------------------AAGCAGAAAGTCAGGGAGAATAGAGACGCTTGCATTACCGAGACAGGA---------------------------
OBP12_Seq_Rv-Primer(Rev-Comp)       GGCCCAAGAATTGACGGAAGCGCAAAAGCA----------------------------------------------------------------------
OBP12_cds                           GGCCCAAGAATTGACGGAAGCGCAAAAGCAGAAAGTCAGGGAGAATAGAGACGCTTGCATTACCGAGACAGGTGCTGACAGAGAGGAGGTTAATAAGGCT

                                             210       220       230       240       250       260       270       280       290       300
                                    ....|....|....|....|....|....|....|....|....|....|....|....|....|....|....|....|....|....|....|....|
OBP12_Seq_Fw-Primer                 ----------------------------------------------------------------------------------------------------
OBP12_Seq_Rv-Primer(Rev-Comp)       ----------------------------------------------------------------------------------------------------
OBP12_cds                           AATAAGGGCGAATGGGCTGACAATGCAAAGATCCGTTGTTTCACCCTCTGTATGCTGAAGAAATGGGGCATGATGAATGATGCCGGTGTATTAGATGAAG

                                             310       320       330       340       350       360       370       380       390       400
                                    ....|....|....|....|....|....|....|....|....|....|....|....|....|....|....|....|....|....|....|....|
OBP12_Seq_Fw-Primer                 ----------------------------------------------------------------------------------------------------
OBP12_Seq_Rv-Primer(Rev-Comp)       ----------------------------------------------------------------------------------------------------
```

```
OBP12_cds                         CTGCAGCTCGCCAGAAGATGGGCTTGCAGATGAAGCCCGAGAAGGTCGAGGAGATCATGACCAAGTGCAAATATCTGAAGGGCGATACCGCTTGTGATAC

                                           410       420       430       440       450       460       470       480       490       500
                                  ....|....|....|....|....|....|....|....|....|....|....|....|....|....|....|....|....|....|....|....|
OBP12_Seq_Fw-Primer               ----------------------------------------------------------------------------------------------------
OBP12_Seq_Rv-Primer(Rev-Comp)     ----------------------------------------------------------------------------------------------------
OBP12_cds                         AGCATACATGATGATGAAGTGCTACACTGACAACAGAGCAGTCACCGTCTAAGATTAACCCGAAATTTCTACTATACAAACATCGATGATTTTACTGATT

                                           510       520       530       540       550       560       570       580       590       600
                                  ....|....|....|....|....|....|....|....|....|....|....|....|....|....|....|....|....|....|....|....|
OBP12_Seq_Fw-Primer               ----------------------------------------------------------------------------------------------------
OBP12_Seq_Rv-Primer(Rev-Comp)     ----------------------------------------------------------------------------------------------------
OBP12_cds                         TTTTTTGAGTGCACATGATTTAATCTGTTCAATAAAGTTTTGGATCTCTCAGTTTTCCATTGGATTCTTCAATTCATTGAAATACAACATTTATTGGCGA

                                           610       620       630       640       650       660       670       680       690       700
                                  ....|....|....|....|....|....|....|....|....|....|....|....|....|....|....|....|....|....|....|....|
OBP12_Seq_Fw-Primer               ----------------------------------------------------------------------------------------------------
OBP12_Seq_Rv-Primer(Rev-Comp)     ----------------------------------------------------------------------------------------------------
OBP12_cds                         TGATTGAAATTTTCTTGAGCTACAATTTCATCAAACTTGATGTCTGTGCAGATAAATAAATTTTGATTTTTAGAAATATTGTCGCTGAGGAGTTGGCCTC

                                           710       720       730       740       750       760       770       780       790       800
                                  ....|....|....|....|....|....|....|....|....|....|....|....|....|....|....|....|....|....|....|....|
OBP12_Seq_Fw-Primer               ----------------------------------------------------------------------------------------------------
OBP12_Seq_Rv-Primer(Rev-Comp)     ----------------------------------------------------------------------------------------------------
OBP12_cds                         TGGCATAGTAATTAGATAGGCTATTGAGGCCCATCGGAAAAGTTGGTTGAACCAACCAGAGAAAATTCGCCATCGCTGATCAATTTTTGGACGGATAAAT

                                           810       820       830       840       850       860
                                  ....|....|....|....|....|....|....|....|....|....|....|....|...
OBP12_Seq_Fw-Primer               ---------------------------------------------------------------
OBP12_Seq_Rv-Primer(Rev-Comp)     -----------------------
OBP12_cds                         CGATATTGGGTTATGGATTTGAA
```

OBP 14

```
                                           10        20        30        40        50        60        70        80        90       100
                                  ....|....|....|....|....|....|....|....|....|....|....|....|....|....|....|....|....|....|....|....|
OBP14_Seq_Fw-Primer               ----------------------------------------------------------------------------------------------------
OBP14_Seq_Rv-Primer(Rev-Comp)     ----------------------------------------------------------------------------------------------------
OBP14_cds                         GTTTTGCGGGAGATTTATACCCTATAGGATTGCTCGAGTATTTTTTATCATTTGTGCTCAAATCGTGAAATTCTCTTCGTCAATTAACTATATTTAATTA

                                           110       120       130       140       150       160       170       180       190       200
                                  ....|....|....|....|....|....|....|....|....|....|....|....|....|....|....|....|....|....|....|....|
OBP14_Seq_Fw-Primer               ---------------------------------------------------------------------------------------------------A
OBP14_Seq_Rv-Primer(Rev-Comp)     ----------------------------------------------------------------------------------------------------
OBP14_cds                         ACCCTATCATGAGGAAGTACGTTGGTTTCCTATGTCTAATTCTTCAGGTGTCAATTGGACACTGTGGGCCGGTTGGGCGACCCGATTTCGTCTCAGATGA

                                           210       220       230       240       250       260       270       280       290       300
                                  ....|....|....|....|....|....|....|....|....|....|....|....|....|....|....|....|....|....|....|....|
OBP14_Seq_Fw-Primer               AGATTGCATTGGCAGCAAGTGTTGTCAATGCATGTCAAACACAGACAGGCGTAGCTACAG----------------------------------------
OBP14_Seq_Rv-Primer(Rev-Comp)     ---------------------------ATGCATGTCAAACACAGACAGGCGTAGCTACAGCTGACATTGAGGCTGTCAGAAATGGACAATGGCTAAACTC
OBP14_cds                         AGATTGCATTGGCAGCAAGTGTTGTCAATGCATGTCAAACACAGACAGGCGTAGCTACAGCTGACATTGAGGCTGTCAGAAATGGACAATGGCTAAACTC

                                           310       320       330       340       350       360       370       380       390       400
                                  ....|....|....|....|....|....|....|....|....|....|....|....|....|....|....|....|....|....|....|....|
OBP14_Seq_Fw-Primer               ----------------------------------------------------------------------------------------------------
OBP14_Seq_Rv-Primer(Rev-Comp)     AACAC-----------------------------------------------------------------------------------------------
OBP14_cds                         AACACCTTTAAAGTGCTACATGTACTGCCTTTGGGAACAATTTGGTCTTGTCGATGACAAAAGAGAGCTCAGTCTCAACGGAATGCTAACATTTTTCCAA

                                           410       420       430       440       450       460       470       480       490       500
                                  ....|....|....|....|....|....|....|....|....|....|....|....|....|....|....|....|....|....|....|....|
OBP14_Seq_Fw-Primer               ----------------------------------------------------------------------------------------------------
OBP14_Seq_Rv-Primer(Rev-Comp)     ----------------------------------------------------------------------------------------------------
OBP14_cds                         CGATACCAGCCTACAGGGCAGAAGTGCAAACAGCGATCAGAGAGTGCAAGAAGATTGGTAAATTCTTCGGTATTTATTTTTTTATTATTATTTTCTCTTC

                                           510       520       530       540       550       560       570       580       590       600
                                  ....|....|....|....|....|....|....|....|....|....|....|....|....|....|....|....|....|....|....|....|
OBP14_Seq_Fw-Primer               ----------------------------------------------------------------------------------------------------
OBP14_Seq_Rv-Primer(Rev-Comp)     ----------------------------------------------------------------------------------------------------
OBP14_cds                         AGTTGGTTATTTTTTAATAATTTCCCTACAATATCCACATTACTAACTATCCTGACTTGGCTTATTGCTCAGCTGATGGAGACAACTGCCAGTACGCCTA

                                           610       620       630       640       650       660       670       680       690       700
                                  ....|....|....|....|....|....|....|....|....|....|....|....|....|....|....|....|....|....|....|....|
OBP14_Seq_Fw-Primer               ----------------------------------------------------------------------------------------------------
OBP14_Seq_Rv-Primer(Rev-Comp)     ----------------------------------------------------------------------------------------------------
OBP14_cds                         CACCTTCAACCTGTGCTATTCAAAACTATCCCCAAGGGTACGAAACAATATTAAATATAATTTTCCCTTCAATAAATCTGCCCTAAATCTACACTAATAT

                                           710       720       730       740       750       760
                                  ....|....|....|....|....|....|....|....|....|....|....|....|....|.
OBP14_Seq_Fw-Primer               -----------------------
OBP14_Seq_Rv-Primer(Rev-Comp)     ------------------------------------------------------------------
OBP14_cds                         CCATTCCTTATTTGTTTTTCAGACTTACTATTTGTTCTAACTGCCGAAAACCGACCGAATACGTGT
```

OBP 17

```
                                           10        20        30        40        50        60        70        80        90       100
                                  ....|....|....|....|....|....|....|....|....|....|....|....|....|....|....|....|....|....|....|....|
OBP17_Seq_Fw-Primer               ----------------------------------------------------------------------------------------------------
OBP17_Seq_Rv-Primer(Rev-Comp)     ----------------------------------------------------------------------------------------------------
OBP17_cds                         GGCGGATAATTTATTGTGTGTGTTGCCATGCCAGAAAAGGTTTCCATTCATAATTTGGCAATGTACACGTTTTTGGAATTTAAAACATTTTTCGGCATTG

                                           110       120       130       140       150       160       170       180       190       200
                                  ....|....|....|....|....|....|....|....|....|....|....|....|....|....|....|....|....|....|....|....|
OBP17_Seq_Fw-Primer               ----------------------------------------------------------------------------------------------------
OBP17_Seq_Rv-Primer(Rev-Comp)     ----------------------------------------------------------------------------------------------------
OBP17_cds                         ATTGATTAATTGATTGAGGAATTTTCCATTGAGCCATTGCGTTGCCACTGATTAATGATCCTTTCCGTTGAGGAGGACAAAGCAGCTACGCTACTATTTG

                                           210       220       230       240       250       260       270       280       290       300
                                  ....|....|....|....|....|....|....|....|....|....|....|....|....|....|....|....|....|....|....|....|
OBP17_Seq_Fw-Primer               ----------------------------------------------------------------------------------------------------
OBP17_Seq_Rv-Primer(Rev-Comp)     ----------------------------------------------------------------------------------------------------
OBP17_cds                         TTGACATTGAGTTTTGCTCAGTATATTGTTGGTCTTACCCGAAAAGCAGAGTTGGAATATTGTTCTCGTACATGCAGACGAATAGCAAATGAGCTGTTGT

                                           310       320       330       340       350       360       370       380       390       400
                                  ....|....|....|....|....|....|....|....|....|....|....|....|....|....|....|....|....|....|....|....|
OBP17_Seq_Fw-Primer               ----------------------------------------------------------------------------------------------------
OBP17_Seq_Rv-Primer(Rev-Comp)     ----------------------------------------------------------------------------------------------------
OBP17_cds                         GCATTTATCAGGATCTCTTCTAGCACTGCACGTTTTGATAGCATCTGTCAGCTGTTGTGCGAAGTCAGCGTCCTGCGGTATTTTAGTTACAGCCAATTCA

                                           410       420       430       440       450       460       470       480       490       500
                                  ....|....|....|....|....|....|....|....|....|....|....|....|....|....|....|....|....|....|....|....|
OBP17_Seq_Fw-Primer               ----------------------------------------------------------------------------------------------------
OBP17_Seq_Rv-Primer(Rev-Comp)     ---------------------------------------------------------------------------------------TCTCCATTCAACA
OBP17_cds                         TCATTGAATGAACCATCTGGCTTTAGCAATTGGAATTCTTCAAGGAGACAAGCAGCAAAGCAGTTGAAATGTGGATCATCAACAATTTCTCCATTCAACA

                                           510       520       530       540       550       560       570       580       590       600
                                  ....|....|....|....|....|....|....|....|....|....|....|....|....|....|....|....|....|....|....|....|
OBP17_Seq_Fw-Primer               --------------------------TCCTCACTGATTCTACATTTTTCTACTGCCCTCTGAAAGCGCACGATATCTGCA--------------------
OBP17_Seq_Rv-Primer(Rev-Comp)     CACGCTCAAGTACTTCATCACTAACATCCTCAC-------------------------------------------------------------------
OBP17_cds                         CACGCTCAAGTACTTCATCACTAACATCCTCACTGATTCTACATTTTTCTACTGCCCTCTGAAAGCGCACGATATCTGCAGGTCTGAAGGCCCCAGCTGC

                                           610       620       630       640       650       660       670
                                  ....|....|....|....|....|....|....|....|....|....|....|....|....|....|....
OBP17_Seq_Fw-Primer               ------------------------------------------------------------------------------
```

```
OBP17_Seq_Rv-Primer(Rev-Comp)   ----------------------------------------
OBP17_cds                       AAATGCAACGTGAAAGATCAGTAGAATCACGAGAAGTTTC
```

NPC2a

```
                                        10        20        30        40        50        60        70        80        90       100
                                ....|....|....|....|....|....|....|....|....|....|....|....|....|....|....|....|....|....|....|....|
NPC2a_Seq_Fw-Primer             -------------------------------------------------------------------------------------------GGGTCCGCA
NPC2a_Seq_Rv-Primer(Rev-Comp)   ---------------------------------TTGTGCTTTCGTTGTGTGCTTGTCGACGCTAGTCAGTGCTGCTAAGTTCAGGGATTGCGGGTCCGCA
NPC2a_cds                       CAGACACCAAGGAGGCAAGATGTCTCGAATTATTTGTGCTTTCGTTGTGTGCTTGTCGACGCTAGTCAGTGCTGCTAAGTTCAGGGATTGCGGGTCCGCA

                                       110       120       130       140       150       160       170       180       190       200
                                ....|....|....|....|....|....|....|....|....|....|....|....|....|....|....|....|....|....|....|....|
NPC2a_Seq_Fw-Primer             GTGGGAAAGTACACGTCTGTTACTATCAGTAACTGTGGAGCCAGTGATAGCGAG----------------------------------------------
NPC2a_Seq_Rv-Primer(Rev-Comp)   GTGGGAAAGTACAC--------------------------------------------------------------------------------------
NPC2a_cds                       GTGGGAAAGTACACGTCTGTTACTATCAGTAACTGTGGAGCCAGTGATAGCGAGTGTATTCTCACCAGAGGATCTAATGCAACCATTGAAATATCATTCA

                                       210       220       230       240       250       260       270       280       290       300
                                ....|....|....|....|....|....|....|....|....|....|....|....|....|....|....|....|....|....|....|....|
NPC2a_Seq_Fw-Primer             ----------------------------------------------------------------------------------------------------
NPC2a_Seq_Rv-Primer(Rev-Comp)   ----------------------------------------------------------------------------------------------------
NPC2a_cds                       ACACCGACGAGTCCGCCGATGCTGTTACAGCAGTTGTCCATGGAATTGTAGCTAGTGTTCCCATGCCATATCCAATATCCCATCCTGATGCTTGTGCTAA

                                       310       320       330       340       350       360       370       380       390       400
                                ....|....|....|....|....|....|....|....|....|....|....|....|....|....|....|....|....|....|....|....|
NPC2a_Seq_Fw-Primer             ----------------------------------------------------------------------------------------------------
NPC2a_Seq_Rv-Primer(Rev-Comp)   ----------------------------------------------------------------------------------------------------
NPC2a_cds                       TCCGGACACTGGGATAACATGTCCTTTGAAGAAGGGTGGATCATACTCATACAGAAAAACTTTTCCCGTTTTAGCTCAATATCCTAAGGTGAGGGTTCAA

                                       410       420       430       440       450       460       470       480       490       500
                                ....|....|....|....|....|....|....|....|....|....|....|....|....|....|....|....|....|....|....|....|
NPC2a_Seq_Fw-Primer             ----------------------------------------------------------------------------------------------------
NPC2a_Seq_Rv-Primer(Rev-Comp)   ----------------------------------------------------------------------------------------------------
NPC2a_cds                       GTCAAATGGGAATTGCAAAATGAAAAGATGCAGGATATTATATGCATCTTGATTCCAGCAAAAATTCAGTAAATAATCAAGAACTGAATTTTTCAATGCG

                                       510       520       530       540       550       560       570       580       590       600
                                ....|....|....|....|....|....|....|....|....|....|....|....|....|....|....|....|....|....|....|....|
NPC2a_Seq_Fw-Primer             ----------------------------------------------------------------------------------------------------
NPC2a_Seq_Rv-Primer(Rev-Comp)   -------------------------------------------------------------------------------------
NPC2a_cds                       TCGACAGCAATAGACTGAACGTATCCCACTACTTTTTGCTTGTACTAATTGTTGCTAATCACACAAAGATAAATAGAAATTTCAG

                                       610       620       630
                                ....|....|....|....|....|....|....|....
NPC2a_Seq_Fw-Primer             ---------------------------------------
NPC2a_Seq_Rv-Primer(Rev-Comp)
NPC2a_cds
```

NPC2b

```
                                        10        20        30        40        50        60        70        80        90       100
                                ....|....|....|....|....|....|....|....|....|....|....|....|....|....|....|....|....|....|....|....|
NPC2b_Seq_Fw-Primer             ----------------------------------------------------------------------------------------------------
NPC2b_Seq_Rv-Primer(Rev-Comp)   -------------------------------------------------------GAAATGTTGGGACTCAAGGTATTGGTTATCGTGGGCTTCTGTGTG
NPC2b_cds                       AAACTATATAAGGGAATTTCGTTGGGGGGAGCTCAGCTTACGGTGATAATCCAAGGAAATGTTGGGACTCAAGGTATTGGTTATCGTGGGCTTCTGTGTG

                                       110       120       130       140       150       160       170       180       190       200
                                ....|....|....|....|....|....|....|....|....|....|....|....|....|....|....|....|....|....|....|....|
NPC2b_Seq_Fw-Primer             ----------------------CATTCACCAAGTGCAAGACGGGTGGGCCACCGCCGGAAAGCCTGAGGATCAAGGGGTGCAATA---------------
NPC2b_Seq_Rv-Primer(Rev-Comp)   GCTGCTTCCCTGCAGGATACTCCATTCACCA---------------------------------------------------------------------
NPC2b_cds                       GCTGCTTCCCTGCAGGATACTCCATTCACCAAGTGCAAGACGGGTGGGCCACCGCCGGAAAGCCTGAGGATCAAGGGGTGCAATACGACACCCTGTAAAT

                                       210       220       230       240       250       260       270       280       290       300
                                ....|....|....|....|....|....|....|....|....|....|....|....|....|....|....|....|....|....|....|....|
NPC2b_Seq_Fw-Primer             ----------------------------------------------------------------------------------------------------
NPC2b_Seq_Rv-Primer(Rev-Comp)   ----------------------------------------------------------------------------------------------------
NPC2b_cds                       TCATCAAGGGGACGGATATCAAGGCTGAGTGGGATTTCAATGTCGTTGCTGACACAGATGATCTCCACCCAAAAGTCCTCGTCAAAGTCGCGGGCTTCAC

                                       310       320       330       340       350       360       370       380       390       400
                                ....|....|....|....|....|....|....|....|....|....|....|....|....|....|....|....|....|....|....|....|
NPC2b_Seq_Fw-Primer             ----------------------------------------------------------------------------------------------------
NPC2b_Seq_Rv-Primer(Rev-Comp)   ----------------------------------------------------------------------------------------------------
NPC2b_cds                       CATCGACTACCCTCTACCTGAGCAGGACGCCTGCAAGTCCCTCACGAACGGTGAATGTCCATTGGAGAAGGGGGAGTTGGTCTCATATGGGCTGAAGATG

                                       410       420       430       440       450       460       470       480       490       500
                                ....|....|....|....|....|....|....|....|....|....|....|....|....|....|....|....|....|....|....|....|
NPC2b_Seq_Fw-Primer             ----------------------------------------------------------------------------------------------------
NPC2b_Seq_Rv-Primer(Rev-Comp)   ----------------------------------------------------------------------------------------------------
NPC2b_cds                       CCGATCCTCAAAATGTACCCTTCCACCAAACTCCACTTGACGTTTTCTCTGGTAGATCAGCATAAGAACACCCAGGTCTGCTTCGAAGTGGATGCTGCAG

                                       510       520       530       540       550       560       570       580       590       600
                                ....|....|....|....|....|....|....|....|....|....|....|....|....|....|....|....|....|....|....|....|
NPC2b_Seq_Fw-Primer             ----------------------------------------------------------------------------------------------------
NPC2b_Seq_Rv-Primer(Rev-Comp)   ----------------------------------------------------------------------------------------------------
NPC2b_cds                       TCATTGATTAAAACCTCTGAATAACAATGTCGCTCAAGTAATTAAATATTTAATTGGTCATTAATTTTATTGTAATAAATTCGATGGAAGGAGAAGAGAT

                                       610       620       630       640       650       660       670       680       690       700
                                ....|....|....|....|....|....|....|....|....|....|....|....|....|....|....|....|....|....|....|....|
NPC2b_Seq_Fw-Primer             ----------------------------------------------------------------------------------------------------
NPC2b_Seq_Rv-Primer(Rev-Comp)   ----------------------------------------------------------------------------------------------------
NPC2b_cds                       TTGCTGTCGTCCATTCATTGATTCATCCAATTAATTTTTAAATTAAATTAATCATTTTTTGTTATGGGAGCAAATGTTGGGTTTTTGGTCATAAAAAATA

                                       710       720       730       740       750       760       770       780       790
                                ....|....|....|....|....|....|....|....|....|....|....|....|....|....|....|....|....|....|....|....
NPC2b_Seq_Fw-Primer             ---------------------------------------------------------------------------------------------------
NPC2b_Seq_Rv-Primer(Rev-Comp)   ---------------------------------------------
NPC2b_cds                       GAGAGGTATTTTCCATTGAATTGTTGATAAAAAATAAAATGTTGC
```

ESR16

```
                                        10        20        30        40        50        60        70        80        90       100
                                ....|....|....|....|....|....|....|....|....|....|....|....|....|....|....|....|....|....|....|....|
ESR16_Seq_Fw-Primer             ----------------------------------------------------------------------------------------------------
ESR16_Seq_Rv-Primer(Rev-Comp)   ----------------------------------------------------------------------------------------------------
ESR16_cds                       GAAGGTTCAAGCGACCGGGACAGCCGCTCTGCCGGACAGTGATCACGTGAAAGTCTGTTTGTACGGAACGAAAATAATTACACTGCAGTTCTACTCCTCA

                                       110       120       130       140       150       160       170       180       190       200
                                ....|....|....|....|....|....|....|....|....|....|....|....|....|....|....|....|....|....|....|....|
ESR16_Seq_Fw-Primer             ----------------------------------------------------------------------------------------------------
ESR16_Seq_Rv-Primer(Rev-Comp)   ----------------------------------------------------------------------------------------------------
ESR16_cds                       GAAGGCACTAAAACTTTCACTGAGATGTTTGGAAAAACGGCGATTCTATTTACAACTGTCCTGTGTTTTGTGACTTTTGGAGAGTCAACGGATGTTCTCA

                                       210       220       230       240       250       260       270       280       290       300
                                ....|....|....|....|....|....|....|....|....|....|....|....|....|....|....|....|....|....|....|....|
ESR16_Seq_Fw-Primer             ----------------------------------------------------------------------------------------------------
ESR16_Seq_Rv-Primer(Rev-Comp)   ----------------------------------------------------------------------------------------------------
ESR16_cds                       AGTGCAAAAATGGTGGAAGCTTCGATACTATCAAGTCCATCGTAATAACAAAATGCGAACAGCCACCATGCCTATTAAAGCGTGGGACTAGGGTGGAACT

                                       310       320       330       340       350       360       370       380       390       400
                                ....|....|....|....|....|....|....|....|....|....|....|....|....|....|....|....|....|....|....|....|
ESR16_Seq_Fw-Primer             ----------------------------------------------------------------------------------------------------
ESR16_Seq_Rv-Primer(Rev-Comp)   ----------------------------------------------------------------------------------------------------
ESR16_cds                       GGTTCAACAATTTGTTCCCGAGAAAAATGTTGAAAAACTTACCACATCTGTCCATGCAACACTATTGGGAGTACCTCTGCCATTCGTTGGAGTCGATGGC
```

```
                                       410       420       430       440       450       460       470       480       490       500
                                ....|....|....|....|....|....|....|....|....|....|....|....|....|....|....|....|....|....|....|....|
ESR16_Seq_Fw-Primer             ------------------------------------------------------------------------------------------CCTGTTCTTA
ESR16_Seq_Rv-Primer(Rev-Comp)   ------------------------------------------GGATGTCCACTCAAAGCAGGCACCCAGTATTATTATAAAAATGGATTTCCTGTTCTTA
ESR16_cds                       ACTGACGCATGCCCAAATATTTTCGATGCCGATGGAAAACCTGGATGTCCACTCAAAGCAGGCACCCAGTATTATTATAAAAATGGATTTCCTGTTCTTA

                                       510       520       530       540       550       560       570       580       590       600
                                ....|....|....|....|....|....|....|....|....|....|....|....|....|....|....|....|....|....|....|....|
ESR16_Seq_Fw-Primer             ATTTTTACCCCACGGTCGACCTGGTCGTTGCCTGGGCACTGCTGGAAAACAATGTGCCA-----------------------------------------
ESR16_Seq_Rv-Primer(Rev-Comp)   ATTTTTACCC------------------------------------------------------------------------------------------
ESR16_cds                       ATTTTTACCCCACGGTCGACCTGGTCGTTGCCTGGGCACTGCTGGAAAACAATGTGCCACTCACGTGTTTCGAAATTCCAGCAAAAATCATTCGTTAAAA

                                       610       620       630       640       650       660
                                ....|....|....|....|....|....|....|....|....|....|....|....|..
ESR16_Seq_Fw-Primer             --------------------------------------------------------------
ESR16_Seq_Rv-Primer(Rev-Comp)   ------------
ESR16_cds                       ATACCATTTAAT
```

MD-2

```
                                        10        20        30        40        50        60        70        80        90       100
                                ....|....|....|....|....|....|....|....|....|....|....|....|....|....|....|....|....|....|....|....|
MD-2_Seq_Fw-Primer              ----------------------------------------------------------------------------------------------------
MD-2_Seq_Rv-Primer(Rev-Comp)    ----------------------------------------------------------------------------------------------------
MD-2_cds                        CTTAAACACCACATGTTCACAGGAAAACACCTCGAATAAACCCCTGAAATGACGTCTGAACTAGAACGAAAAACCTCACTTGATTCAATTGGTCTTTAAA

                                       110       120       130       140       150       160       170       180       190       200
                                ....|....|....|....|....|....|....|....|....|....|....|....|....|....|....|....|....|....|....|....|
MD-2_Seq_Fw-Primer              ----------------------------------------------------------------------------------------------------
MD-2_Seq_Rv-Primer(Rev-Comp)    ----------------------------------------------------------------------------------------------------
MD-2_cds                        TTTTTTTTAAGACTTCAAAAATCGTTACACTGAATTTCTTTGATCAATTCGATCAATTCTCATCAAGACCCAAAGAAAAATAACAAATCCCCACAAAAAA

                                       210       220       230       240       250       260       270       280       290       300
                                ....|....|....|....|....|....|....|....|....|....|....|....|....|....|....|....|....|....|....|....|
MD-2_Seq_Fw-Primer              ----------------------------------------------------------------------------------------------------
MD-2_Seq_Rv-Primer(Rev-Comp)    ----------------------------------------------------------------------------------------------------
MD-2_cds                        AAATATCTAACTGTTATAAAGGCGATTCTCACTTCATTATTGATCACATCGATTCATCTGAAAGTAATTCAAGTTCGAAAATTGGATTTTTGTTGTGTTT

                                       310       320       330       340       350       360       370       380       390       400
                                ....|....|....|....|....|....|....|....|....|....|....|....|....|....|....|....|....|....|....|....|
MD-2_Seq_Fw-Primer              ----------------------------------------------------------------------------------------------------
MD-2_Seq_Rv-Primer(Rev-Comp)    ----------------------------------------------------------------------------------------------------
MD-2_cds                        ACAAACGACCAGACATGTAAATACATTCACAGAATTGAAAAAAAAATTATGTTCATTTGACGAGCTTAATCTGGAAGACGATGCAGCACTCCTGCTCCTG

                                       410       420       430       440       450       460       470       480       490       500
                                ....|....|....|....|....|....|....|....|....|....|....|....|....|....|....|....|....|....|....|....|
MD-2_Seq_Fw-Primer              ----------------------------------------------------------------------------------------------------
MD-2_Seq_Rv-Primer(Rev-Comp)    ----------------------------------------------------------------------------------------------------
MD-2_cds                        CTGATTCCACAGTTTCCACTTGACATCGTAGCTCCTAGCTGGGTATTTGTGGTCGATGTGCAGACTCATTTTATAAGTCTGTTTGGTATCGGGGTTAACT

                                       510       520       530       540       550       560       570       580       590       600
                                ....|....|....|....|....|....|....|....|....|....|....|....|....|....|....|....|....|....|....|....|
MD-2_Seq_Fw-Primer              ----------------------------------------------------------------------------------------------------
MD-2_Seq_Rv-Primer(Rev-Comp)    ----------------------------------------------------------------------------------------------------
MD-2_cds                        GGACAGGGGGTGGACAAGCAGGCGTCGGTTGTCATCCCCAGGAAGGGTAGGTCCACCAGGCTATTGGCCCAGTAAGCTCGCCCTTGCAGCGTGTTAGCCG

                                       610       620       630       640       650       660       670       680       690       700
                                ....|....|....|....|....|....|....|....|....|....|....|....|....|....|....|....|....|....|....|....|
MD-2_Seq_Fw-Primer              ----------------------------------------------------------------------------------------------------
MD-2_Seq_Rv-Primer(Rev-Comp)    -------------------------------------------------------------------------------------------------CAA
MD-2_cds                        AAAAGTTTGTTGAGTAGTCGAATTCAATGCTGGCATCAGTTCCTCGTTTCACTCGACAGGGTTTAGCCGCTGCAGACTCTGGACAGGGCTTTATACGCAA

                                       710       720       730       740       750       760       770       780       790       800
                                ....|....|....|....|....|....|....|....|....|....|....|....|....|....|....|....|....|....|....|....|
MD-2_Seq_Fw-Primer              ----------------------------CCTTCGGGGTATACGCAGGGTTGAAATTGCACGACTTCAGCCCTTACCAGGGCGACACATACAA--------
MD-2_Seq_Rv-Primer(Rev-Comp)    TTCGTGGACTGTGCAGTTGATAGGAGTTCCT---------------------------------------------------------------------
MD-2_cds                        TTCGTGGACTGTGCAGTTGATAGGAGTTCCTTCGGGGTATACGCAGGGTTGAAATTGCACGACTTCAGCCCTTACCAGGGCGACACATACAACTCCAAAA

                                       810       820       830       840       850       860       870       880       890
                                ....|....|....|....|....|....|....|....|....|....|....|....|....|....|....|....|....|....|.
MD-2_Seq_Fw-Primer              -------------------------------------------------------------------------------------------
MD-2_Seq_Rv-Primer(Rev-Comp)    ---------------------------------------------------------------
MD-2_cds                        ATCACCAAAAAGCTCTTCATTGTTATTCTAATTGATATTTTTTTCGATCTCCACGGGATGAGC
```
